# Supplementary material for: ESRP1-regulated isoform switching of LRRFIP2 determines metastasis of gastric cancer
Source: Nat Commun. 2022 Oct 28;13:6274. doi: 10.1038/s41467-022-33786-9 (PMC9616898; doi:10.1038/s41467-022-33786-9)
Supplement: Supplementary file 1 — Supplementary Information [file 41467_2022_33786_MOESM1_ESM.pdf]

Supplementary Fig. 1

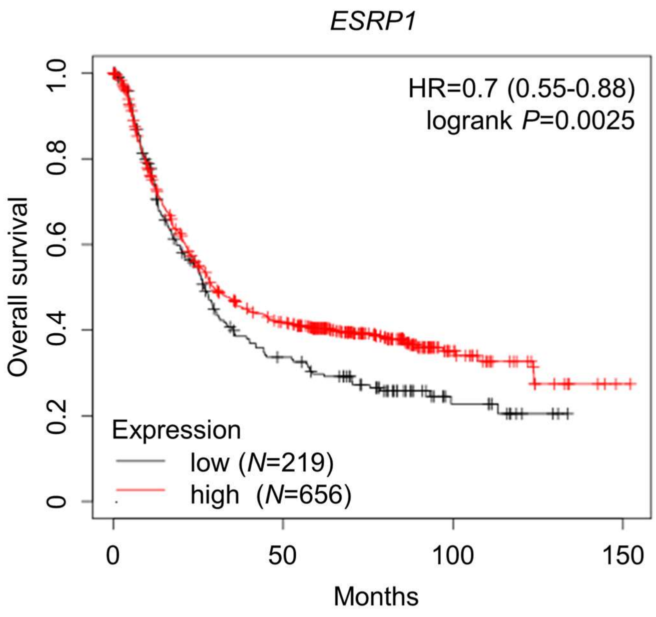

Supplementary Fig. 1. *ESRP1* expression is positively correlated with the overall survival times of gastric cancer patients. Kaplan-Meier analysis showing overall survival depending on *ESRP1* expression levels from public meta-analysis data (*N*=875). *P* values were calculated using a log-rank test.

Supplementary Fig. 2

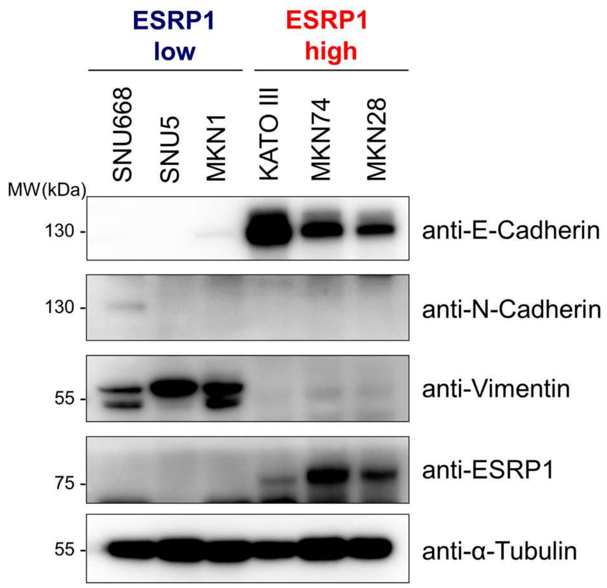

Supplementary Fig. 2. ESRP1 expression is correlated with the expression of EMT markers in gastric cancer cells. Immunoblot analysis of EMT markers in ESRP1-low gastric cancer cell lines (SNU668, SNU5 and MKN1) and ESRP1-high gastric cancer cell lines (KATOIII, MKN74 and MKN28). The representative results were obtained from at least two independent experiments.

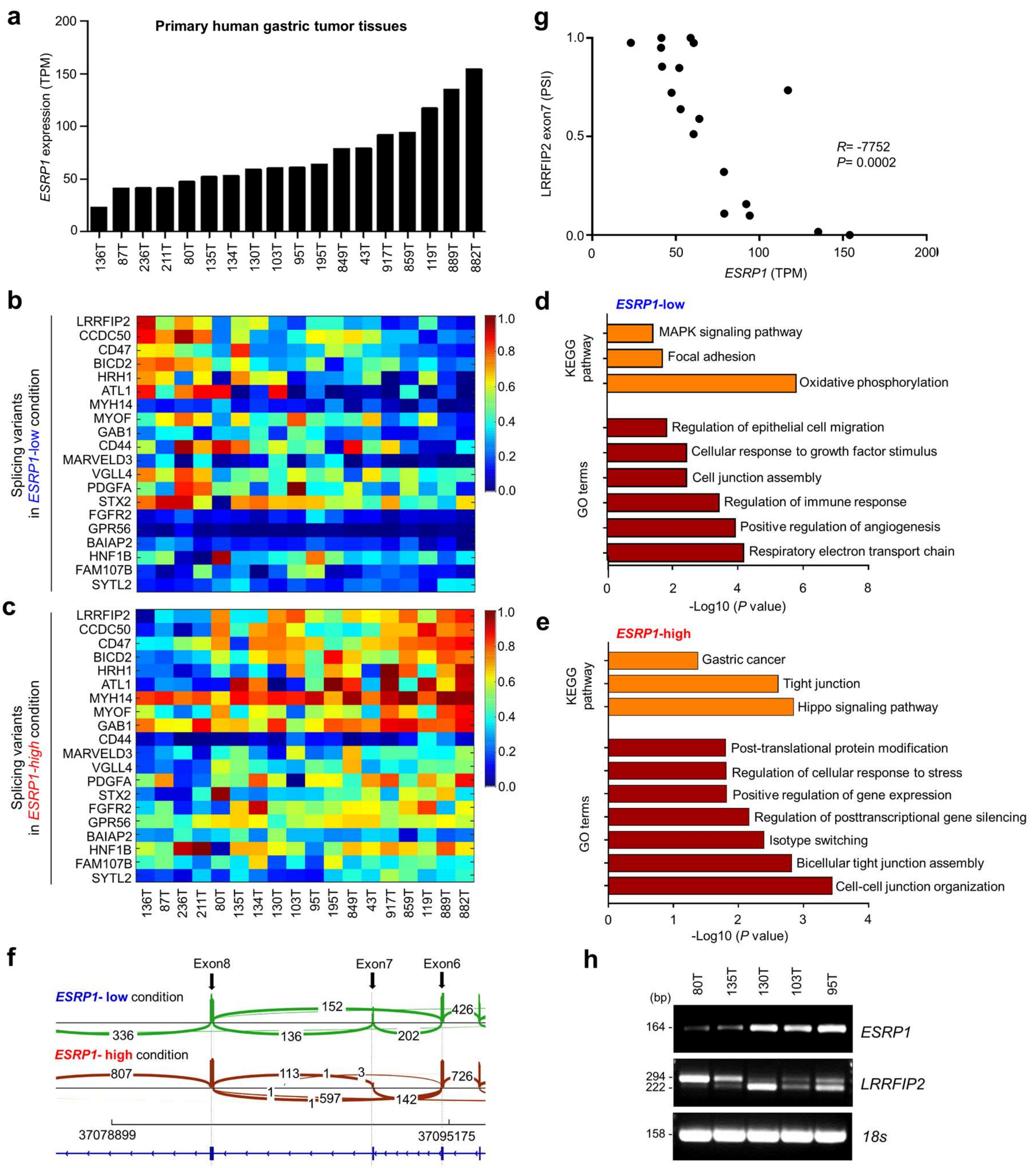

Supplementary Fig. 3. The relative frequencies of *LRRFIP2* splicing are significantly associated with the expression levels of *ESRP1* in gastric cancer patient tissues. a Bar graph showing the expression levels of *ESRP1* differentially expressed in the gastric cancer tissues. **b, c** Heatmaps illustrating relative TPM of 20 splicing variants alternatively expressed in **b** *ESRP1*-low and **c** -high conditions. **d, e** KEGG pathways and GO terms enriched in differentially expressed genes upregulated in **b** and **c**. **f** Sashimi plots indicate exon usage and splicing of LRRFIP2 in ESRP1-low and -high conditions. Arches and numbers represent RNA-seq reads at exon–exon junctions. **g** Pearson's correlation analysis between the PSI value of LRRFIP2 exon 7 and ESRP1 expression (TPM) in 18 patient tissue samples (*R*= -0.7724, *P*= 0.0002). **h** RT-PCR result showing expression levels of *LRRFIP2* variant 2 and variant 3 in gastric cancer tissues each with low or high expression level of ESRP1. The representative results were obtained from at least two independent experiments. Source data are provided in the Source Data file.

Supplementary Fig. 4

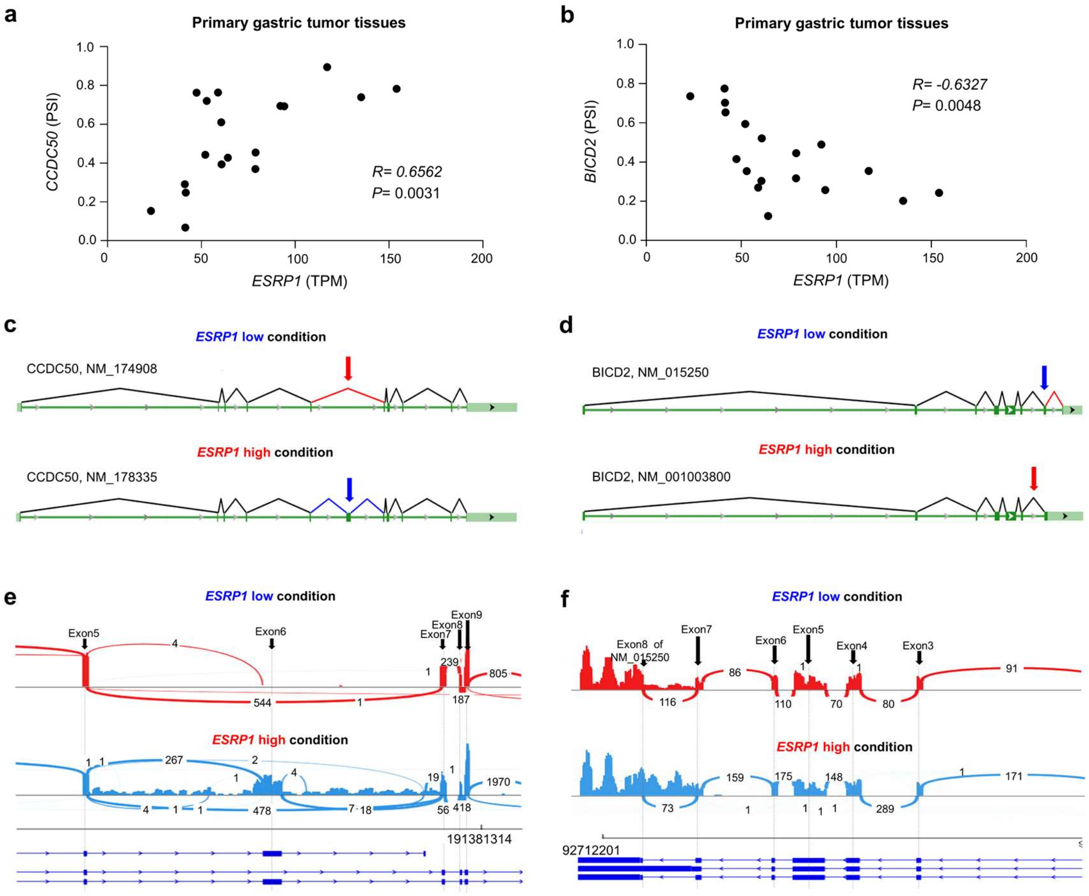

Supplementary Fig. 4. Splicing variants of CCDC50 and BICD2 differentially display exon skipping events in ESRP1-low and ESRP1-high conditions. a Pearson's correlation analysis between the PSI of *CCDC50* (NM_178335) exon 6 in the association of *ESRP1*(TPM) from **Supplementary Fig. 2b** and **c** (*R*=0.6562, *P*=0.0031). **b** Pearson's correlation analysis between the PSI of *BICD2* (NM_015250) exon 8 in the association of *ESRP1*(TPM) from **Supplementary Fig. 2b** and **c** (*R*=-0.6327, *P*=0.0048). **c, d** Schematic representation of human **c** CCDC50 and **d** BICD2 isoforms differentially expressed in ESRP1-low condition and ESRP1-high condition. The arrows point at the exons whose alternative splicing frequencies are associated with the expression level of ESRP1. **e**, **f** Sashimi plots indicate exon usage and splicing of **e** CCDC50 and **f** BICD2 in ESRP1-low and -high conditions. Arches and numbers represent RNA-seq reads at exon–exon junctions. Source data are provided in the Source Data file.

Supplementary Fig. 5

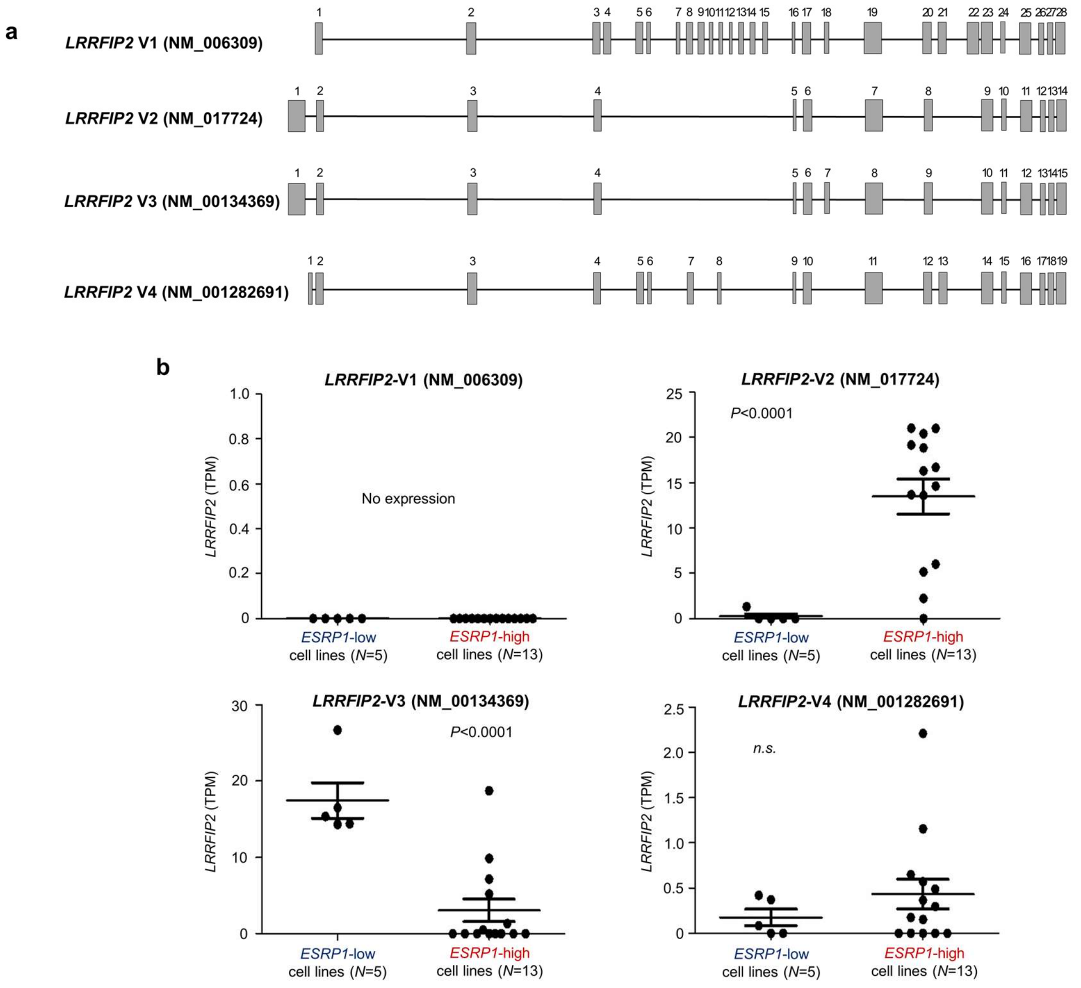

Supplementary Fig. 5. *LRRFIP2* variants 2 and 3 are highly expressed in the 18 gastric cell lines and their expression levels are significantly correlated with those of *ESRP1*. a Schematic representation of four variants of LRRFIP2. Grey boxes represent exons. **b** Dotplot derived from TPM profiles of four alternatively spliced isoforms under *ESRP1*-low condition (*N*=5) and *ESRP1*-high condition (*N*=13) from **Fig. 1b** and **c**. All *P* values were calculated by unpaired two-tailed Student's t tests. These data represent the mean ± S.D. Source data are provided in the Source Data file.

Supplementary Fig. 6

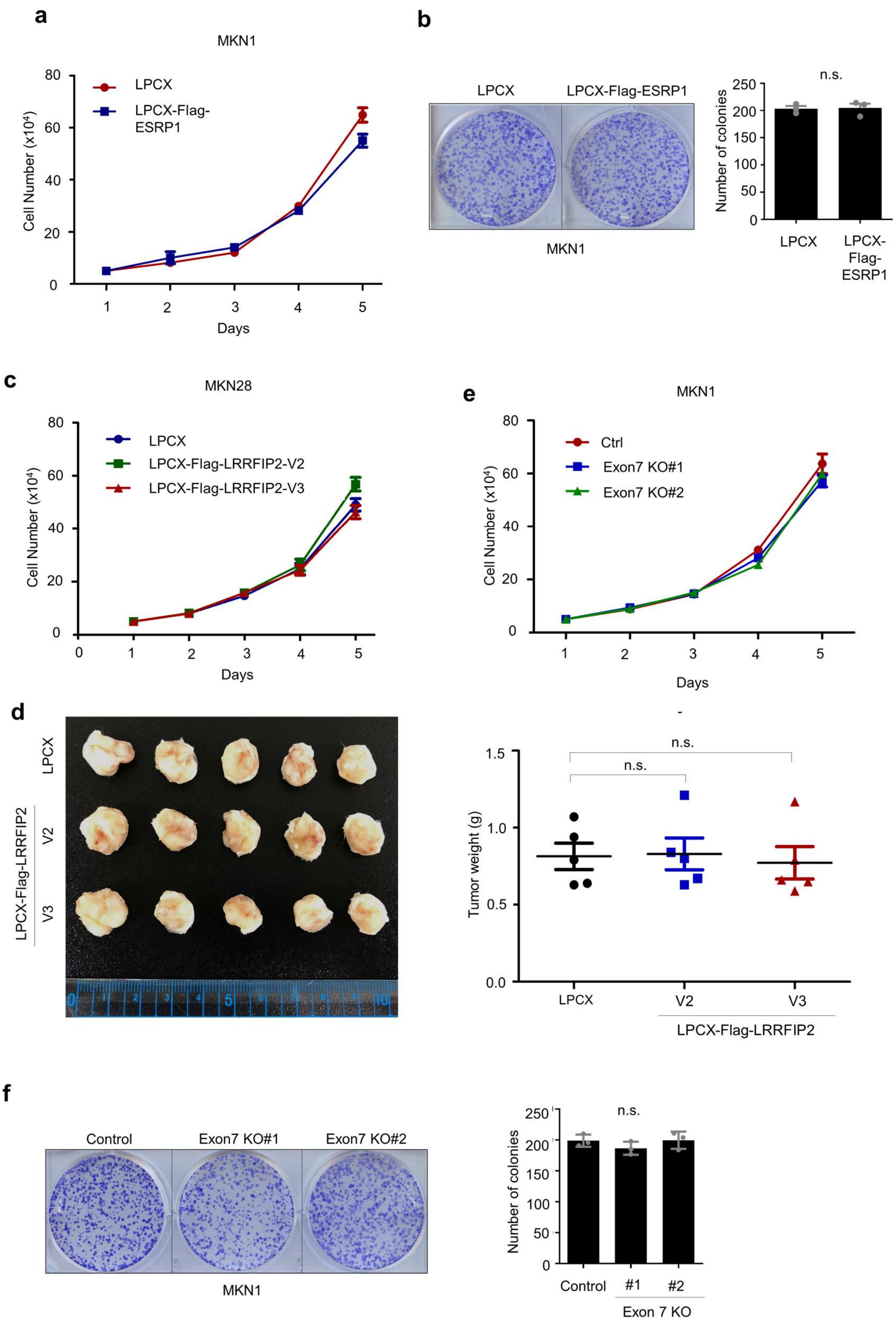

Supplementary Fig. 6. Overexpression of ESRP1 or splicing switch of LRRFIP2 does not alter tumorigenicity of gastric cancer cells. a Cell doubling times of control and ESRP1-overexpressing MKN1 cells. Each point represents the mean of cell numbers counted in triplicate dishes. **b** Foci formation of control and ESRP1-overexpressing MKN1 cells. **c** Cell doubling times of control and LRRFIP2 variants 2 and 3-overexpressing MKN28 cells. **d** Tumor formation and growth of control and LRRFIP2 variants 2 and 3-overexpressing MKN28 cells subcutaneously injected into the flanks of immunodeficient mice (n=6). Representative primary tumor images (left) and tumor weights (right) are shown. **e** Cell doubling times of control and exon 7-deleted MKN1 cells. **f** Foci formation of control and exon 7-deleted MKN1 cells. **a-b, e, f** Data represent the mean ±SD of three independent experiments. **d** Data are representative mean ± SD of five independent animals (n = 5). All *P* values were calculated by unpaired two-tailed Student's t tests. n.s: not significant. Source data are provided in the Source Data file.

Supplementary Fig. 7

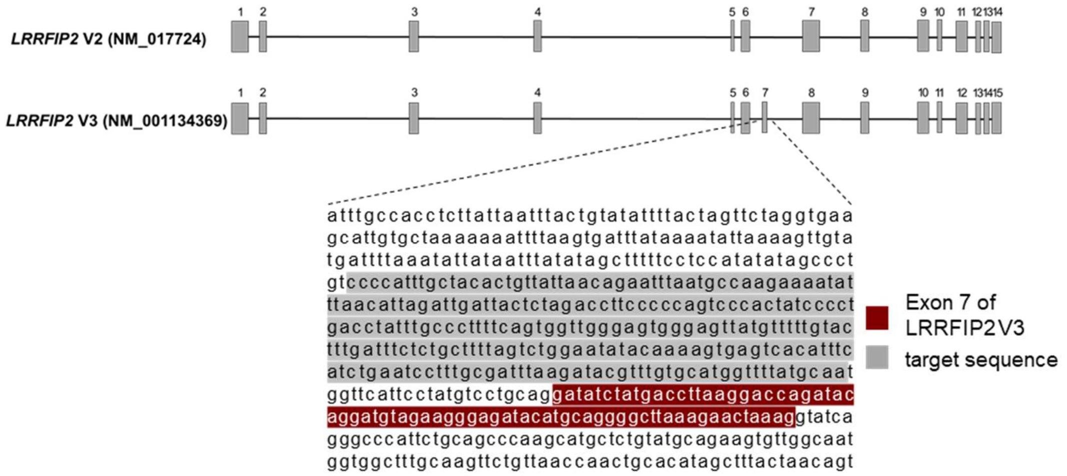

Supplementary Fig. 7. ESRP1 directly binds LRRFIP2 transcript in MKN28 cells. Schematic representation and nucleotide sequence of ESRP1-binding site within LRRFIP2 variant. Red box represents exon 7 of LRRFIP2 V3 and grey box represents the targeted sequence in **Fig. 2c**.

Supplementary Fig. 8

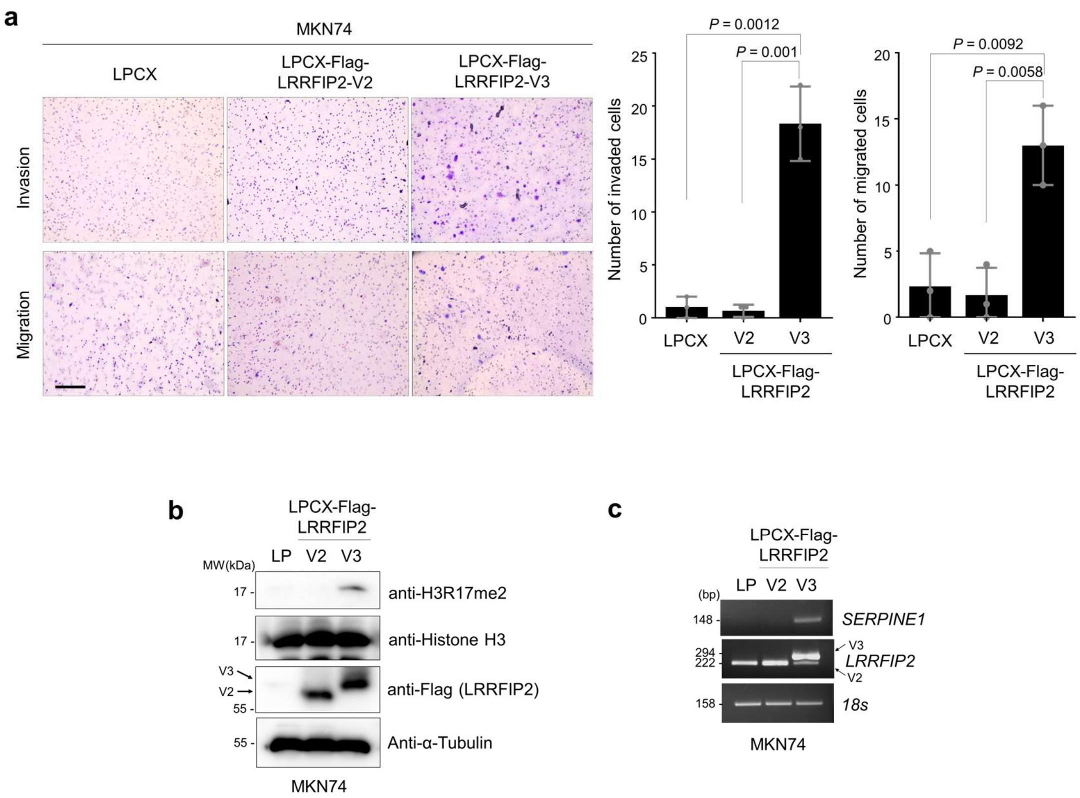

Supplementary Fig. 8. Overexpression of LRRFIP2 variant 3 increases invasiveness and migration of MKN74 cells. a Transwell migration assay and Matrigel invasion assay of LRRFIP2 variants 2 and 3-overexpressing MKN74 cells (left) and bar graphs showing number of invaded and migrated cells (right), respectively, following staining with crystal violet. Original magnification, _40X. Scale bar, 0.5 mm. **b** RT-PCR analysis of *LRRFIP2* and *SERPINE1* in MKN74 cells overexpressing variants 2 and 3. **c** Immunoblot analysis of Flag-tagged LRRFIP2 and histone H3R17 methylation. **a** Data represent the mean ±SD of three independent experiments. **b**,**c** The representative results were obtained from at least two independent experiments. All *P* values were calculated by unpaired two-tailed Student's t tests. These data represent the mean ± S.D Source data are provided in the Source Data file.

Supplementary Fig. 9

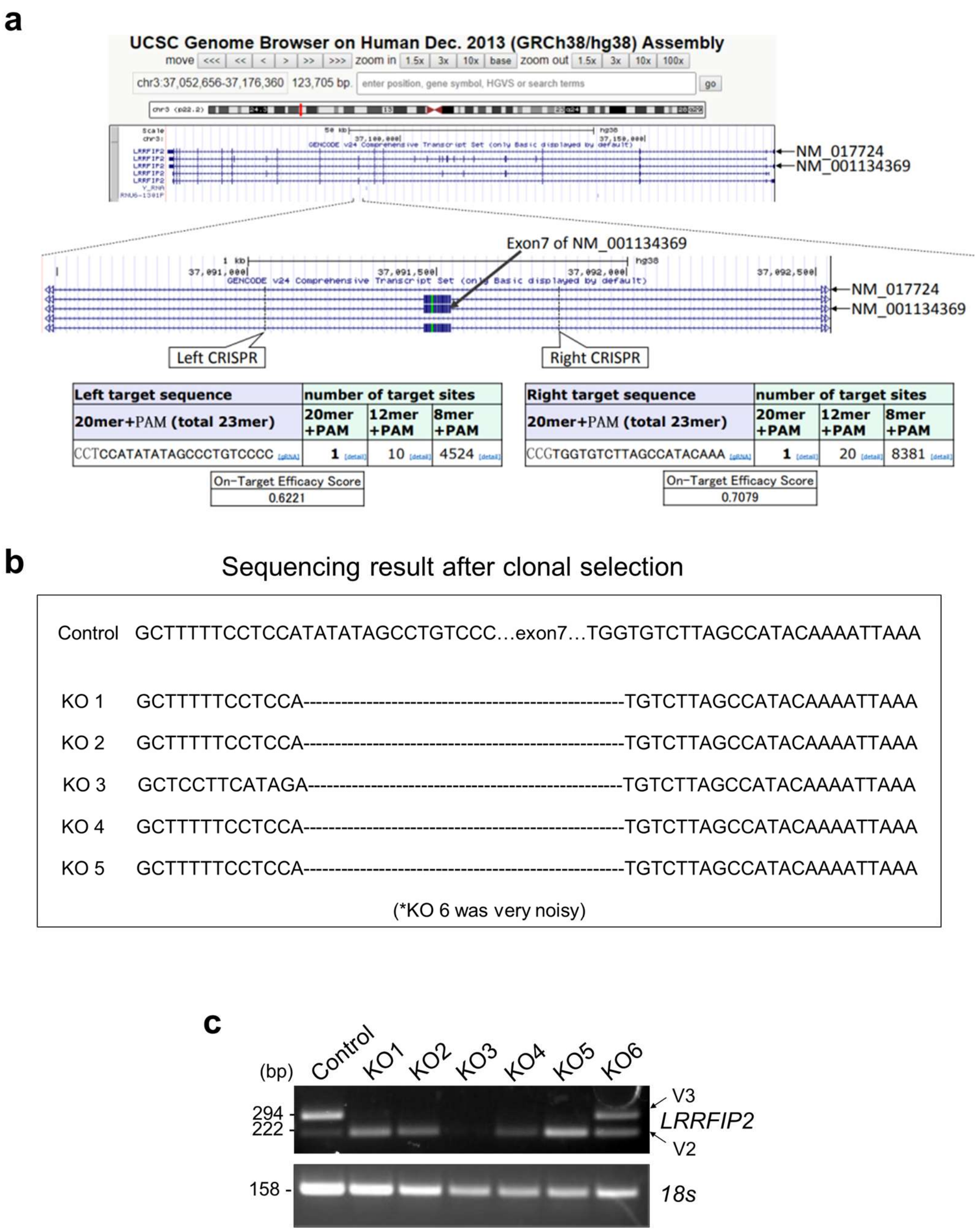

Supplementary Fig. 9. Knockout cell lines were generated using CRISPR/Cas9 system. **a** Two target sites of CRISPR/Cas9 designed to excise exon7. **b** Sequencing result of the clones after clonal selection. **c** RT-PCR analysis of LRRFIP2 in the knockout clones. 2% agarose gel was used to separate the bands of variants (294bp and 222bp).

Supplementary Fig. 10

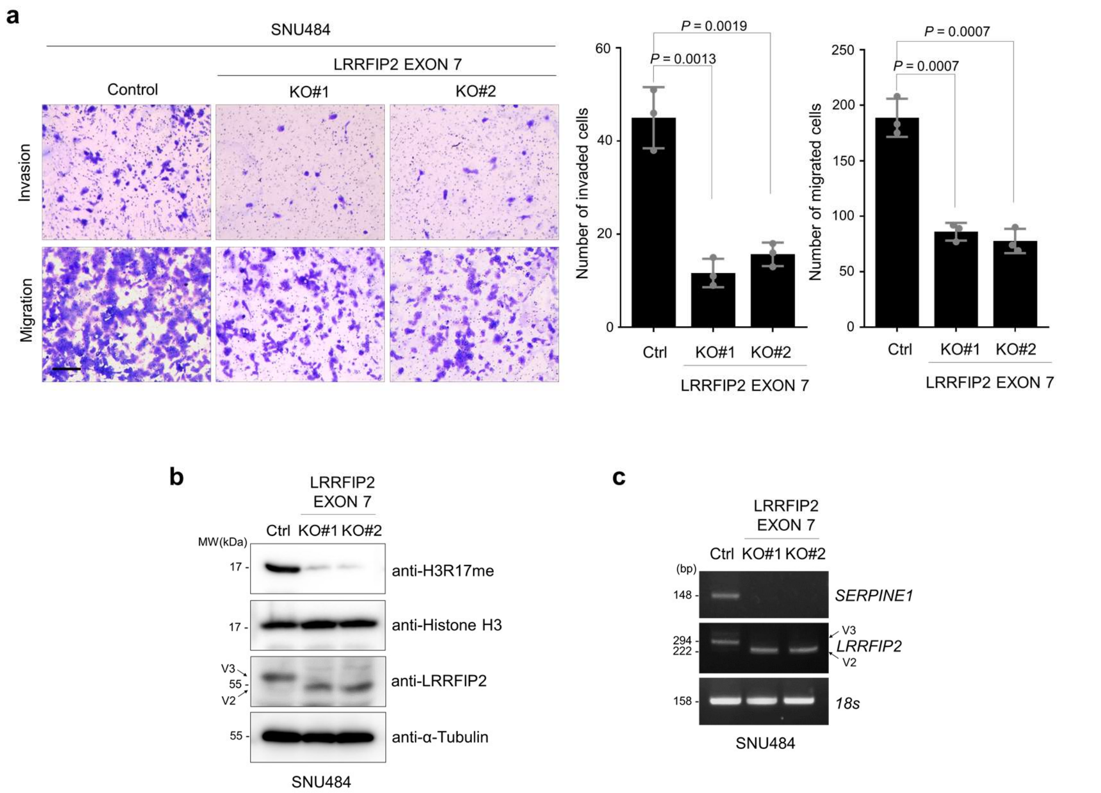

Supplementary Fig. 10. Knockout of LRRFIP2 exon 7 reduces metastatic potential of SNU484 cells. a Transwell migration assay and Matrigel invasion assay of exon 7-deleted cell lines (left) and bar graphs showing number of invaded and migrated cells (right), respectively, following staining with crystal violet. Original magnification, _40X. Scale bar, 0.5 mm. **b** RT-PCR analysis of *LRRFIP2* and *SERPINE1* in exon 7-deleted SNU484 cells. **c** Immunoblot analysis of LRRFIP2 and histone H3R17 methylation in exon 7-deleted SNU484 cells. **a** Data are representative mean ± SD of three independent experiments (*N* = 3). **b**,**c** The representative results were obtained from at least two independent experiments. All P values were calculated by unpaired two-tailed Student's t tests. Source data are provided in the Source Data file.

Supplementary Fig. 11

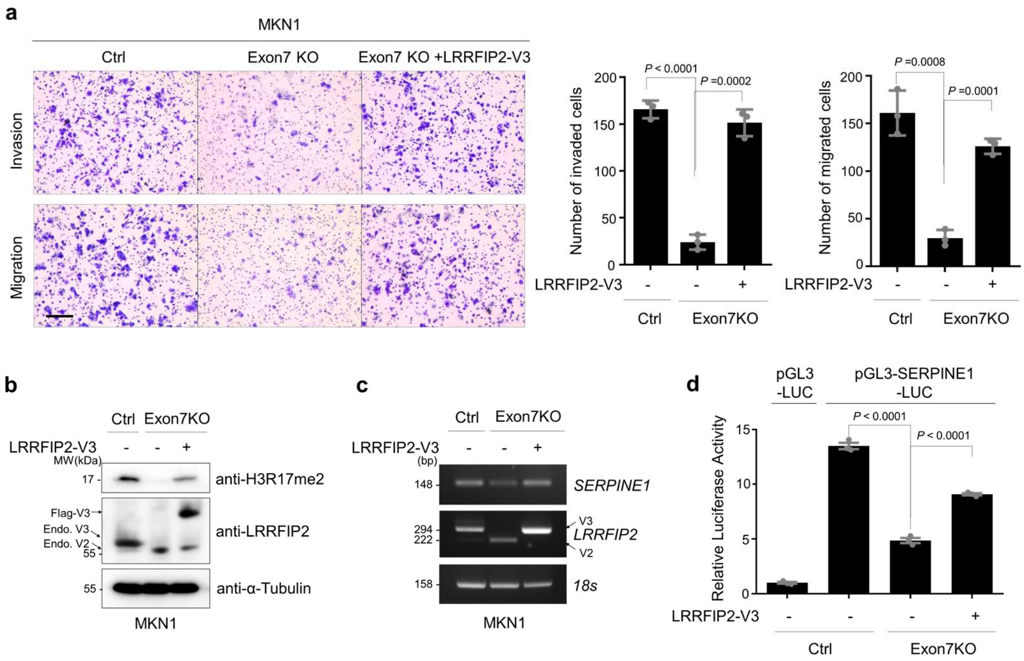

Supplementary Fig. 11. Overexpression of LRRFIP2 variant in exon 7 KO cell line rescues invasiveness and migration of gastric cancer cells. a Transwell migration assay and Matrigel invasion assay of the control, exon 7-knockout, and variant 3-rescued cell lines (left) and bar graphs showing number of invaded and migrated cells (right), respectively, following staining with crystal violet. Original magnification, _40X. Scale bar, 0.5 mm. **b** Immunoblot analysis of histone H3R17 methylation and LRRFIP2. **c** RT-PCR analysis of *LRRFIP2* and *SERPINE1* in MKN1 control and exon 7 knockout cells. **d** Luciferase activity of *SERPINE1* promoter following overexpression of LRRFIP2 V3. **a** Data are representative mean ± SD of three independent experiments (N = 3). **b**,**c** The representative results were obtained from at least two independent experiments. **d** Data are representative mean ± SD of three independent experiments, each conducted in triplicate (n = 3). Unpaired two-tailed Student's t test ($P < 0.05$) with Benjamini-Hochberg correction for multiple tests was conducted for all statistical analyses. These data represent the mean ± S.D. Source data are provided in the Source Data file.

Supplementary Fig. 12

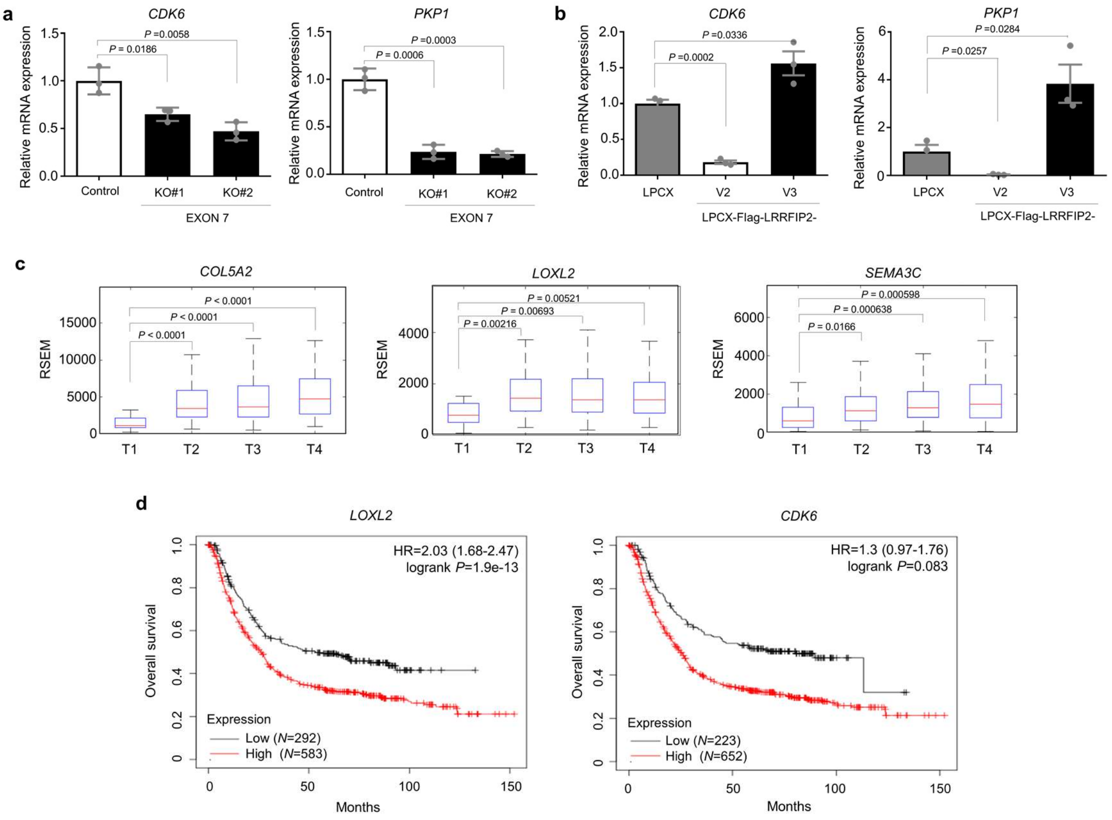

Supplementary Fig. 12. Alternative splicing of LRRFIP2 regulates gene expression in gastric cancer cells. a and **b** Real-time qRT-PCR showing the expression of altered target genes in **a** exon7-deleted MKN1 cells and **b** control, LRRFIP2 variants 2 and 3-overexpressing MKN28 cells. **c** Box plots showing *COL5A2*, *LOXL2* and *SEMA3C* expression levels in each tumor stage in gastric cancer tissues from public TCGA data sets. T1-T4 refers to the size and or/extent of the main tumor into nearby tissues. The center line is the median; the box is from the 25th to the 75th percentile. The upper or lower whisker extends from the hinge to the 1.5 x IQR (distance between the first and third quartiles) from the hinge for up and low, respectively. **d** Kaplan-Meier analysis showing overall survival depending on *LOXL2* and *CDK6* expression levels from public meta-analysis data (*N*=875). **a**, **b** Data are representative mean ± SD of three independent experiments (N = 3). *P* values were calculated by unpaired two-tailed Student's t tests.**c**, **d** *P* values were calculated using a log-rank test. Source data are provided in the Source Data file.

Supplementary Fig. 13

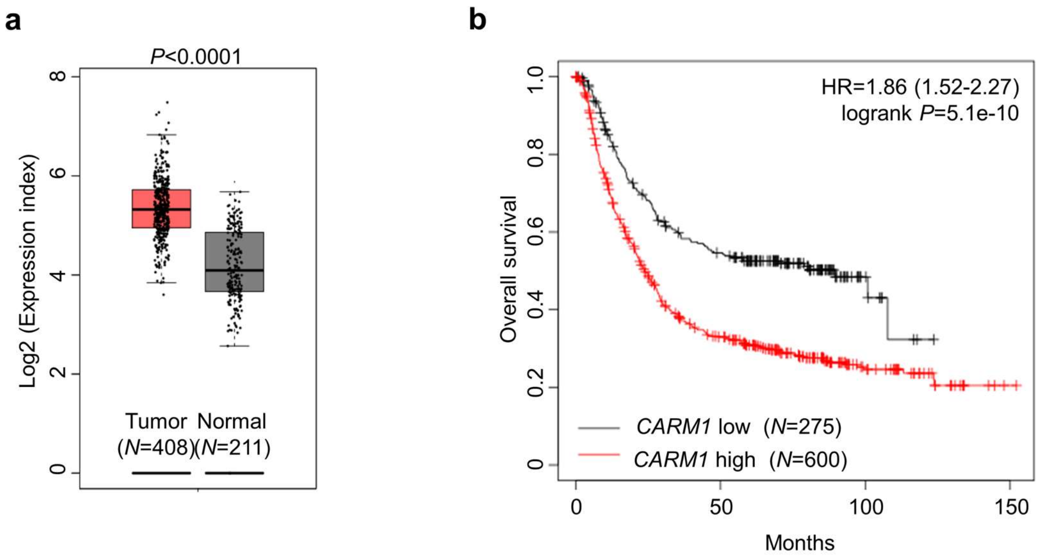

Supplementary Fig. 13. CARM1 is highly expressed in tumor tissues and gastric cancer patients with short relapse-free survival times. a Gene expression analysis of CARM1 using GEPIA based on the TCGA and GTEx database. Box plots represent the gene expression level in terms of log 2 (TPM+1) in the tumor (red, *N* = 408) and normal (grey, *N* = 211) samples, respectively. Normal tissues are matched TCGA adjacent tissue and GTEx data. The center line is the median; the box is from the 25th to the 75th percentile. The upper or lower whisker extends from the hinge to the 1.5 x IQR (distance between the first and third quartiles) from the hinge for up and low, respectively. **b** Kaplan-Meier analysis showing overall survival depending on CARM1 expression levels from public meta-analysis data (*N*=875). All *P* values were calculated using a log-rank test.

Supplementary Fig. 14

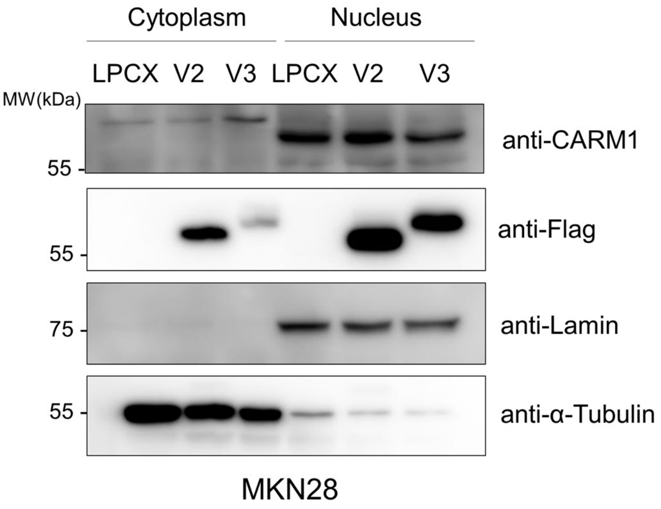

Supplementary Fig. 14. CARM1 and LRRFIP2 variants are co-localized in the nucleus while LRRFIP2 variants are also detected in the cytoplasm. The fractionated cell lysates were immunoblotted to show intracellular localization of CARM1 and LRRFIP2. The representative results were obtained from at least two independent experiments. Source data are provided in the Source Data file.

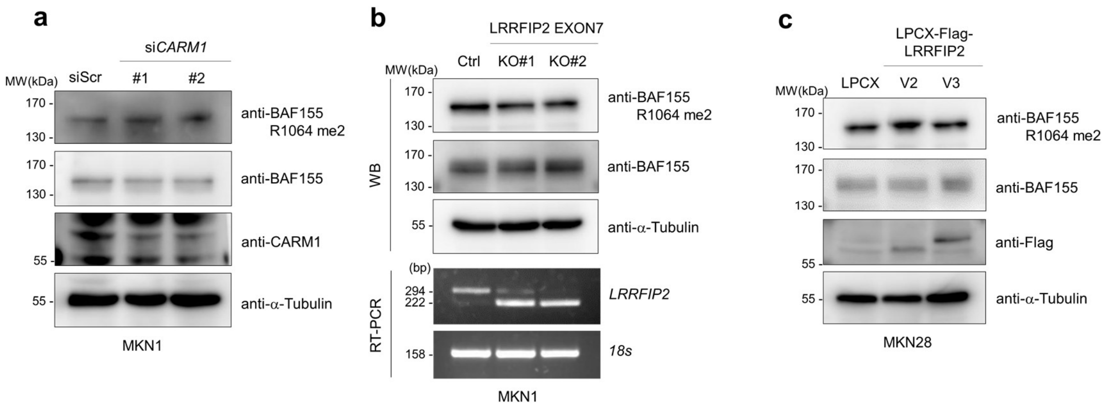

Supplementary Fig. 15. Dimethylation of BAF155 (R1064) is not associated with the expression levels or CARM1 or splicing variants of LRRFIP2 in gastric cancer cells. a Immunoblot analysis of methylation of BAF155 (R1064) and total BAF155 expressions in control and CARM1 siRNA-transfected MKN1 cells. **b** Immunoblot and analysis of methylation of BAF155 (R1064) and total BAF155 expressions and RT-PCR analysis of *LRRFIP2* variants in control and CARM1 siRNA-transfected MKN1 cells. **c** Immunoblot analysis of methylation of BAF155 (R1064) and total BAF155 expressions in control and LRRFIP2 variants 2 and 3-overexpressing MKN28 cells. The representative results were obtained from at least two independent experiments. Source data are provided in the Source Data file.

Supplementary Fig. 16

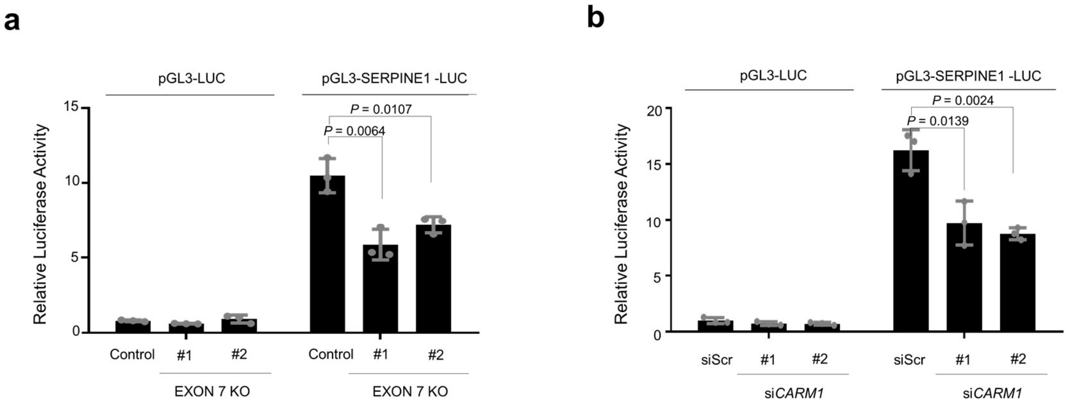

Supplementary Fig. 16. Exon 7 deletion reduces the luciferase activity of the *SERPINE1* promoter. a Control and exon 7-deleted MKN1 cells were transfected with *SERPINE1* promoter (-1500/+500) and then assayed for luciferase activity. **b** Control and CARM1-knockdown MKN1 cells were transfected with *SERPINE1* promoter (-1500/+500) and then assayed for luciferase activity. Data are representative mean ± SD of three independent experiments, each conducted in triplicate (n = 3). Unpaired two-tailed Student's t test ($P < 0.05$) with Benjamini-Hochberg correction for multiple tests was conducted for all statistical analyses. Source data are provided in the Source Data file.

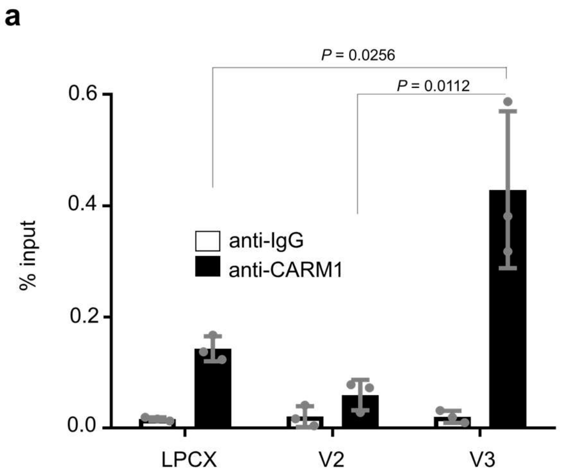

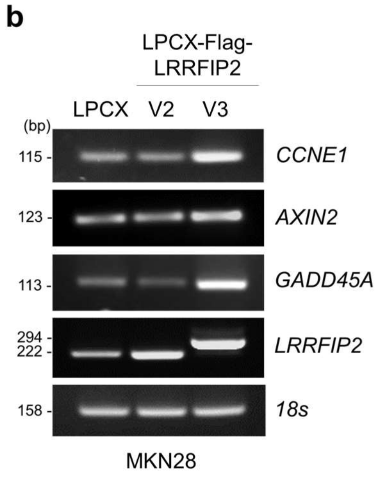

Supplementary Fig. 17. Overexpression of LRRFIP2 variant 3 induces transcription of the target genes of CARM1. a qChIP analysis showing the recruitment of CARM1 to the human *Cyclin E1* promoter (-1546bp/-1331bp) in control and LRRFIP2 variants 2 and 3-overexpressing MKN28 cells. Precipitation was conducted with anti-normal IgG or anti-CARM1 antibodies. **b** RT-PCR analysis of CARM1 target genes (*CCNE1, AXIN2*, and *GADD45A*) and *LRRFIP2* in control and LRRFIP2 variants 2 and 3-overexpressing MKN28 cells. **a** Data represent the mean ±SD of three independent samples. **b** The representative results were obtained from at least two independent experiments. All P values were calculated by unpaired two-tailed Student's t tests. Source data are provided in the Source Data file.

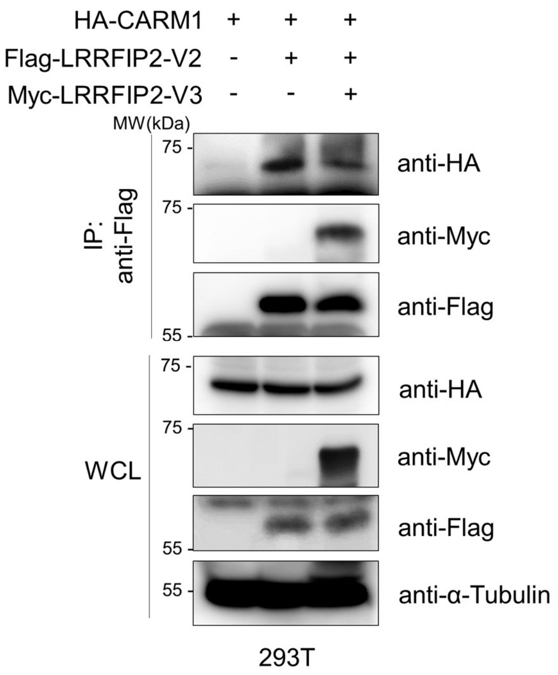

Supplementary Fig. 18. Overexpression of LRRFIP2 variant 3 reduces interaction between CARM1 and LRRFIP2 variant 2. Immunoprecipitation assay showing the interaction between CARM1 and LRRFIP2 variant 2 in the presence of LRRFIP2 variant 3. The representative results were obtained from at least two independent experiments. Source data are provided in the Source Data file.

Supplementary Fig. 19

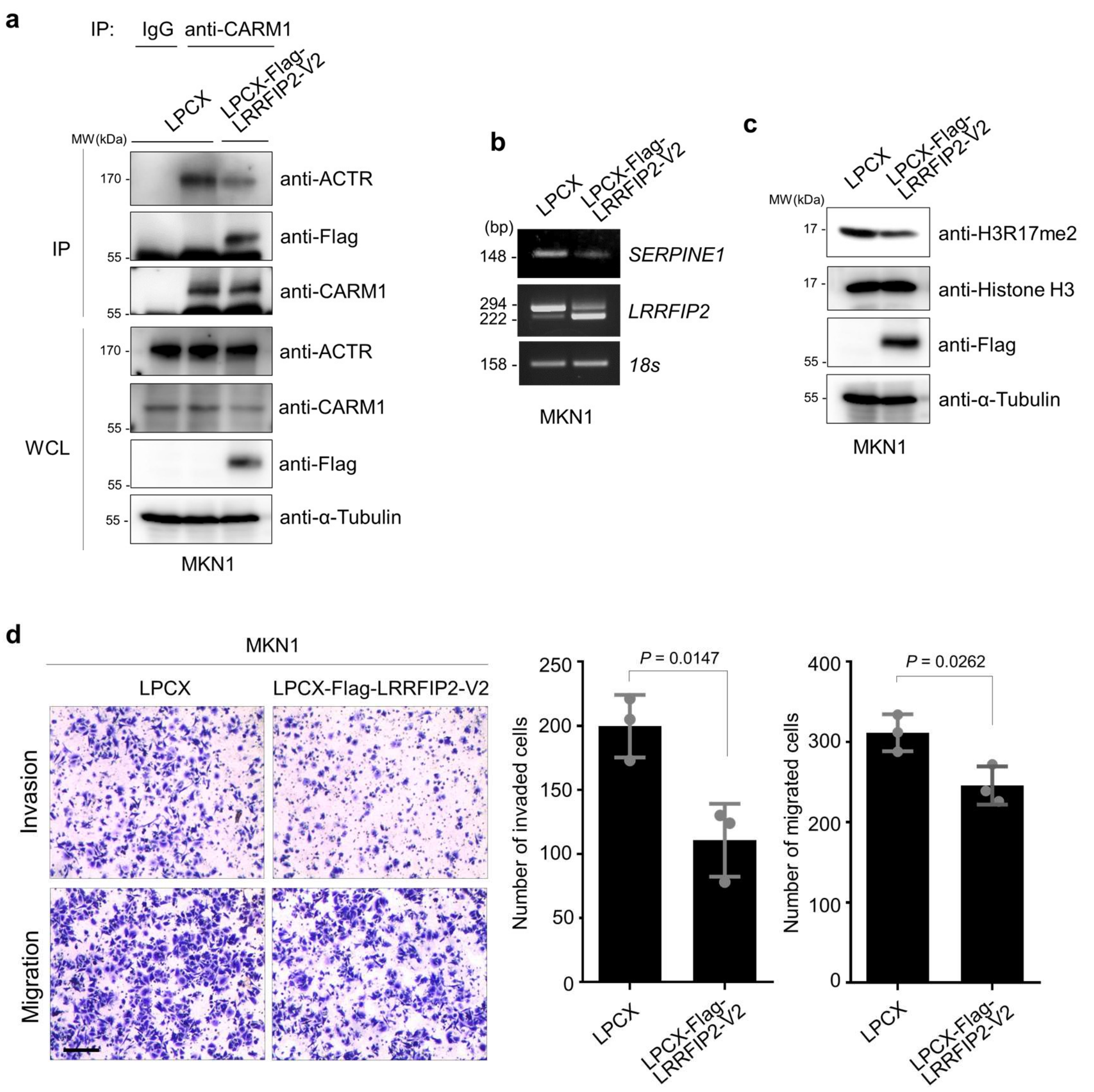

Supplementary Fig. 19. Overexpression of LRRFIP2 variant 2 in MKN1 cells reduces the metastatic potential of gastric cancer cells. a Immunoprecipitation assay showing the interaction between endogenous CARM1 and ACTR. **b** RT-PCR analysis showing mRNA level of *SERPINE1*. **c** Immunoblot analysis of Histone H3R17me2. **d** Transwell migration assay and Matrigel invasion assay of MKN28 cell lines upon overexpression of LRRFIP2 variant 2 (left) and bar graphs showing number of invaded and migrated cells (right), respectively, following staining with crystal violet. **a-c** The representative results were obtained from at least two independent experiments. **d** Data represent the mean ±SD of three independent experiments. Original magnification, _40X. Scale bar, 0.5 mm. All *P* values were calculated by unpaired two-tailed Student’s t tests. Source data are provided in the Source Data file.

Supplementary Fig. 20

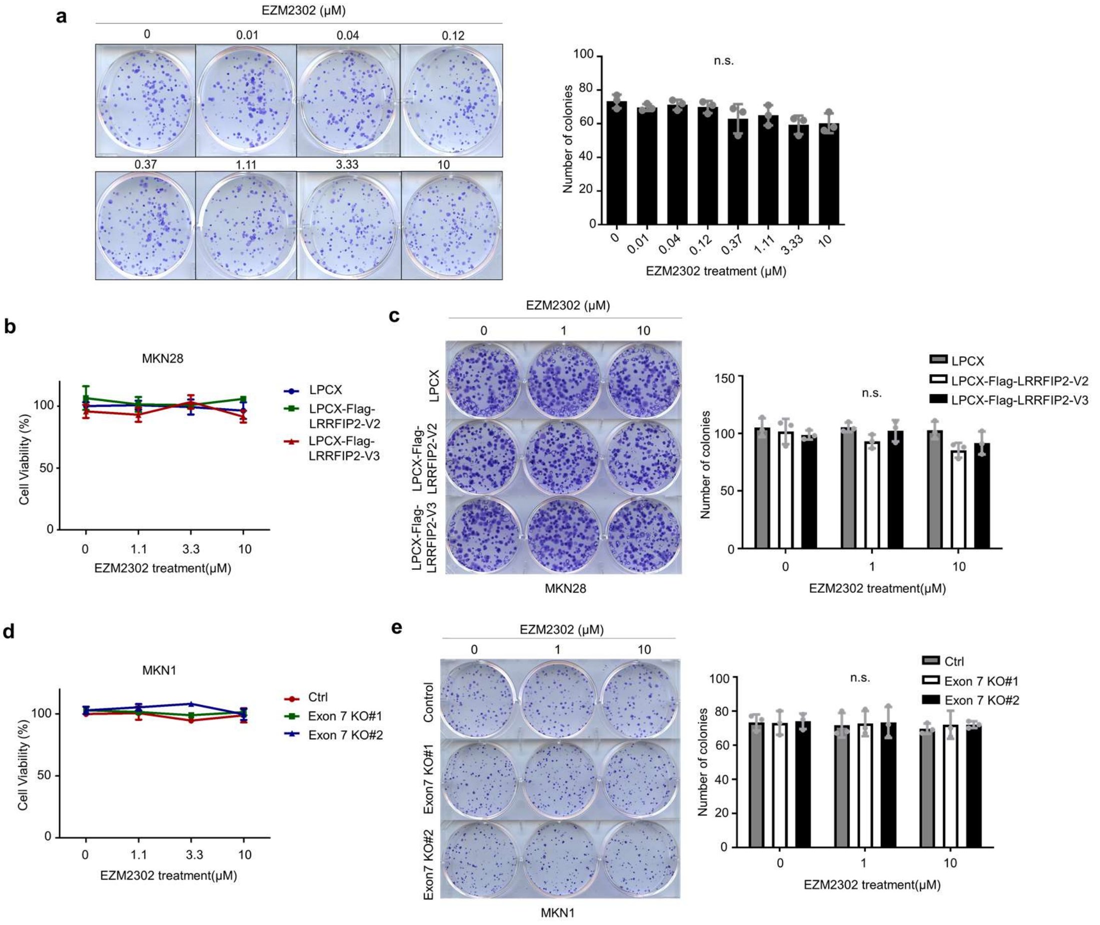

Supplementary Fig. 20. EZM2302 treatment does not affect cell proliferation of MKN1 and MKN28 cells *in vitro*. a Colony formation assays performed in cells with EZM2302 treatment. **b** Cell doublings of MKN28 cells overexpressing LRRFIP2 variants 2 and 3 with EZM treatment. **c** Colony formation assays performed in MKN28 cells overexpressing LRRFIP2 variants 2 and 3 with EZM2302 treatment. **d** Cell doublings of control and exon 7 deleted MKN1 cells with EZM2302 treatment. **e** Colony formation assay performed in control and exon 7 deleted MKN1 cells with EZM treatment. Data represent the mean ±SD of three independent experiments. All P values were calculated by unpaired two-tailed Student's t tests. n.s: not significant. Source data are provided in the Source Data file.

Supplementary Fig. 21 Unprocessed original scans of blots and gels

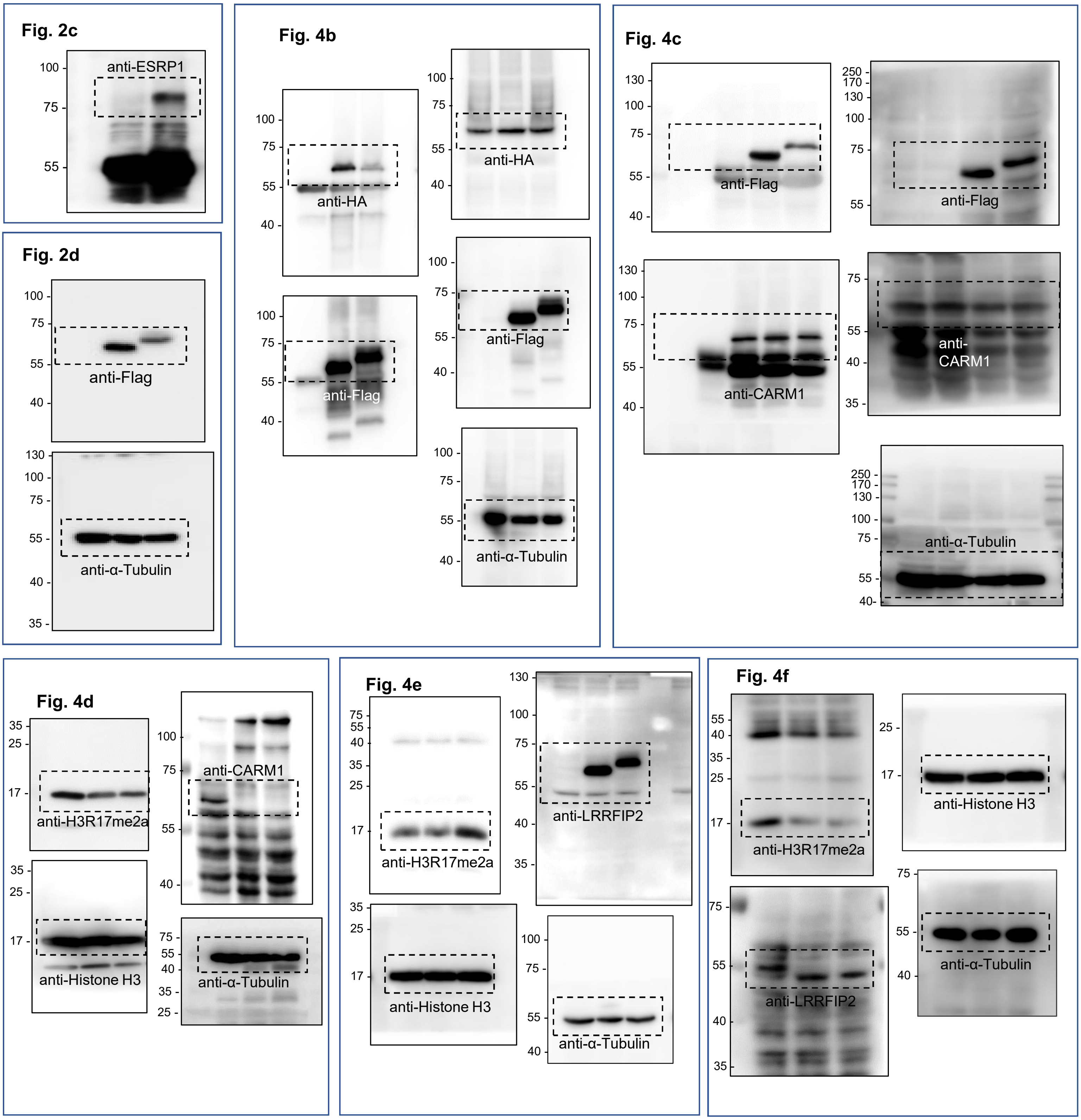

Fig. 5a

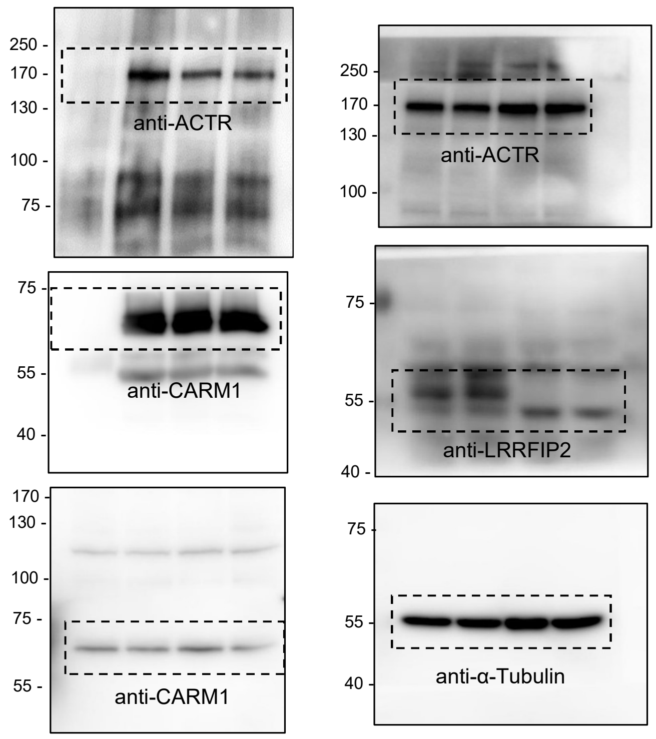

Fig. 5b

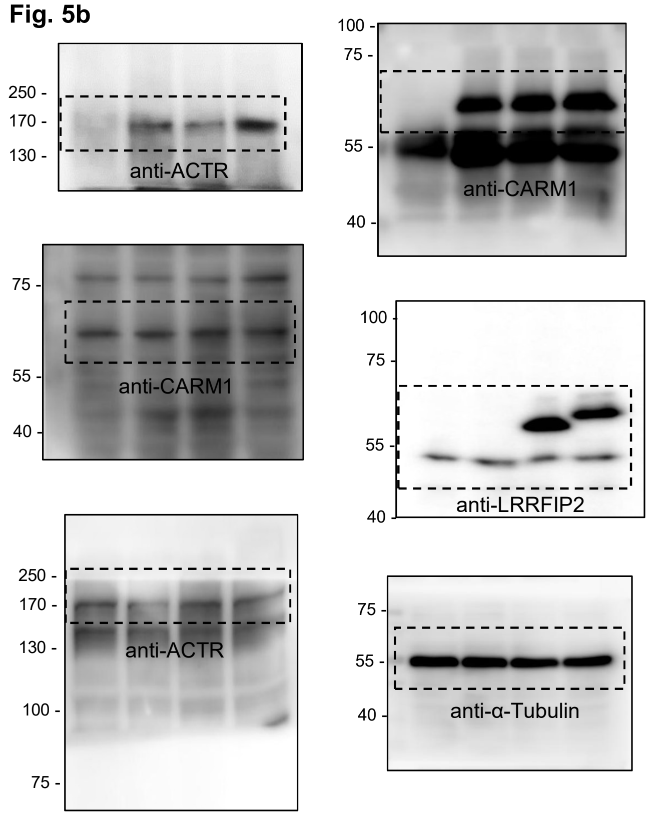

Fig. 5c

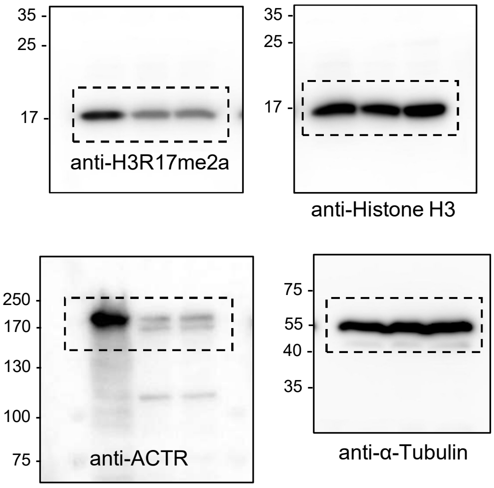

Fig. 6a

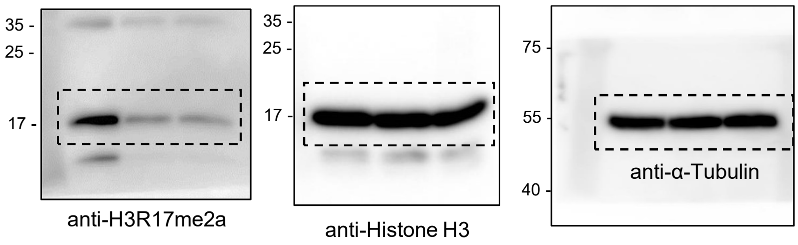

Fig. 6b

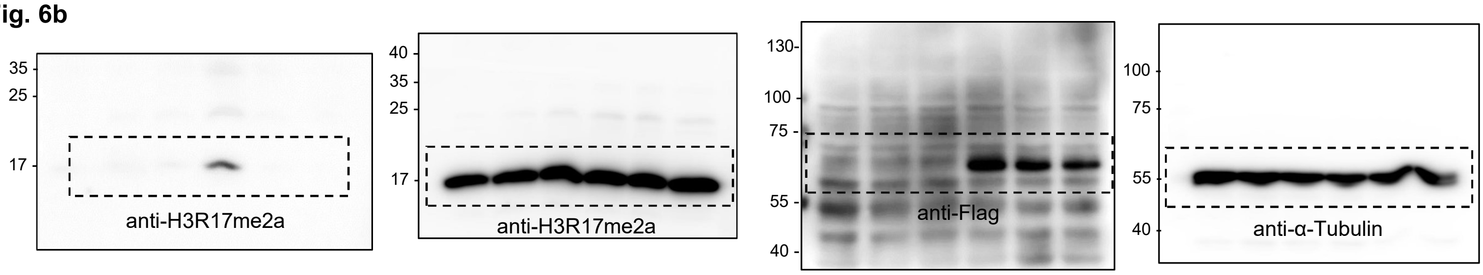

Supplementary Fig. 21 Unprocessed original scans of blots and gels -Continued

Fig. S2

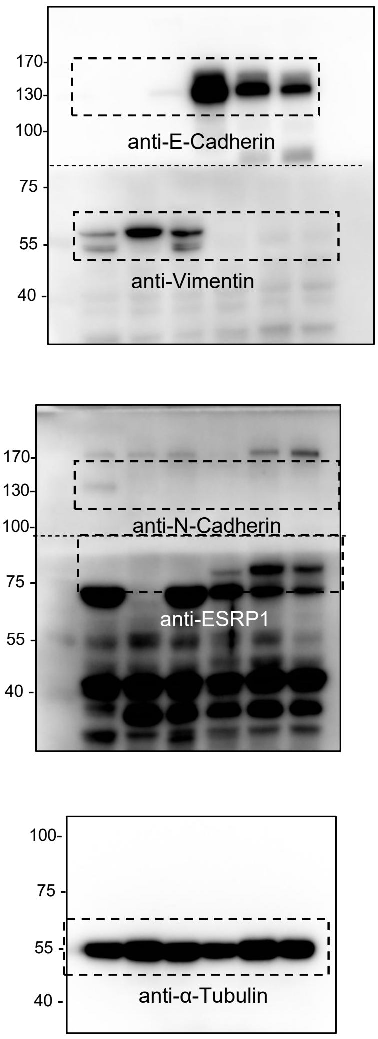

Fig. S8

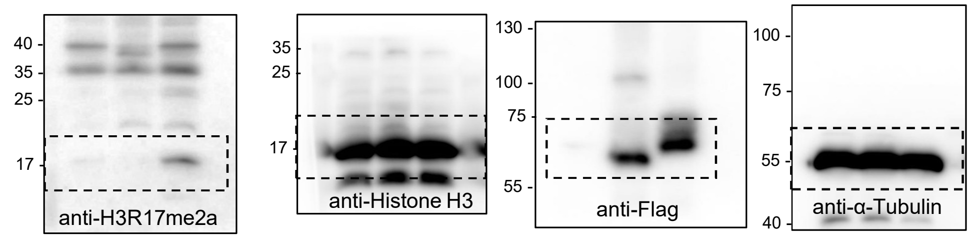

Fig. S10

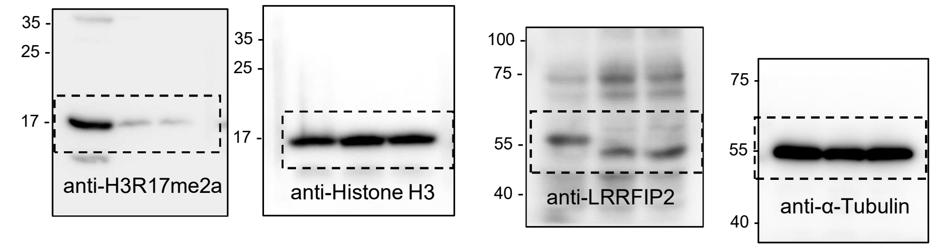

Fig. S11

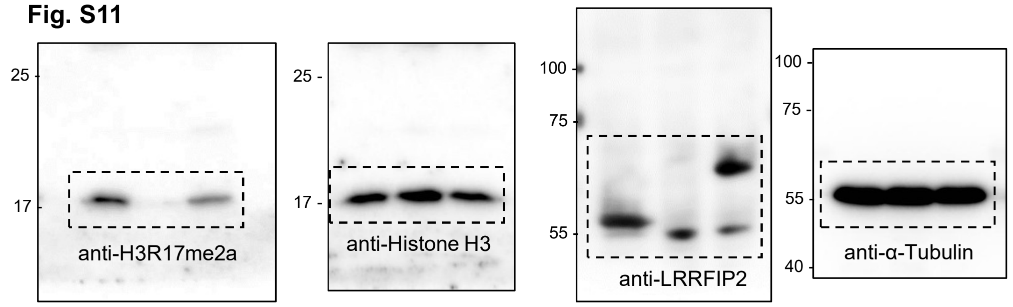

Fig. S14

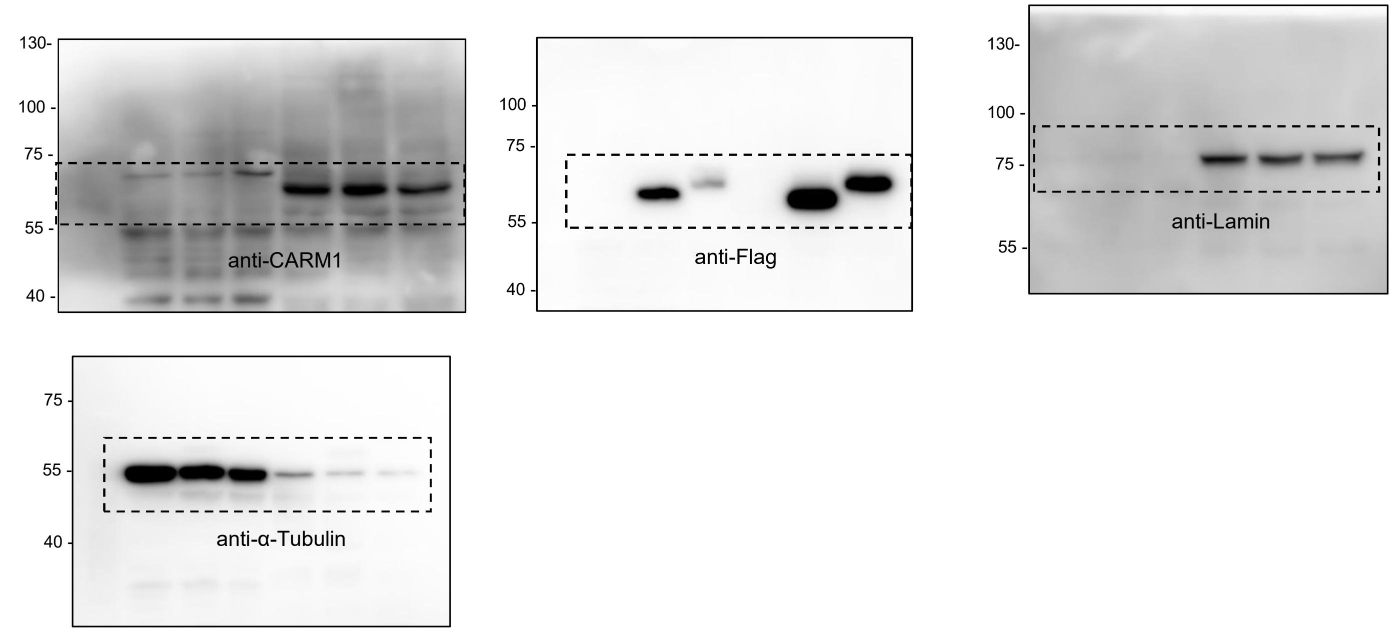

Supplementary Fig. 21 Unprocessed original scans of blots and gels -Continued

Fig.S15a

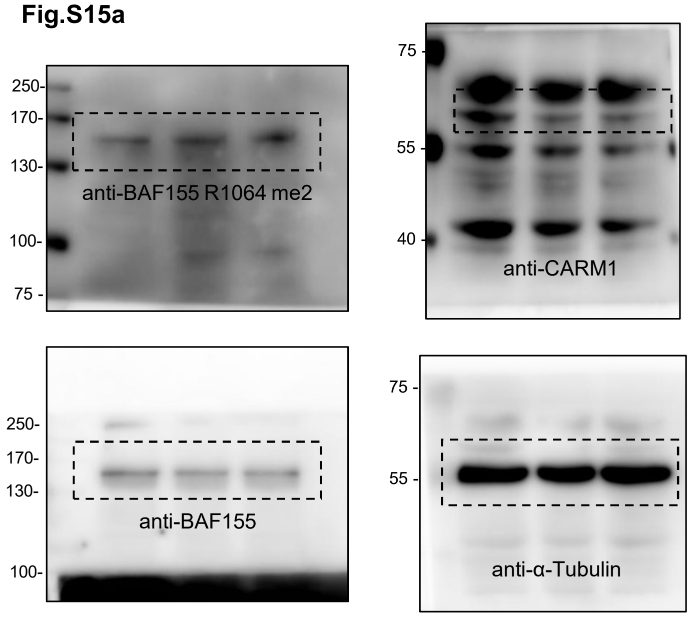

Fig.S15b

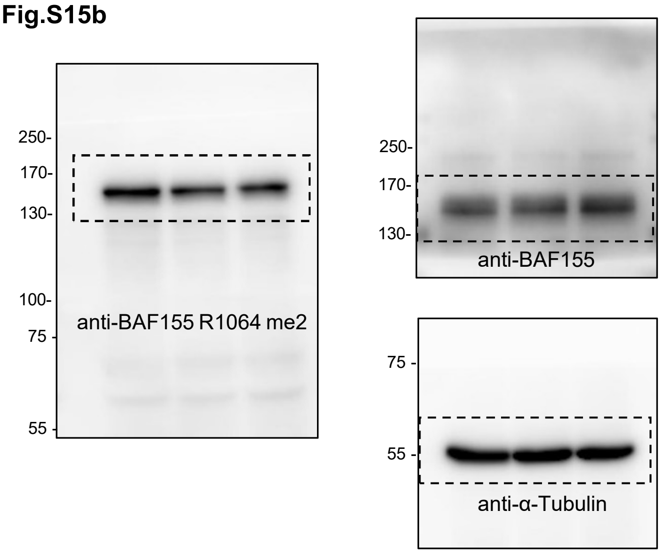

Fig.S15c

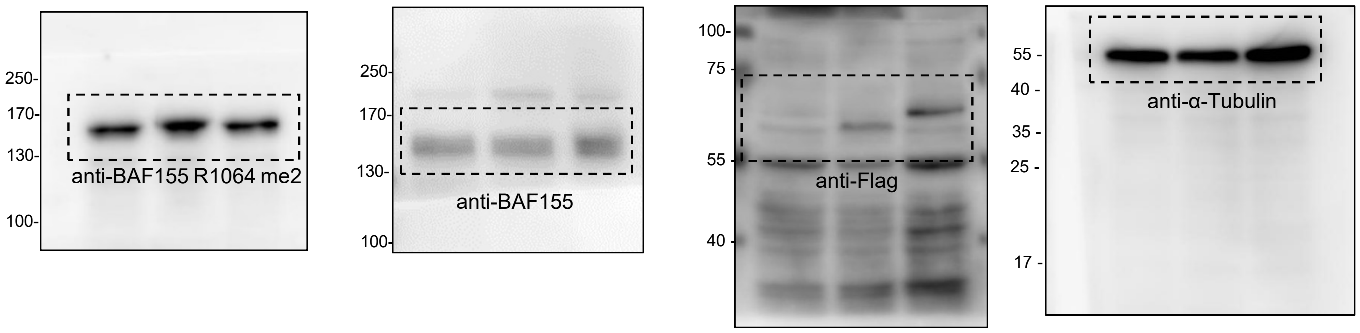

Fig.S18

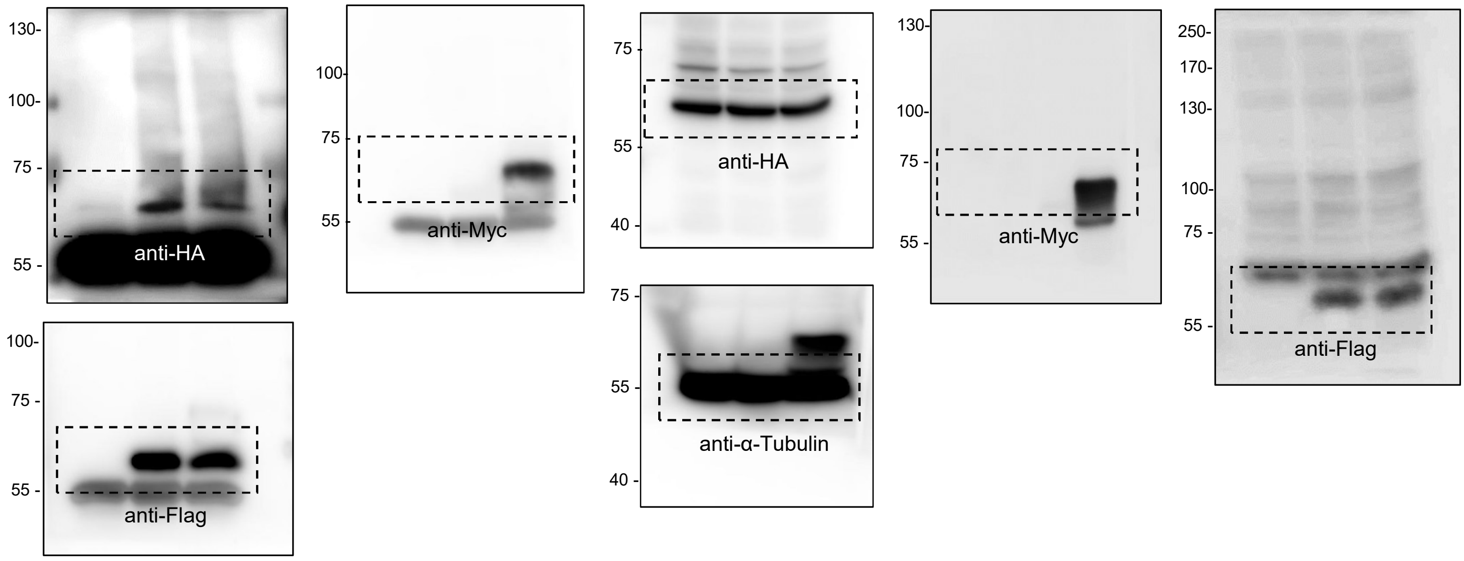

Supplementary Fig. 21 Unprocessed original scans of blots and gels -Continued

Fig.S19a

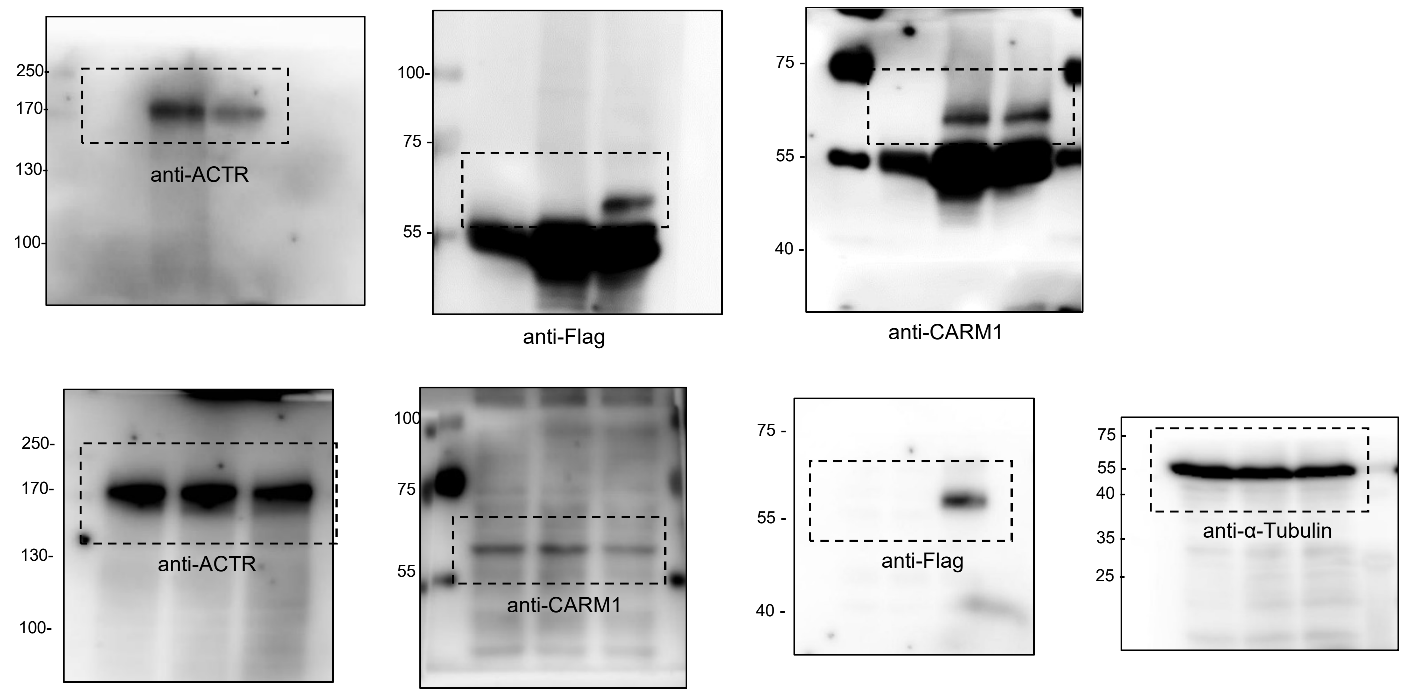

Fig.S19c

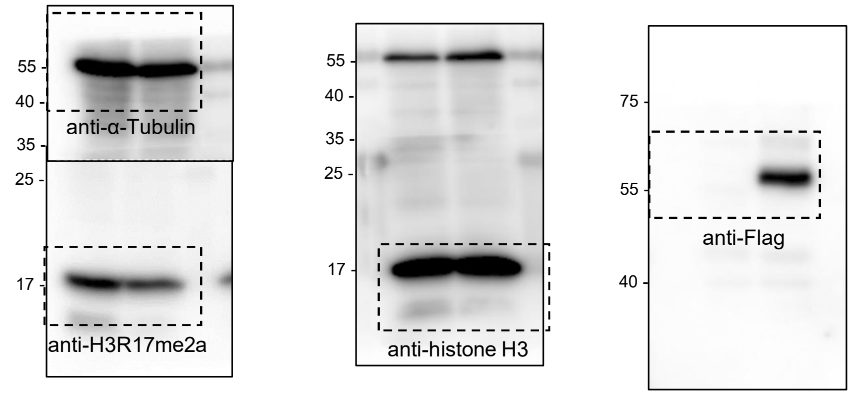

Supplementary Fig. 21 Unprocessed original scans of blots and gels -Continued

Fig. 1j

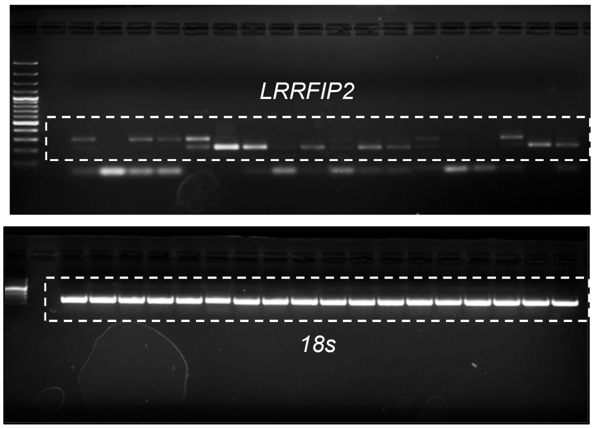

Fig. 2b

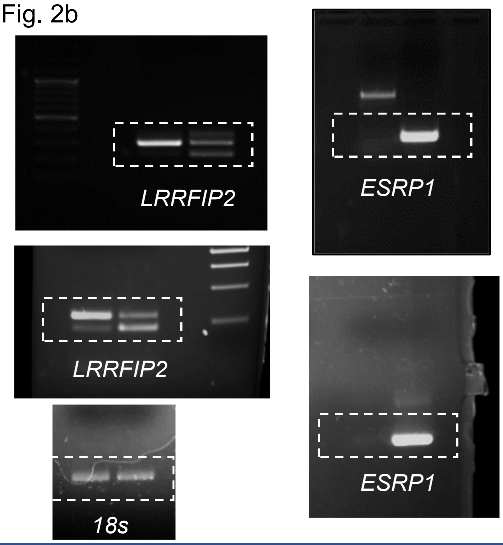

Fig. 2c

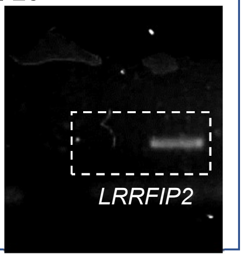

Fig. 2d

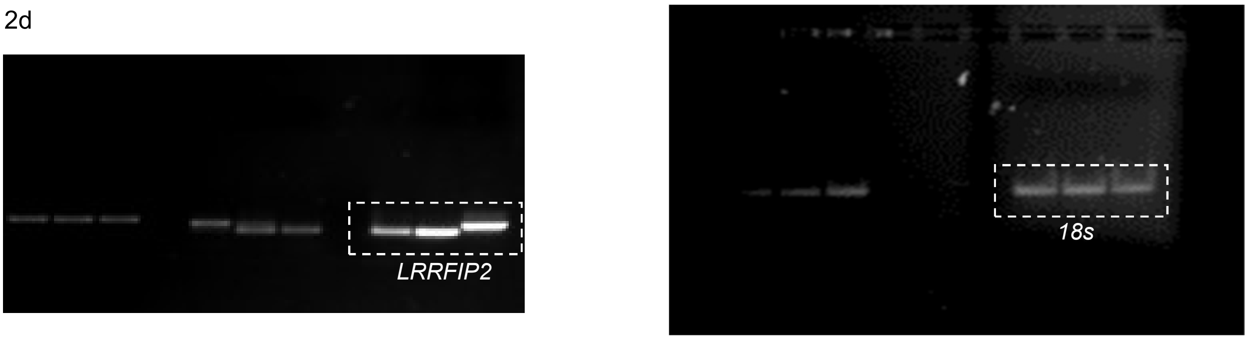

Fig. 2i

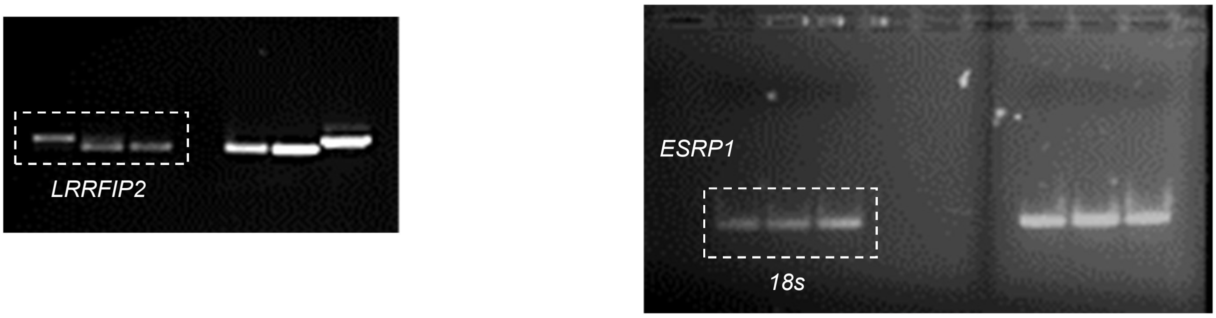

Fig. 4f

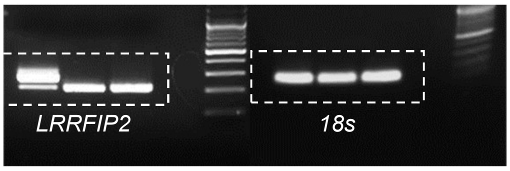

Fig. 4g

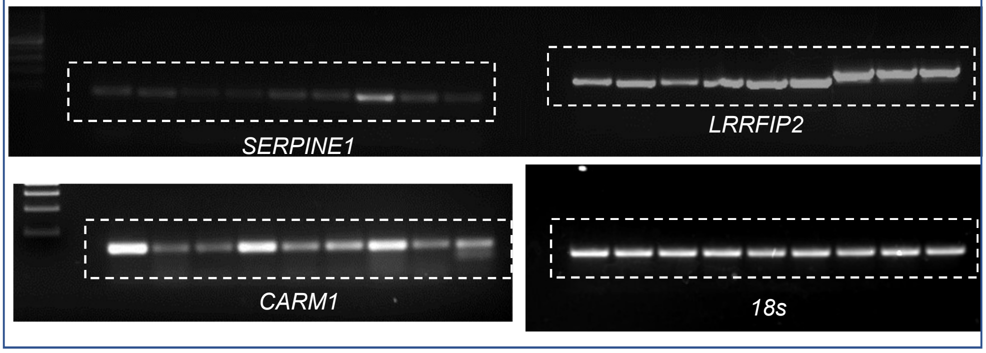

Fig. 6f

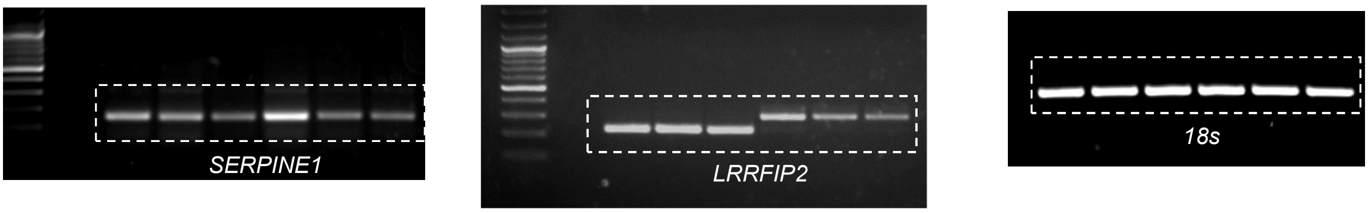

Supplementary Fig. 21 Unprocessed original scans of blots and gels -Continued

Fig. S3

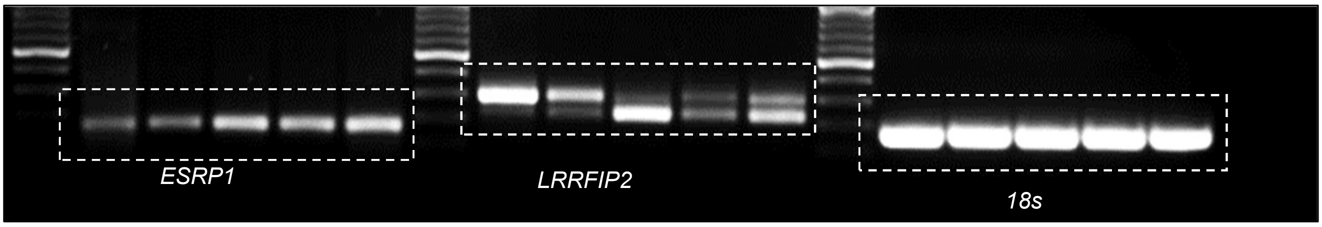

Fig. S8b

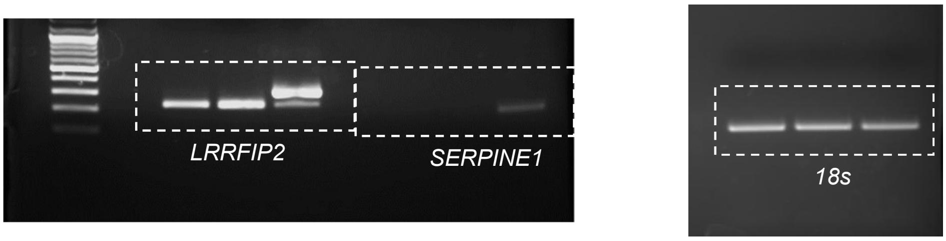

Fig. S9b

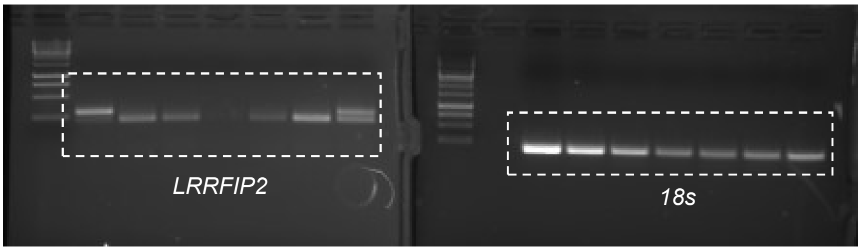

Fig. S10b

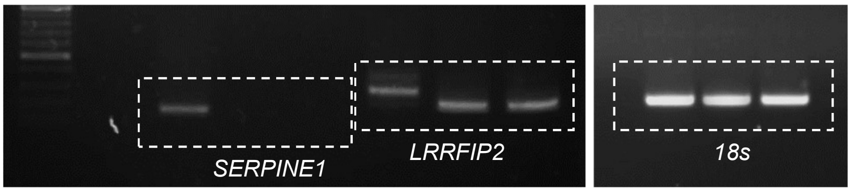

Fig. S11c

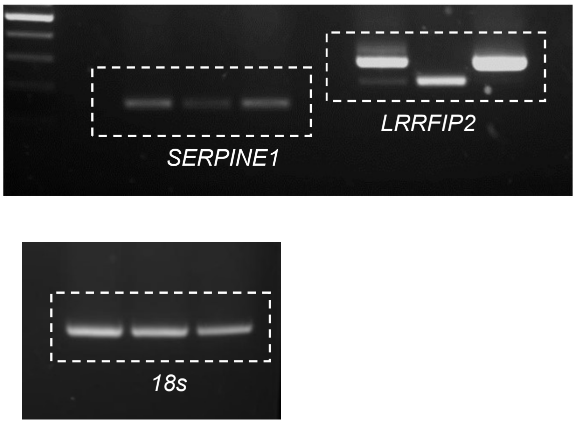

Fig. S17b

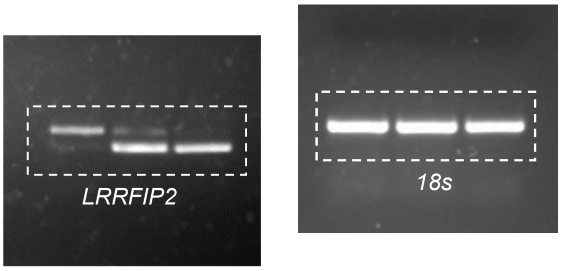

Fig. 17b

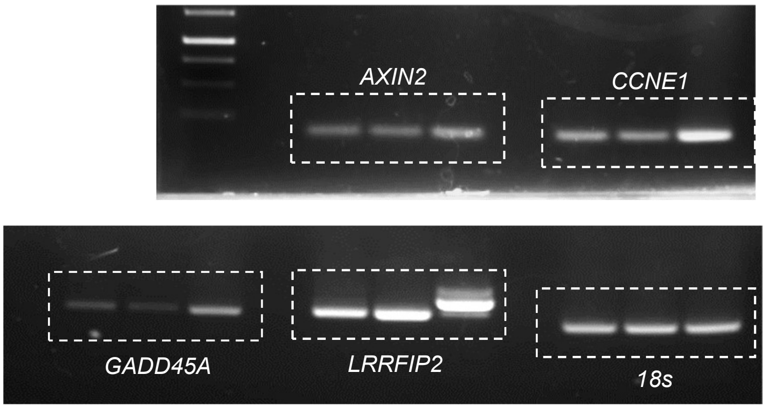

Fig. 19b

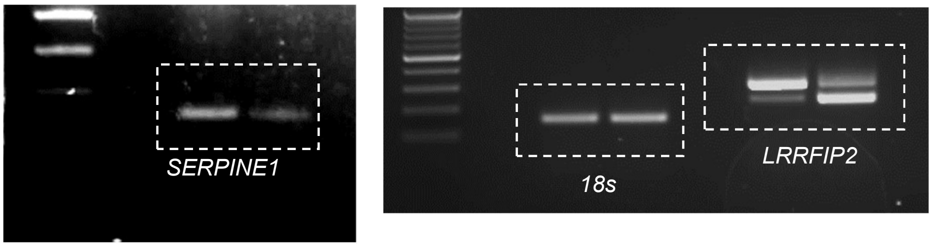

Supplementary Table 1. A list of primer sequences used.

	Construct	Species	Direction	Sequence (5' - 3')
Cloning	pCS4-3Flag-LRRFIP2	Human	Forward	GATC CTCGAG GGGA CTCCTGCTTC TGGA
			Reverse	GATC GCTAGC CTA CTGCTGGGCCAGAAGT
	pCS4-3Flag-ESRP1	Human	Forward	ATGC AGGCCT ACGG CCTCTCCGGATTAC
			Reverse	ATGC TCTAGA TTAAATACAAACCCATTCT
	pCS4-3HA-CARM1	Human	Forward	ATGAATTCAATGGCAGCGGCGGCGGCGGCGGTG
			Reverse	GATCTAGACTAGCTCCCGTAGTGCATGGTGTTGG
RT-PCR	LRRFIP2	Human	Forward	CCTCAGCAACAACCCCTCTA
			Reverse	CCTGCTCTTCAATAACATCC
	ESRP1	Human	Forward	ACGGAGGACTGCAAAGAAGA
			Reverse	CTGACATGAAGCTGCCCATC
	SERPINE1	Human	Forward	TCTGCCCTCACCAACATTCT
			Reverse	CGGTCATTCCCAGGTTCTCT
	CARM1	Human	Forward	GGCTCCAAGTCCAGTAACCT
			Reverse	TCCACATGTTTTCCGAGGGA
	CCNE1	Human	Forward	AAGTGGCGTTTAAGTCCCCT
			Reverse	GATTTGCTGGGGATACTGCG
	GADD45A	Human	Forward	CTGGAGAGCAGAAGACCGAA
			Reverse	CTGGATCAGGGTGAAGTGGA
	AXIN2	Human	Forward	TGACCCTGGGCCACTTTAAA
			Reverse	CCGTCTCATCCTCCCAGATC
	18S	Human	Forward	CCCAACTTCTTAGAGGGACA
			Reverse	TAGTCAAGTTCGACCGTCTT
qRT-PCR	SERPINE1	Human	Forward	TCTGCCCTCACCAACATTCT
			Reverse	CGGTCATTCCCAGGTTCTCT
	CARM1	Human	Forward	GGCTCCAAGTCCAGTAACCT
			Reverse	TCCACATGTTTTCCGAGGGA
	COL5A2	Human	Forward	TGAGAAGGGAATGGCTGGAG
			Reverse	AATTCCTCTTTCTCCCGGCA
	LOXL2	Human	Forward	TGCAGCGACAAAAGGATTCC
			Reverse	CTTGCGGTAGGTTGAGAGGA
	SEMA3C	Human	Forward	TTCAACCCCAACGTGAACAC
			Reverse	TCAGTTCTGACCGCATTCCT
	CD74	Human	Forward	GCTGTCGGGAAGATCAGAAG
			Reverse	CAGGATGGAAAAGCCTGTGT
	PKP1	Human	Forward	AGTCCAAGTCTTCCCAGTCG
			Reverse	TATCCCCATGAGCCATTCCC
	CDK6	Human	Forward	TGCACAGTGTCACGAACAGA
			Reverse	ACCTCGGAGAAGCTGAAACA
	ACTR	Human	Forward	TCGAGTGTCTAGTCCTCCCA
			Reverse	AAACTGCTGCTGGGAAAAGG
	18S	Human	Forward	AATGCTTCTCTGGCACGTCT
			Reverse	TCTTCCATCTCACGCATCTG
ChIP-PCR	qChIP SERPINE1 (+336/+474)	Human	Forward	ACCCCATCACCCAGTAACAA
			Reverse	ACAGTGCCCAGCCTTAATTC
	qChIP CCNE1 (-1626bp/-1441bp)	Human	Forward	AGACCCTGGCTCTAACTTCA
			Reverse	ACCGGCTAATGAGAGAGACA

Supplementary Table 2. A list of Primary Antibodies used

Antibody	Catalog number	Dilution	Species Reactivity	Supplier
LRRFIP2	ab172367	Immunoblotting (1:1000)	Mouse	Abcam
PRMT4/CARM1 (3H2)	#12495S lot: 2	Immunoblotting (1:1000)	Mouse	Cell Signaling
		Immunoprecipitation (1:200)		
SRC1 (128E7)	#2191S lot: 2	Immunoblotting (1:1000)	Rabbit	Cell Signaling
SRC-2 (D2X4M)	#96687S lot: 1	Immunoblotting (1:1000)	Rabbit	Cell Signaling
SRC-3 (5E11)	#2126S lot:4	Immunoblotting (1:1000)	Rabbit	Cell Signaling
ESRP1	21045-1AP	Immunoblotting (1:1000)	Rabbit	Proteintech
		RNA-Immunoprecipitation (1:200)		
Asymmetric Dimethyl- SMARCC1/BAF155 (Arg1064) (D8I3U)	#94962S lot: 1	Immunoblotting (1:1000)	Rabbit	Cell Signaling
SMARCC1/BAF155 (D7F8S)	#11956S lot: 4	Immunoblotting (1:1000)	Rabbit	Cell Signaling
Histone H3 (asymmetric di methyl R17)	ab8284	Immunoblotting (1:1000)	Rabbit	Abcam
		Chromatin Immunoprecipitation (1:100)		
Histone H3	ab1791	Immunoblotting (1:1000)	Rabbit	Abcam
Vimentin (V9)	ab8069	Immunoblotting (1:1000)	Rabbit	Abcam
N-Cadherin	610920	Immunoblotting (1:1000)	Mouse	BD Biosciences
E-Cadherin	610181	Immunoblotting (1:1000)	Mouse	BD Biosciences
PAI-1 (C-9)	SC-5297 lot: K1 308	Immunohistochemistry (1:50)	Mouse	Santa Cruz
Myc (9E10)	sc-40	Immunoblotting (1:1000)	Mouse	Santa Cruz
HA (F-7)	sc-7392	Immunoblotting (1:1000)	Mouse	Santa Cruz
Flag (M2)	F3165	Immunoblotting (1:5000)	Mouse	Sigma
		Immunoprecipitation (1:1000)		
α-tubulin	T5168	Immunoblotting (1:5000)	Mouse	Sigma
Normal mouse IgG	sc-2025	Immunoprecipitation (1:200)	Mouse	Santa Cruz
normal rabbit IgG	sc-2027	Immunoprecipitation (1:200)	Rabbit	Santa Cruz

Supplementary Table 3. A list of Secondary Antibodies used

Antibody	Catalog number	Dilution	Supplier
Goat Anti-Mouse IgG Antibody, Peroxidase Conjugated	AP124P	Immunoblotting (1:5000)	Millipore
Goat Anti-Rabbit IgG Antibody, Peroxidase Conjugated	AP132P	Immunoblotting (1:5000)	Millipore

Supplementary Table 4. A list of 100 isoform switches found in ESRP1-low versus ESRP-high cell lines

Gene		Correlation in tissue samples			Correlation in cell lines		
isoform 1	isoform 2	correlation (isoform1) - correlation (isoform2)	correlation between isoform1 and ESRP1	correlation between isoform2 and ESRP1	correlation (isoform1) - correlation (isoform2)	correlation between isoform1 and ESRP1 (cell line)	correlation between isoform2 and ESRP1 (cell line)
LRRFIP2,NM_017724	LRRFIP2,NM_001134369	1.381752178	0.688443474	-0.693308704	0.97379806	0.497312109	-0.476485951
CCDC50,NM_178335	CCDC50,NM_174908	1.31241368	0.65620684	-0.65620684	1.406426626	0.703213313	-0.703213313
CD47,NM_001777	CD47,NM_198793	1.29059511	0.645297555	-0.645297555	0.915481284	0.457740642	-0.457740642
BICD2,NM_001003800	BICD2,NM_015250	1.265403536	0.632701768	-0.632701768	1.046785009	0.523392504	-0.523392504
HRH1,NM_001098211	HRH1,NM_001098212	1.164102405	0.660633845	-0.50346856	0.231277771	0.173664847	-0.057612924
PLEKHA1,NM_001001974	PLEKHA1,NM_001195608	1.125816513	0.709199383	-0.41661713	0.080604319	-0.475125094	-0.555729413
ATL1,NM_001127713	ATL1,NM_181598	1.119446089	0.557190701	-0.562255388	0.570742853	0.285705498	-0.285037355
MYH14,NM_001077186	MYH14,NM_024729	1.083237132	0.558239648	-0.524997483	1.159997053	0.602228286	-0.557768767
MYOF,NM_133337	MYOF,NM_013451	1.077609438	0.538804719	-0.538804719	1.299430112	0.649715056	-0.649715056
DNM2,NM_001005360	DNM2,NM_001005362	0.990115359	0.279221697	-0.710893661	1.235540909	0.604536625	-0.631004284
GAB1,NM_002039	GAB1,NM_207123	0.98902836	0.49451418	-0.49451418	1.140428091	0.570214046	-0.570214046
CD44,NM_001001389	CD44,NM_001001391	0.938275914	0.44830022	-0.489975694	0.659375411	0.155354777	-0.504020634
MARVELD3,NM_001017967	MARVELD3,NM_001271329	0.918941779	0.427900465	-0.491041314	0.683053341	0.221679828	-0.461373512
VGLL4,NM_014667	VGLL4,NM_001128219	0.869398651	0.444741848	-0.424656803	1.598219406	0.799988203	-0.798231203
ARMCX5:ARMCX5-GPRASP2:GPRASP2,NM_001168482	ARMCX5:ARMCX5-GPRASP2:GPRASP2,NM_001004051	0.843486267	0.597395443	-0.246090824	0.479075777	0.116839362	-0.362236415
PDGFA,NM_002607	PDGFA,NM_033023	0.836493828	0.418246914	-0.418246914	0.980246431	0.490123216	-0.490123216
TACC2,NM_001291876	TACC2,NM_006997	0.834665253	0.555003295	-0.279661958	0.697197186	0.429700534	-0.267496652
GNAI2,NM_001282620	GNAI2,NM_001282618	0.767006191	0.510066261	-0.25693993	0.533419264	0.372088972	-0.161330291
ZNF33A,NM_001278179	ZNF33A,NM_001278171	0.747989479	0.497600802	-0.250388677	0.127577486	0.196507963	0.068930477
NF2,NM_181825	NF2,NM_181829	0.742338389	0.732358229	-0.009980159	0.368851696	0.074493053	-0.294358643
CLDN12,NM_001185073	CLDN12,NM_001185072	0.729467261	0.406240353	-0.323226908	1.247764587	0.573032636	-0.674731951
STX2,NM_194356	STX2,NM_001980	0.675828879	0.337914439	-0.337914439	0.284512399	0.1422562	-0.1422562
SERINC2,NM_001199039	SERINC2,NM_001199038	0.668356918	0.553273596	-0.115083322	0.853048769	0.455292934	-0.397755835
NUMB,NM_001005743	NUMB,NM_001005744	0.659357802	0.338673818	-0.320683984	0.322758787	-0.214672474	-0.537431261
FGFR2,NM_022970	FGFR2,NM_001144918	0.648327015	0.306532115	-0.341794901	0.661580641	0.377373587	-0.284207054
SLAIN1,NM_001242869	SLAIN1,NM_001242870	0.592722499	0.641646541	0.048924042	0.304824393	0.035316431	-0.269507962
SH3YL1,NM_015677	SH3YL1,NM_001282682	0.579141757	0.13070577	-0.448435986	0.9333773	0.496000402	-0.437376898
ERBB2IP,NM_001253699	ERBB2IP,NM_001006600	0.562702039	0.31816139	-0.244540649	0.776015576	0.420849154	-0.355166423
GPR56,NM_001145770	GPR56,NM_001145771	0.54026628	0.223077227	-0.317189053	1.105754357	0.731364509	-0.374389848
ZNF280D,NM_001288588	ZNF280D,NM_001288589	0.519266469	0.489038838	-0.030227631	0.47693259	0.160334497	-0.316598092
MORF4L2,NM_001142426	MORF4L2,NM_001142432	0.50881448	0.182340594	-0.326473886	0.573266804	0.114465713	-0.458801092
EDRF1,NR_110858	EDRF1,NM_015608	0.413307694	0.051676304	-0.36163139	0.677502378	0.144364558	-0.53313782
RAI14,NM_001145520	RAI14,NM_001145522	0.398392409	-0.110944989	-0.509337398	0.580295183	0.117596548	-0.462698635
FGFR3,NM_022965	FGFR3,NM_000142	0.392002542	0.139675844	-0.252326698	0.986793244	0.354525035	-0.632268209
MAST4,NM_001297651	MAST4,NM_015183	0.386556905	0.223155865	-0.16340104	0.240474957	-0.053837915	-0.294312871
SGOL1,NM_001012409	SGOL1,NM_001012413	0.344519726	0.178957533	-0.165562193	0.656838301	0.160816796	-0.496021505
ZNHIT3,NM_001281432	ZNHIT3,NR_104011	0.343772407	0.148524257	-0.195248151	0.429322573	0.08351208	-0.345810493
PAX6,NM_001258462	PAX6,NM_000280	0.340637437	-0.181314127	-0.521951564	0.095395432	0.256410758	0.161015326
TRAK1,NM_001265610	TRAK1,NM_001265608	0.334937827	0.135506945	-0.199430882	0.434323554	-0.022605644	-0.456929198
MUC1,NM_001204293	MUC1,NM_002456	0.325973823	0.066986047	-0.258987776	0.457620041	0.446704993	-0.010915048
BAIAP2,NM_006340	BAIAP2,NM_001144888	0.313617581	-0.353127129	-0.66674471	0.97966345	0.559243108	-0.420420342
ZNF419,NM_001098494	ZNF419,NM_024691	0.2842207	-0.095645732	-0.379866432	0.057374084	0.118253169	0.060879085
PTGES3,NM_001282604	PTGES3,NR_104219	0.27213726	0.179404518	-0.092732742	0.576332206	0.386174829	-0.190157376
HNF1B,NM_000458	HNF1B,NM_001165923	0.248038379	0.12401919	-0.12401919	0.583168929	0.291584465	-0.291584465
FAM107B,NM_001282696	FAM107B,NM_001282700	0.246992276	0.022745305	-0.224246972	1.081294532	0.588554602	-0.49273993
PGAP2,NR_104272	PGAP2,NR_045927	0.243239603	0.092139975	-0.151099627	0.283897984	0.048812826	-0.235085158
CSF1,NM_172211	CSF1,NM_172212	0.209004226	0.226610866	0.01760664	0.896973272	0.512872487	-0.384100785
PHLDB1,NM_001144758	PHLDB1,NM_015157	0.203227285	0.085417447	-0.117809838	0.386429908	-0.069493487	-0.455923395
ANAPC10,NM_001256711	ANAPC10,NM_001256709	0.197061163	0.062768286	-0.134292876	0.50482574	0.071846247	-0.432979493
ZNF180,NM_013256	ZNF180,NM_001278508	0.192263196	0.46443063	0.272167434	0.262506022	-0.089458301	-0.351964324
BCL2L11,NM_001204106	BCL2L11,NM_001204111	0.161608971	-0.02355055	-0.185159521	0.4164055	0.172985061	-0.243420439
GSN,NM_001258030	GSN,NM_001258029	0.15425994	-0.206503523	-0.360763463	0.379230254	0.009914304	-0.36931595
CTNND1:TMX2:TMX2-CTNND1,NM_001085463	CTNND1:TMX2:TMX2-CTNND1,NM_001206889	0.14559481	-0.287559045	-0.433153856	1.100917996	0.614690151	-0.486227846
SLC44A3,NM_152369	SLC44A3,NM_001258342	0.142742336	0.025671046	-0.117071289	0.348329067	-0.111643252	-0.459972319

Supplementary Table 4. A list of 100 isoform switches found in ESRP1-low versus ESRP-high cell lines-**Continued**

Gene		Correlation in tissue samples			Correlation in cell lines		
isoform 1	isoform 2	correlation (isoform1) - correlation (isoform2)	correlation between isoform1 and ESRP1	correlation between isoform2 and ESRP1	correlation (isoform1) - correlation (isoform2)	correlation between isoform1 and ESRP1 (cell line)	correlation between isoform2 and ESRP1 (cell line)
SYTL2,NM_001162953	SYTL2,NM_032943	0.118023882	0.611557921	0.493534039	0.841960542	0.511177868	-0.330782674
NFATC2,NM_001258294	NFATC2,NM_001258295	0.118002002	-0.413217025	-0.531219027	0.795169081	0.406546273	-0.388622808
CLIP4,NM_001287527	CLIP4,NM_001287528	0.114427503	-0.109589435	-0.224016938	0.349369229	0.215455109	-0.13391412
ARHGAP8:PRR5:PRR5-ARHGAP8,NM_015366	ARHGAP8:PRR5:PRR5-ARHGAP8,NM_001017528	0.106488007	0.162544274	0.056056267	0.755064754	0.473704156	-0.281360598
FAM86B3P,NR_024362	FAM86B3P,NR_024363	0.099321256	0.632918085	0.533596829	0.850099957	0.371689368	-0.47841059
MYB,NM_001130172	MYB,NM_001130173	0.081797403	0.02501324	-0.056784163	0.859839182	0.520512986	-0.339326196
MIER1,NM_001077702	MIER1,NM_001146111	0.066707574	-0.195339887	-0.262047462	0.566734178	-0.037453525	-0.604187703
POLL,NR_033406	POLL,NM_001174085	0.037896954	-0.098539793	-0.136436747	0.836594715	0.317787188	-0.518807527
GNAL,NM_001142339	GNAL,NM_182978	0.023850669	-0.008208546	-0.032059215	0.517401911	-0.020654747	-0.538056658
NEDD4,NM_001284340	NEDD4,NM_001284338	-0.009779382	-0.10070794	-0.090928558	0.078782549	-0.054679337	-0.133461886
TCF7L2,NM_001146286	TCF7L2,NM_001198529	-0.032888396	-0.381714213	-0.348825817	0.181173222	-0.347849244	-0.529022467
RABGAP1L,NM_001035230	RABGAP1L,NM_001243763	-0.051898071	-0.055579238	-0.003681168	0.391162983	0.501701346	0.110538363
ADORA2A:SPECC1L:SPECC1L-ADORA2A,NM_001278500	ADORA2A:SPECC1L:SPECC1L-ADORA2A,NR_103543	-0.058926059	-0.33963977	-0.280713712	0.839317367	0.573245444	-0.266071923
DNMT3B,NM_175848	DNMT3B,NM_175849	-0.066730022	0.202001053	0.268731075	1.394563017	0.741498816	-0.653064201
TMEM254,NR_072986	TMEM254,NM_001270373	-0.066760582	-0.121289335	-0.054528753	0.420130282	0.115847747	-0.304282535
TSPAN4,NM_001025237	TSPAN4,NM_001025239	-0.078033756	-0.148875512	-0.070841755	0.379761615	-0.036501841	-0.416263456
BIVM:BIVM-ERCC5:ERCC5,NM_001204425	BIVM:BIVM-ERCC5:ERCC5,NM_017693	-0.148120497	-0.249043487	-0.100922991	0.570071295	-0.08785236	-0.657923655
CLCN3,NM_001243374	CLCN3,NM_173872	-0.18692416	0.203321985	0.390246145	1.048589322	0.688247814	-0.360341508
JAM3,NM_001205329	JAM3,NM_032801	-0.209854781	-0.10492739	0.10492739	0.57608427	0.288042135	-0.288042135
TNFSF12:TNFSF12-TNFSF13:TNFSF13,NM_001198624	TNFSF12:TNFSF12-TNFSF13:TNFSF13,NR_073490	-0.266984445	-0.338641077	-0.071656632	0.919308994	0.592412623	-0.326896371
ZEB1,NM_001174093	ZEB1,NM_030751	-0.284499007	-0.368059885	-0.083560877	1.001611096	0.67214912	-0.329461976
YAP1,NM_001282099	YAP1,NM_001282100	-0.290617779	-0.116314796	0.174302983	0.752852435	0.118064654	-0.634787781
C19orf48,NM_001290154	C19orf48,NM_001290153	-0.316398197	-0.284106808	0.032291389	0.516078677	0.084240409	-0.431838268
CARD8,NM_001184900	CARD8,NR_033679	-0.318545104	0.068865907	0.387411011	0.110670824	0.257698629	0.147027804
BGLAP:PMF1:PMF1-BGLAP,NM_001199654	BGLAP:PMF1:PMF1-BGLAP,NM_001199662	-0.327168399	-0.280117905	0.047050495	0.396686703	0.309985661	-0.086701041
STPG1,NM_001199014	STPG1,NM_001199012	-0.331156459	-0.413467754	-0.082311295	0.400851656	0.120254409	-0.280597247
TRMT112,NM_001286082	TRMT112,NM_001286084	-0.346783114	-0.21742461	0.129358503	0.648176839	0.257647772	-0.390529067
SERPINB6,NM_001271825	SERPINB6,NM_001271824	-0.355346865	0.344065983	0.699412848	0.941832033	0.315847521	-0.625984512
FRS2,NM_001278357	FRS2,NM_001278354	-0.400479023	-0.302587606	0.097891418	0.581091484	0.052106512	-0.528984972
CACNA1G,NR_046055	CACNA1G,NM_198382	-0.412156978	-0.215882279	0.196274698	1.123579417	0.647605143	-0.475974274
CEACAM1,NM_001205344	CEACAM1,NM_001184816	-0.426787325	-0.432452425	-0.005665099	0.648012166	0.403500849	-0.244511317
IL17RC,NM_153460	IL17RC,NM_001203264	-0.46668526	-0.255344992	0.211340269	0.12247427	0.344347101	0.221872831
SLC38A9,NR_047649	SLC38A9,NM_001258286	-0.514651841	-0.103256954	0.411394887	-0.045613872	0.108116968	0.15373084
FKBP9P1,NR_027340	FKBP9P1,NR_027342	-0.541457841	-0.024511228	0.516946614	0.769311704	0.302679689	-0.466632015
INPP4A,NM_001134224	INPP4A,NM_001566	-0.5541761	0.080774206	0.634950306	0.414822563	0.379036604	-0.035785959
EHF,NM_001206615	EHF,NM_001206616	-0.635873914	-0.285450461	0.350423453	0.722896115	0.100904286	-0.62199183
SLC16A3,NM_001042422	SLC16A3,NM_004207	-0.646284949	-0.205842567	0.440442382	0.740352901	0.355663289	-0.384689612
SCO2:TYMP,NM_001169110	SCO2:TYMP,NM_001169109	-0.659797765	-0.330387185	0.329410579	0.219198663	-0.00179384	-0.220992503
TMEM98,NM_015544	TMEM98,NM_001033504	-0.684523496	-0.339580509	0.344942987	1.259811134	0.627081132	-0.632730002
RGL2,NM_001243738	RGL2,NM_004761	-0.84094588	-0.42047294	0.42047294	1.19164992	0.59582496	-0.59582496
NMNAT3,NM_178177	NMNAT3,NM_001200047	-0.990270401	-0.495135198	0.495135203	0.999606913	0.499803456	-0.499803456
ARHGAP17,NM_018054	ARHGAP17,NM_001006634	-1.056036012	-0.528018006	0.528018006	1.451719527	0.725859764	-0.725859764
LFNG,NM_002304	LFNG,NM_001040167	-1.167760479	-0.586560135	0.581200344	0.863604327	0.420468167	-0.44313616
PRR3,NM_025263	PRR3,NM_001077497	-1.257194981	-0.628597491	0.628597491	1.013544633	0.506772317	-0.506772317
LIMK1,NM_001204426	LIMK1,NM_002314	-1.508211368	-0.754105684	0.754105684	0.37106421	0.185532105	-0.185532105

Supplementary Table 5. A list of significant isoform switches (p-value<0.05 & absolute Relative expression median delta>0.1) of event type Alternative 3' Splice Sites (A3) found in ESRP1-low versus ESRP-high cell lines using two-sided Wilcoxon test

Event name	Relative expression median delta	Wilcoxon p-value of relative expression
TMC4;A3:chr19:54675870-54676734:54675852-54676734:-	-0.368975302	0.000835477
MYEOV;A3:chr11:69062043-69062700:69062043-69062868:+	0.175269814	0.001060553
CEP170;A3:chr1:243319757-243327586:243319649-243327586:-	0.109013192	0.001340641
FAM101A:ZNF664:ZNF664-FAM101A;A3:chr12:124458567-124472590:124458567-124472593:+	0.112249386	0.001340641
SYTL2;A3:chr11:85447658-85448618:85447655-85448618:-	0.530248049	0.001687636
CYB561A3;A3:chr11:61117894-61118463:61117115-61118463:-	-0.685502819	0.002641098
HCFC1R1;A3:chr16:3073531-3073848:3073413-3073848:-	0.354163556	0.002641098
PHOSPHO1;A3:chr17:47302493-47304009:47302366-47304009:-	0.25883199	0.002699796
GRK6;A3:chr5:176868054-176868736:176868054-176868738:+	-0.15186873	0.003283461
CDK13;A3:chr7:40127930-40132384:40127930-40132564:+	0.116843848	0.003283461
MKNK2;A3:chr19:2039855-2040133:2037828-2040133:-	0.232890724	0.003283461
CSF1;A3:chr1:110464616-110465788:110464616-110466682:+	-0.366687246	0.005012287
SHOX2;A3:chr3:157816109-157817649:157816073-157817649:-	0.322732964	0.005286959
HEPH;A3:chrX:65483530-65486282:65483530-65486285:+	0.110768722	0.005510642
LOC113230;A3:chr19:14184689-14185137:14184689-14185202:+	-0.222427887	0.007526315
VPS45;A3:chr1:150040007-150040667:150040007-150040687:+	0.152118252	0.007526315
SECISBP2;A3:chr9:91934712-91940342:91934712-91940463:+	0.165883502	0.007526315
MYEOV;A3:chr11:69062043-69062794:69062043-69062868:+	0.166142741	0.007526315
BZRAP1-AS1;A3:chr17:56415040-56429096:56415040-56429105:+	0.25929932	0.007526315
DLG1;A3:chr3:196803556-196807922:196803553-196807922:-	-0.139155209	0.009166074
LOC100506990;A3:chr8:12296020-12298478:12296020-12298509:+	0.18615473	0.009166074
EXOC7;A3:chr17:74086562-74087224:74086478-74087224:-	0.253942979	0.009166074
FAM156A;A3:chrX:52985953-52986286:52985544-52986286:-	-0.220457739	0.011117561
MSTO1:MSTO2P;A3:chr1:155583524-155583850:155583524-155583853:+	-0.190773644	0.011117561
BAZ2B;A3:chr2:160304920-160310124:160304914-160310124:-	0.131498099	0.011117561
ALS2CL;A3:chr3:46717466-46717735:46717176-46717735:-	0.137674548	0.011117561
GTF2I;A3:chr7:74125440-74129177:74125440-74129180:+	0.145075462	0.011117561
REEP2;A3:chr5:137780556-137780917:137780556-137780923:+	-0.324831394	0.011194752
GLRX;A3:chr5:95150661-95152211:95150511-95152211:-	-0.531936481	0.012192849
OLFML2B;A3:chr1:161970131-161976087:161970128-161976087:-	0.499501713	0.012826694
ESR2;A3:chr14:64694647-64701688:64694332-64701688:-	0.502097602	0.013429696
P2RX2;A3:chr12:133196505-133196586:133196505-133196621:+	0.188435481	0.014305878
CACNA1G;A3:chr17:48684350-48685263:48684350-48685290:+	0.164924594	0.015018591
HHLA2;A3:chr3:108070724-108072274:108070724-108072536:+	-0.404218018	0.015806775
LIMA1;A3:chr12:50586347-50589613:50586344-50589613:-	-0.272885398	0.016156931
TM2D2;A3:chr8:38853313-38853732:38852924-38853732:-	0.182334009	0.016156931
PAXIP1-AS2;A3:chr7:154738203-154738303:154738203-154738350:+	0.542502941	0.016156931
TCF4;A3:chr18:53018234-53070685:53018231-53070685:-	-0.227525074	0.017021868
CACNA1G;A3:chr17:48684350-48685263:48684350-48685269:+	0.254302699	0.018204132
IRF4;A3:chr6:395935-397108:395935-397111:+	-0.342014607	0.019016474
MUTYH;A3:chr1:45799233-45800063:45799169-45800063:-	-0.137947837	0.019359467
LTBP4;A3:chr19:41105161-41105308:41105161-41105327:+	0.265207871	0.019359467
CNTF:ZFP91:ZFP91-CNTF;A3:chr11:58377807-58378423:58377807-58378426:+	0.502040455	0.019359467
CES1;A3:chr16:55862886-55866916:55862883-55866916:-	0.12292656	0.020921335
ARMC12;A3:chr6:35715424-35716315:35715424-35716345:+	-0.360354057	0.022990394
FAM188B:INMT:INMT-FAM188B;A3:chr7:30791920-30793347:30791920-30793350:+	-0.499365256	0.023103394
GAS8;A3:chr16:90094130-90097584:90094130-90097707:+	0.114638033	0.023103394
PPP1R3F;A3:chrX:49137924-49138444:49137924-49138447:+	0.268196079	0.023103394
C1S;A3:chr12:7168181-7169056:7168181-7169143:+	-0.28662433	0.025211843
SH2D2A;A3:chr1:156785886-156786467:156785855-156786467:-	-0.173778084	0.026285452
CRHR2;A3:chr7:30705255-30706844:30705252-30706844:-	-0.196324441	0.026453399
DLGAP1;A3:chr18:3582246-3729135:3582162-3729135:-	0.218659254	0.027166252
PTGES2;A3:chr9:130886130-130886771:130886126-130886771:-	-0.222695225	0.027460744
LMBR1L;A3:chr12:49495106-49495255:49495046-49495255:-	-0.202025397	0.027460744
ARHGAP33;A3:chr19:36276385-36277315:36276385-36277798:+	0.124956562	0.027460744
MIB2;A3:chr1:1551994-1558769:1551994-1558811:+	0.228408659	0.027460744
LOC493754;A3:chr7:66041919-66057244:66041916-66057244:-	0.352710703	0.027460744
GPR56;A3:chr16:57662714-57675499:57662714-57675503:+	0.643537439	0.027460744
AP1M2;A3:chr19:10690540-10691942:10690534-10691942:-	-0.279667612	0.029272509
DDX11;A3:chr12:31226903-31231324:31226903-31231397:+	-0.555774519	0.032509445
CCDC51;A3:chr3:48476546-48481506:48476425-48481506:-	-0.296622414	0.032509445
TCF19;A3:chr6:31126499-31126674:31126499-31126920:+	-0.128153573	0.032509445
PXK;A3:chr3:58395886-58398628:58395886-58398631:+	-0.103708706	0.032509445
GUCY1B3;A3:chr4:156721226-156723365:156721226-156723593:+	-0.55721308	0.033894854
GHRL;A3:chr3:10333135-10334526:10332674-10334526:-	0.389084454	0.035296597
SLC29A1;A3:chr6:44193904-44194997:44193904-44195000:+	-0.230354182	0.038333139
MCM8;A3:chr20:5948233-5948476:5948233-5948524:+	-0.129877743	0.038333139
MECR;A3:chr1:29543324-29557243:29543197-29557243:-	0.17211422	0.038333139
TXNDC12;A3:chr1:52499723-52507209:52499600-52507209:-	0.509828199	0.038333139
C6orf48;A3:chr6:31803228-31804190:31803228-31804204:+	0.756450757	0.038333139
C5orf38;A3:chr5:2752578-2752736:2752578-2752794:+	0.174400923	0.038765574
DHRS4L1;A3:chr14:24505837-24507533:24505837-24507616:+	-0.137342271	0.039110519
COL6A2;A3:chr21:47546455-47548817:47546455-47549110:+	0.122660653	0.040423979
FAM213B;A3:chr1:2519892-2520393:2519892-2520396:+	-0.326962381	0.045020881
BTN3A1;A3:chr6:26411809-26412640:26411809-26412933:+	-0.192950114	0.045020881
ICA1;A3:chr7:8272385-8275540:8272382-8275540:-	-0.178886939	0.045020881
HSF4;A3:chr16:67201125-67201364:67201125-67201378:+	-0.165609411	0.045020881
DCAF11;A3:chr14:24584958-24586126:24584958-24586181:+	-0.113181139	0.045020881
ZNF142;A3:chr2:219523853-219524092:219523758-219524092:-	-0.1096334	0.045020881
HSF4;A3:chr16:67201502-67201623:67201502-67201727:+	0.165609411	0.045020881
LETM2;A3:chr8:38250513-38251616:38250513-38251701:+	0.279803326	0.045020881
SLC44A3;A3:chr1:95330455-95332887:95330455-95332890:+	-0.139707619	0.046539581
SLC30A10;A3:chr1:220100485-220101143:220100447-220101143:-	-0.552694805	0.049534613
THPO;A3:chr3:184090954-184091203:184090949-184091203:-	-0.501871658	0.049534613
HNF1B;A3:chr17:36093814-36099431:36093736-36099431:-	0.191205416	0.049534613
LOC151174;A3:chr2:239135253-239136325:239134140-239136325:-	0.993551792	0.049534613

Supplementary Table 6. A list of significant isoform switches (p-value<0.05 & absolute Relative expression median delta>0.1) of event type Alternative 5' Splice Sites (A5) found in ESRP1-low versus ESRP-high cell lines using two-sided Wilcoxon test

Event name	Relative expression median delta	Wilcoxon p-value of relative expression
CTNND1:TMX2:TMX2-CTNND1;A5:chr11:57529591-57556509:57529518-57556509:+	0.745149798	0.000512005
ADAM15;A5:chr1:155034593-155034721:155034451-155034721:+	-0.144628093	0.000835477
DSP;A5:chr6:7581802-7582875:7580005-7582875:+	-0.175780314	0.001060553
SMARCC2;A5:chr12:56557549-56558087:56557549-56558432:-	0.134993701	0.001060553
CEP170;A5:chr1:243303409-243305588:243303409-243305618:-	-0.109013192	0.001340641
FAM101A:ZNF664:ZNF664-FAM101A;A5:chr12:124457899-124458433:124457850-124458433:+	0.110457715	0.001340641
ODF2;A5:chr9:131223346-131231462:131223289-131231462:+	-0.114942381	0.001687636
XKR9;A5:chr8:71581773-71587053:71581709-71587053:+	-0.650453183	0.002115607
IQGAP2;A5:chr5:75905055-75906845:75905004-75906845:+	-0.195516336	0.002843317
ALDH18A1;A5:chr10:97392806-97393248:97392806-97393254:-	-0.248491728	0.003283461
SMARCB1;A5:chr22:24134081-24135746:24134054-24135746:+	-0.121902411	0.00406519
MDP1:NEDD8:NEDD8-MDP1;A5:chr14:24683357-24683468:24683357-24683516:-	-0.198905063	0.005012287
ARPC4:ARPC4-TTLL3:TTLL3;A5:chr3:9868924-9870644:9868756-9870644:+	0.129793831	0.005012287
IQCH-AS1;A5:chr15:67811440-67813832:67811440-67813884:-	-0.331612914	0.006154624
C17orf62;A5:chr17:80407356-80408409:80407356-80408576:-	-0.235458886	0.006154624
COPS7A;A5:chr12:6833416-6833780:6833318-6833780:+	0.321918091	0.006154624
CPEB1;A5:chr15:83221366-83222202:83221366-83222217:-	0.174257513	0.006269685
MORC4;A5:chrX:106184862-106185168:106184862-106185206:-	0.239157171	0.007526315
MARVELD3;A5:chr16:71660599-71663270:71660436-71663270:+	0.548785584	0.007526315
ALS2CL;A5:chr3:46722897-46722990:46722897-46722994:-	-0.201829526	0.009166074
MSTO1:MSTO2P;A5:chr1:155583557-155583853:155583524-155583853:+	-0.182525654	0.009166074
HPS4;A5:chr22:26878206-26879640:26878206-26879731:-	0.108971756	0.009166074
YTHDF3;A5:chr8:64081670-64081946:64081664-64081946:+	0.450898858	0.009166074
SLC44A3;A5:chr1:95293199-95294049:95293103-95294049:+	-0.37705044	0.009873332
C1orf210;A5:chr1:43749039-43751115:43749039-43751224:-	0.496928006	0.010810283
SHISA5;A5:chr3:48538726-48541856:48538726-48542063:-	-0.401388926	0.011117561
ZNF205;A5:chr16:3162951-3163350:3162683-3163350:+	-0.197135552	0.011117561
MAP4K4;A5:chr2:102503723-102504240:102503699-102504240:+	-0.150370297	0.011117561
ZNF202;A5:chr11:123600533-123601195:123600533-123601199:-	-0.145888578	0.011117561
ANAPC11;A5:chr17:79849739-79851428:79849709-79851428:+	0.118938814	0.011117561
ATP8B3;A5:chr19:1791860-1791970:1791860-1792000:-	0.318405813	0.011117561
SLC43A3;A5:chr11:57194125-57194359:57194125-57194539:-	-0.170307603	0.01144592
BDNF;A5:chr11:27680132-27741976:27680132-27742191:-	0.21355182	0.01144592
SDCBP2-AS1;A5:chr20:1306426-1353722:1306173-1353722:+	-0.223892708	0.013429696
P2RX2;A5:chr12:133197174-133197587:133197169-133197587:+	-0.161802566	0.014305878
ZNF501;A5:chr3:44771507-44772968:44771214-44772968:+	-0.504133681	0.014886724
RCAN1;A5:chr21:35896008-35897386:35896008-35897592:-	-0.996994186	0.016156931
FAM107B;A5:chr10:14595386-14613762:14595386-14613923:-	-0.178842394	0.016156931
MAP3K4;A5:chr6:161514883-161518112:161514871-161518112:+	-0.142643017	0.016156931
CYB561D2;A5:chr3:50403288-50403728:50403269-50403728:+	-0.129491256	0.016156931
PDE4DIP;A5:chr1:144881622-144882446:144881622-144882776:-	0.206743123	0.016156931
NR2F1-AS1;A5:chr5:92899169-92916173:92899169-92916848:-	-0.281507859	0.018204132
CACNA1G;A5:chr17:48685401-48692722:48685380-48692722:+	0.51698209	0.018204132
PEX5;A5:chr12:7342812-7342958:7342576-7342958:+	-0.401864121	0.019359467
PICK1;A5:chr22:38453594-38453760:38453496-38453760:+	-0.275306043	0.019359467
SSX2IP;A5:chr1:85146070-85155837:85146070-85156054:-	-0.255146678	0.019359467
MAPK7;A5:chr17:19281943-19282209:19281855-19282209:+	-0.24735815	0.019359467
POLL;A5:chr10:103343438-103344359:103343438-103344635:-	0.104738576	0.019359467
BPTF;A5:chr17:65942441-65943842:65942012-65943842:+	0.127484689	0.019359467
PQBP1;A5:chrX:48755365-48755775:48755297-48755775:+	0.147379218	0.019359467
FAM104B;A5:chrX:55172744-55185555:55172744-55185559:-	0.224245152	0.019359467
BCS1L;A5:chr2:219524968-219525662:219524889-219525662:+	0.264353611	0.019359467
ELOVL5;A5:chr6:53160505-53213148:53160505-53213615:-	0.165814532	0.019404627
CHN2;A5:chr7:29519972-29535568:29519837-29535568:+	0.257050762	0.019517481
ELN;A5:chr7:73470765-73471002:73470687-73471002:+	-0.21086514	0.020637558
CYP4F3;A5:chr19:15751755-15752225:15751739-15752225:+	-0.183565933	0.020716612
ZNF180;A5:chr19:45001424-45004250:45001424-45004327:-	-0.720973205	0.023103394
BCL2L1;A5:chr20:30253889-30309458:30253889-30309647:-	-0.169176575	0.023103394
TANGO2;A5:chr22:20049206-20050861:20049204-20050861:+	-0.123527902	0.023103394
RNF146;A5:chr6:127588240-127601376:127588070-127601376:+	-0.112101359	0.023103394
CCDC90B;A5:chr11:82991303-82996916:82991303-82997143:-	0.107715011	0.023103394
SENP1;A5:chr12:48495299-48499185:48495299-48499240:-	0.136889646	0.023103394
NCOR2;A5:chr12:124810916-124811955:124810916-124812093:-	0.169937318	0.023103394
PPP1R3F;A5:chrX:49127336-49137869:49126396-49137869:+	0.268196079	0.023103394
ENOX1;A5:chr13:44058288-44241578:44058288-44241604:-	0.332356669	0.025144761
CLIP4;A5:chr2:29338531-29344240:29338527-29344240:+	-0.310201358	0.026387678
ZNF503-AS2;A5:chr10:77163815-77167013:77163704-77167013:+	-0.698523007	0.026992286
TNFAIP3;A5:chr6:138188679-138192350:138188655-138192350:+	-0.402139376	0.027460744
KRIT1;A5:chr7:91874485-91874741:91874485-91875104:-	-0.134815611	0.027460744
ZNF321P:ZNF816:ZNF816-ZNF321P;A5:chr19:53459357-53465970:53459357-53465980:-	-0.110963081	0.027460744
MZF1;A5:chr19:59082796-59084422:59082796-59084740:-	-0.100589851	0.027460744
CDK11A:CDK11B;A5:chr1:1577362-1577708:1577362-1577747:-	0.117515836	0.027460744
TMEM218;A5:chr11:124972705-124981435:124972705-124981481:-	0.124412112	0.027460744
COPS7A;A5:chr12:6833416-6833780:6833345-6833780:+	0.209483578	0.027460744
LOC101927027;A5:chr2:179291388-179295497:179291232-179295497:+	0.22439127	0.027460744
C17orf62;A5:chr17:80407356-80408356:80407356-80408409:-	0.817038309	0.027460744
LAMA4;A5:chr6:112508803-112510312:112508803-112510333:-	-0.133122351	0.028185802
SMARCA2;A5:chr9:2158978-2159814:2158553-2159814:+	-0.977589515	0.032509445
THTPA;A5:chr14:24026513-24027904:24026243-24027904:+	-0.306690287	0.032509445
WDR26;A5:chr1:224612356-224619179:224612356-224619227:-	-0.131418556	0.032509445
YAP1;A5:chr11:102076817-102094353:102076805-102094353:+	-0.127167737	0.032509445
TYW3;A5:chr1:75199215-75202226:75199102-75202226:+	0.168190546	0.032509445
PRRT2;A5:chr16:29823672-29824311:29823644-29824311:+	0.483052392	0.032509445
BOC;A5:chr3:112935125-112968569:112935121-112968569:+	-0.160670821	0.033222319

Supplementary Table 6. A list of significant isoform switches (p-value<0.05 & absolute Relative expression median delta>0.1) of event type Alternative 5' Splice Sites (A5) found in ESRP1-low versus ESRP-high cell lines using two-sided Wilcoxon test - **Continued**

Event name	Relative expression median delta	Wilcoxon p-value of relative expression
EMR1;A5:chr19:6928321-6934998:6928222-6934998:+	0.161231323	0.036713856
ARF1;A5:chr1:228271075-228284779:228271053-228284779:+	-0.507896601	0.038333139
PTGR2;A5:chr14:74318823-74325519:74318707-74325519:+	-0.275398218	0.038333139
PTGES2;A5:chr9:130887720-130889718:130887720-130890334:-	-0.263304757	0.038333139
SLC29A1;A5:chr6:44191692-44193798:44191378-44193798:+	-0.230354182	0.038333139
NUDT4:NUDT4P1:NUDT4P2;A5:chr12:93772566-93788385:93772469-93788385:+	-0.223724084	0.038333139
PPP1R7;A5:chr2:242089962-242092891:242089123-242092891:+	-0.139075726	0.038333139
STRADA;A5:chr17:61781407-61781701:61781407-61781792:-	0.140378327	0.038333139
EXOC1;A5:chr4:56720119-56724480:56719963-56724480:+	0.181009113	0.038333139
TOMM5;A5:chr9:37588929-37592250:37588929-37592307:-	0.192195424	0.038333139
GHRLOS;A5:chr3:10323979-10325205:10323918-10325205:+	0.245068576	0.038333139
CCNO;A5:chr5:54528374-54528595:54528374-54528971:-	0.300009952	0.038333139
TMEM139;A5:chr7:142982091-142983517:142982067-142983517:+	0.590529157	0.038333139
C5orf38;A5:chr5:2753484-2755143:2753469-2755143:+	-0.174400923	0.038765574
FMO5;A5:chr1:146696658-146696869:146696658-146696879:-	0.155500273	0.040242785
NETO2;A5:chr16:47156695-47156779:47156695-47156800:-	-0.25171177	0.044423185
STK31;A5:chr7:23750389-23751718:23749954-23751718:+	0.19418768	0.044538616
CNIH2;A5:chr11:66046008-66049730:66046004-66049730:+	-0.481095545	0.045020881
TTC23;A5:chr15:99785715-99789376:99785715-99789622:-	-0.225615301	0.045020881
LSS;A5:chr21:47641889-47642544:47641889-47642577:-	-0.125995186	0.045020881
CSRNP2;A5:chr12:51470430-51477012:51470430-51477122:-	-0.10691664	0.045020881
FBXL6;A5:chr8:145580781-145581099:145580781-145581117:-	0.125505007	0.045020881
DCAF11;A5:chr14:24584958-24586181:24584935-24586181:+	0.199938771	0.045020881
IFIT1;A5:chr10:91153098-91162038:91152960-91162038:+	0.273950487	0.045020881
BUB1B:PAK6;A5:chr15:40532296-40532800:40532003-40532800:+	-0.685708153	0.046539581
LINC01518;A5:chr10:43181464-43186983:43181464-43186987:-	-0.503302014	0.049534613

Supplementary Table 7. A list of significant isoform switches (p-value<0.05 & absolute Relative expression median delta>0.1) of event type Alternative First Exon (AF) found in ESRP1-low versus ESRP-high cell lines using two-sided Wilcoxon test

Event name	Relative expression median delta	Wilcoxon p-value of relative expression
SH3YL1;AF:chr2:253115-260085:261130:253115-263984:264097:-	-0.896020252	0.000308535
FAM65A;AF:chr16:67562720:67562817-67572327:67571365:67571500-67572327:+	-0.458046609	0.000398294
CCDC120;AF:chrX:48910961:48911406-48919490:48916497:48916626-48919490:+	-0.549498905	0.000512005
SH3BP2;AF:chr4:2813946:2814204-2822341:2819912:2820117-2822341:+	-0.281708588	0.000512005
GPR56;AF:chr16:57653910:57654048-57675503:57662138:57662714-57675503:+	0.609431035	0.000655415
RABGAP1L;AF:chr1:174843524:174843644-174846530:174844656:174844851-174846530:+	-0.605322798	0.000835477
RTKN;AF:chr2:74659793-74667480:74667710:74659793-74668833:74669060:-	-0.518397176	0.000835477
C1orf106;AF:chr1:200860627:200860829-200867435:200863949:200864215-200867435:+	-0.38415266	0.001060553
CCDC120;AF:chrX:48910961:48911406-48919490:48916497:48916590-48919490:+	-0.354476569	0.001060553
GNAI2;AF:chr3:50264120:50264625-50289532:50273589:50273885-50289532:+	0.119563175	0.001060553
SMAGP;AF:chr12:51663100-51663843:51663994:51663100-51664040:51664202:-	0.15306024	0.001060553
FHL1;AF:chrX:135228861:135229171-135252066:135229559:135229787-135252066:+	0.161945249	0.001296896
SERINC2;AF:chr1:31883021:31883282-31896540:31886660:31886911-31896540:+	-0.365170376	0.001340641
RNF24;AF:chr20:3944671-3995792:3996054:3944671-3996084:3996216:-	-0.25332296	0.001340641
EHF;AF:chr11:34642588:34642775-34664175:34654011:34654199-34664175:+	0.43814495	0.001687636
SLC44A2;AF:chr19:10713121:10713237-10736929:10736171:10736346-10736929:+	-0.794039759	0.002115607
FAM110A;AF:chr20:814340:814594-825351:821918:821959-825351:+	-0.610479279	0.002115607
DEPDC5;AF:chr22:32149937:32149982-32150848:32150009:32150150-32150848:+	-0.295117132	0.002115607
DFNA5;AF:chr7:24789412-24797015:24797083:24789412-24797326:24797639:-	-0.265706268	0.002345787
GPR56;AF:chr16:57653605:57653793-57684165:57662138:57662714-57684165:+	0.905436752	0.002506463
SH3YL1;AF:chr2:262786-264703:264743:262786-264782:264866:-	-0.191790304	0.002641098
MID1;AF:chrX:10535643-10645505:10645779:10535643-10851673:10851809:-	-0.186896902	0.002641098
PRKACB;AF:chr1:84609931:84610231-84644860:84629867:84630105-84644860:+	0.78523415	0.002843317
FHL1;AF:chrX:135228861:135229171-135252066:135230737:135230896-135252066:+	0.20897045	0.002985471
HEPH;AF:chrX:65382391:65382718-65390400:65384072:65384312-65390400:+	0.607477825	0.003866534
FHL1;AF:chrX:135229559:135229787-135252066:135230737:135230896-135252066:+	-0.440536138	0.003892417
PIGO;AF:chr9:35095563-35096152:35096546:35095563-35096558:35096598:-	0.234931007	0.005012287
BRSK2;AF:chr11:1431673:1432136-1457270:1432382:1432863-1457270:+	0.600472545	0.005012287
ZNF32;AF:chr10:44141650-44144033:44144152:44141650-44144250:44144326:-	-0.137681371	0.005362948
ARHGAP8:PRR5:PRR5-ARHGAP8;AF:chr22:45064427:45064685-45098372:45072688:45073120-45098372:+	-0.929677723	0.006154624
MARK2;AF:chr11:63606400:63607032-63662631:63655987:63656436-63662631:+	-0.374424963	0.006154624
SPECC1;AF:chr17:19912649:19912737-19999944:19990335:19990364-19999944:+	-0.30712971	0.006154624
TMEM79;AF:chr1:156252704:156252856-156254975:156254070:156254197-156254975:+	-0.286285251	0.006154624
PREPL;AF:chr2:44573529-44586636:44587813:44573529-44588663:44589001:-	0.272821469	0.006154624
CRIP2;AF:chr14:105939275:105939381-105944603:105943583:105943938-105944603:+	0.490548333	0.006154624
SCO2:TYMP;AF:chr22:50962853-50964430:50964574:50962853-50964675:50964868:-	0.848270501	0.006154624
SLC44A3;AF:chr1:95285898:95286034-95290049:95286100:95286279-95290049:+	-0.557443519	0.006378219
GPR56;AF:chr16:57662138:57662740-57675503:57673207:57673582-57675503:+	-0.246845578	0.006378219
SLC43A3;AF:chr11:57194125-57194359:57194560:57194125-57195016:57195053:-	-0.332401041	0.006605619
HIC1;AF:chr17:1958393:1958532-1959965:1959604:1959640-1959965:+	-0.549430334	0.006649756
TMEM254;AF:chr10:81838402:81838542-81841597:81838779:81838940-81841597:+	-0.614331312	0.007526315
NFIX;AF:chr19:13106584:13106678-13135835:13134785:13134848-13135835:+	-0.211321512	0.007526315
BZRAP1-AS1;AF:chr17:56406966:56407025-56414809:56414563:56414671-56414809:+	0.336650956	0.007526315
SLC16A3;AF:chr17:80186282:80186405-80193859:80186887:80186982-80193859:+	0.653972864	0.007526315
BDNF;AF:chr11:27680132-27720929:27721214:27680132-27722223:27722447:-	0.251931046	0.008730257
RIMKLB;AF:chr12:8834273:8834414-8866407:8850496:8850893-8866407:+	0.252801776	0.009023439
GSN;AF:chr9:124048359:124048463-124064241:124048858:124049039-124064241:+	-0.600987252	0.009166074
TJP3;AF:chr19:3708335:3708559-3728422:3721697:3721908-3728422:+	-0.24902283	0.009166074
SLC35B2;AF:chr6:44224615-44224919:44225089:44224615-44225137:44225308:-	-0.132821715	0.009166074
MDK;AF:chr11:46402334:46402733-46403607:46403205:46403308-46403607:+	-0.104072234	0.009166074
SERPINB6;AF:chr6:2959576-2962128:2962404:2959576-2971725:2972399:-	0.610338801	0.009166074
RASGRP2;AF:chr11:64510409-64511475:64511630:64510409-64512214:64512329:-	-0.34614749	0.009207901
LYNX1;AF:chr8:143857537-143858330:143858437:143857537-143858522:143858744:-	0.517820462	0.009236735
CLDN2;AF:chrX:106143394:106143734-106171281:106163606:106163781-106171281:+	-0.388459152	0.010024846
FHL1;AF:chrX:135230737:135230896-135252066:135251796:135251892-135252066:+	0.311269856	0.010562873
COLCA2;AF:chr11:111169271:111169313-111171252:111169565:111169783-111171252:+	0.284548914	0.010803685
BZRAP1-AS1;AF:chr17:56406299:56406435-56414809:56406966:56407025-56414809:+	-0.472807771	0.011117561
NUDT4:NUDT4P1:NUDT4P2;AF:chr12:93771746:93772197-93788385:93772326:93772469-93788385:+	-0.355993883	0.011117561
KCNIP2;AF:chr10:103588956-103599510:103599611:103588956-103603253:103603677:-	0.24793713	0.011117561
PTGES2;AF:chr9:130887720-130890334:130890467:130887720-130890527:130890741:-	0.296540074	0.011117561
BANP;AF:chr16:87993477:87993599-88008654:88003624:88003776-88008654:+	0.751794208	0.011117561
SEPT4;AF:chr17:56604339-56605187:56605408:56604339-56609322:56609445:-	0.255390657	0.011169835
BDNF;AF:chr11:27680132-27741058:27741294:27680132-27741976:27742326:-	-0.273297594	0.01144592
BDNF;AF:chr11:27680132-27741058:27741294:27680132-27742191:27742326:-	0.27457155	0.01144592
UGT1A1:UGT1A10:UGT1A3:UGT1A4:UGT1A5:UGT1A6:UGT1A7:UGT1A8:UGT1A9;AF:chr2:234590584:234591438-234675680:234637773:234638639-234675680:+	-0.919872275	0.012334708
SORBS2;AF:chr4:186696520-186732092:186732258:186696520-186877350:186877870:-	0.638294642	0.012738072
CLDN2;AF:chrX:106143394:106143734-106171281:106161590:106161699-106171281:+	-0.381593477	0.012857794
GPR56;AF:chr16:57662138:57662714-57675503:57673207:57673582-57675503:+	-0.844859469	0.013429696
RALGDS;AF:chr9:135987539-135996359:135996637:135987539-136006339:136006544:-	-0.149210912	0.013429696
SYTL2;AF:chr11:85459466-85468668:85469112:85459466-85521997:85522202:-	0.67595938	0.013429696
GPR56;AF:chr16:57653605:57653793-57675503:57662138:57662714-57675503:+	0.999028223	0.013429696
FAM110A;AF:chr20:814340:814594-825351:816711:816757-825351:+	-0.31462473	0.0147378
BDNF;AF:chr11:27680132-27722528:27722600:27680132-27741976:27742326:-	-0.16514322	0.014886724
KLRC4:KLRC4-KLRK1:KLRK1;AF:chr12:10561609-10561988:10562356:10561609-10562698:10562745:-	-0.746354252	0.015158439
NELL2;AF:chr12:45269683-45270390:45270633:45269683-45307582:45307711:-	0.255940871	0.015806775
NR5A2;AF:chr1:199996730:199997039-200012902:200011953:200012093-200012902:+	0.364784982	0.016078346
DLK2;AF:chr6:43422618-43423133:43423362:43422618-43423725:43424370:-	-0.712678173	0.016156931
TMEM254;AF:chr10:81838402:81838542-81841597:81839001:81839063-81841597:+	-0.456257267	0.016156931
RASSF7;AF:chr11:560971:561033-561762:561450:561477-561762:+	-0.143856789	0.016156931
ACSL5;AF:chr10:114133916:114134027-114154676:114135956:114136206-114154676:+	0.178062352	0.016156931
CORO2B;AF:chr15:68908879:68908953-68937499:68924327:68924680-68937499:+	-0.344965739	0.016309172
SCOC;AF:chr4:141264615:141264862-141300275:141294664:141294871-141300275:+	0.369020644	0.016377308
ZNF503-AS2;AF:chr10:77163514:77163815-77167013:77163923:77164042-77167013:+	-0.657546412	0.016578175

Supplementary Table 7. A list of significant isoform switches (p-value<0.05 & absolute Relative expression median delta>0.1) of event type Alternative First Exon (AF) found in ESRP1-low versus ESRP-high cell lines using two-sided Wilcoxon test -**Continued**

Event name	Relative expression median delta	Wilcoxon p-value of relative expression
GRHL3;AF:chr1:24646056:24646237-24657916:24649530:24649607-24657916:+	-0.389732328	0.016638679
FHL1;AF:chrX:135278913:135279022-135288566:135279055:135279293-135288566:+	-0.140993708	0.016762336
RIMKLB;AF:chr12:8834273:8834414-8866407:8852381:8853210-8866407:+	0.116105519	0.016970294
C2orf54;AF:chr2:241831180-241831290:241831455:241831180-241834901:241835573:-	-0.976001453	0.018554892
C2orf54;AF:chr2:241831180-241831347:241831455:241831180-241834901:241835573:-	-0.903182684	0.018554892
BDNF;AF:chr11:27680132-27722518:27722600:27680132-27741058:27741294:-	-0.258460541	0.019208251
BDNF;AF:chr11:27680132-27722518:27722600:27680132-27742191:27742326:-	-0.215343049	0.019208251
BDNF;AF:chr11:27680132-27722518:27722600:27680132-27741976:27742326:-	-0.172068435	0.019208251
RAD17;AF:chr5:68665124:68665291-68666862:68665639:68665760-68666862:+	-0.296348804	0.019359467
RNF14;AF:chr5:141346402:141346469-141350269:141348451:141348732-141350269:+	0.110762114	0.019359467
GYLTL1B;AF:chr11:45943172:45943244-45944372:45944227:45944279-45944372:+	0.203652142	0.019359467
SLC22A23;AF:chr6:3416089-3445053:3445247:3416089-3456140:3456793:-	0.223461213	0.019359467
PEX11B;AF:chr1:145516165:145516452-145517273:145516560:145516657-145517273:+	0.253725279	0.019359467
LTBP4;AF:chr19:41099072:41099087-41105103:41103141:41103286-41105103:+	0.265207871	0.019359467
CASP8;AF:chr2:202122754:202123105-202131184:202125223:202125336-202131184:+	0.332153401	0.019359467
FOXP1;AF:chr3:71630848-71632722:71632904:71630848-71633061:71633140:-	0.345230423	0.019359467
SESN1;AF:chr6:109323546-109330562:109330758:109323546-109414998:109415708:-	0.376628405	0.019359467
CTAGE5;AF:chr14:39734476:39734625-39746138:39736328:39736726-39746138:+	0.397045717	0.019359467
ZFAT;AF:chr8:135687131-135708674:135708801:135687131-135725088:135725292:-	0.488938709	0.019359467
DSE;AF:chr6:116601283:116601423-116720361:116692110:116692284-116720361:+	0.58885218	0.019359467
PRKACB;AF:chr1:84543658:84544054-84644860:84609931:84610231-84644860:+	-0.600626775	0.020736608
PHLDB2:PLCXD2;AF:chr3:111578027:111578423-111602911:111578568:111578725-111602911:+	-0.313220034	0.020776366
CHN2;AF:chr7:29234121:29234606-29394241:29237357:29237599-29394241:+	-0.409806492	0.021966365
PDE4B;AF:chr1:66258193:66258313-66378928:66258856:66258931-66378928:+	-0.240474471	0.022740296
MINOS1:MINOS1-NBL1:NBL1;AF:chr1:19970208:19970448-19981505:19971866:19972024-19981505:+	-0.655773667	0.023103394
BBC3;AF:chr19:47731703-47734186:47734451:47731703-47735772:47736023:-	-0.533904773	0.023103394
SH3YL1;AF:chr2:253115-260085:261130:253115-264084:264392:-	-0.399163108	0.023103394
ICA1;AF:chr7:8275635-8301724:8301911:8275635-8302070:8302242:-	-0.378123012	0.023103394
KIAA0586;AF:chr14:58894103:58894355-58896081:58894710:58895181-58896081:+	-0.244690788	0.023103394
ARHGAP8:PRR5:PRR5-ARHGAP8;AF:chr22:45064427:45064685-45110471:45072688:45073120-45110471:+	-0.127410072	0.023103394
KATNAL1;AF:chr13:30857928-30881077:30881191:30857928-30881468:30881624:-	0.143237194	0.023103394
MINOS1:MINOS1-NBL1:NBL1;AF:chr1:19923471:19923603-19981505:19970208:19970448-19981505:+	0.26933054	0.023103394
SRC;AF:chr20:35973088:35973290-35993607:35974531:35974658-35993607:+	0.389429481	0.023103394
SLC12A6;AF:chr15:34567590-34610762:34611021:34567590-34628611:34629045:-	0.467063849	0.023103394
WNT2B;AF:chr1:113009163:113010213-113057496:113051370:113052066-113057496:+	0.61365265	0.023103394
SH3YL1;AF:chr2:253115-264084:264392:253115-264782:264866:-	0.757449007	0.023103394
ADAMTSL2;AF:chr9:136397286:136397692-136401685:136399975:136400039-136401685:+	-0.231241749	0.023342202
NELL2;AF:chr12:45269683-45270077:45270247:45269683-45270390:45270633:-	-0.282840411	0.02396768
SLC43A3;AF:chr11:57194125-57194539:57194621:57194125-57195016:57195053:-	-0.211100016	0.024588566
BDNF;AF:chr11:27680132-27720929:27721214:27680132-27721711:27722035:-	0.515508212	0.024588566
CORO2B;AF:chr15:68871308:68871616-68937499:68924327:68924680-68937499:+	-0.469463776	0.024974679
UGT1A1:UGT1A10:UGT1A3:UGT1A4:UGT1A5:UGT1A6:UGT1A7:UGT1A8:UGT1A9;AF:chr2:234590584:234591438-234675680:234668919:234669797-234675680:+	-0.944307087	0.026387678
ZNF790;AF:chr19:37316594-37328574:37328929:37316594-37329239:37329286:-	0.454557216	0.026430974
COLCA2;AF:chr11:111169565:111169783-111171252:111169976:111170449-111171252:+	-0.407221568	0.026856696
FBLN2;AF:chr3:13590625:13590708-13611815:13610240:13610580-13611815:+	-0.159617545	0.027030077
HRH1;AF:chr3:11196214:11196240-11300689:11267669:11267745-11300689:+	-0.319507233	0.027460744
CREB3L4;AF:chr1:153940315:153940660-153940998:153940675:153940786-153940998:+	-0.148660797	0.027460744
RNF14;AF:chr5:141346402:141346469-141350269:141348712:141348799-141350269:+	0.104338161	0.027460744
BIVM:BIVM-ERCC5:ERCC5;AF:chr13:103451399:103451871-103468778:103459496:103460095-103468778:+	0.134671662	0.027460744
FGD5-AS1;AF:chr3:14987661-14989246:14989400:14987661-14989520:14989948:-	0.150630649	0.027460744
CTAGE5;AF:chr14:39735502:39735731-39746138:39736328:39736726-39746138:+	0.168056642	0.027460744
SLC20A2;AF:chr8:42330172-42358549:42358978:42330172-42396964:42397356:-	0.401965269	0.027460744
TMEM51;AF:chr1:15479028:15479280-15541391:15480229:15480450-15541391:+	0.451970344	0.027460744
SERPINB6;AF:chr6:2959576-2962128:2962404:2959576-2971482:2972399:-	0.517943019	0.027460744
PSCA;AF:chr8:143751726:143751986-143762745:143761874:143761981-143762745:+	-0.253777323	0.029272509
GJB1;AF:chrX:70435062:70435140-70443542:70443056:70443185-70443542:+	0.409053425	0.02991299
LYNX1;AF:chr8:143857537-143858330:143858437:143857537-143859175:143859640:-	0.532580714	0.030059568
BDNF;AF:chr11:27680132-27722223:27722447:27680132-27741058:27741294:-	-0.47503176	0.031228617
BDNF;AF:chr11:27680132-27722844:27723180:27680132-27742959:27743605:-	-0.131250184	0.031228617
GFOD1;AF:chr6:13365894-13408142:13408369:13365894-13486243:13486415:-	-0.123788491	0.031557786
LOC100288798;AF:chr12:46777459:46777478-46781530:46777890:46777944-46781530:+	0.214691858	0.031557786
RGS20;AF:chr8:54764368:54764624-54852136:54793432:54793644-54852136:+	0.418864015	0.031794861
RCAN1;AF:chr21:35896008-35897386:35897765:35896008-35986577:35986745:-	-0.998444471	0.032509445
IFIT3;AF:chr10:91087576:91087835-91098418:91092236:91092384-91098418:+	-0.493733024	0.032509445
PCDHGA1:PCDHGA10:PCDHGA11:PCDHGA12:PCDHGA2:PCDHGA3:PCDHGA4:PCDHGA5:PCDHGA6:PCDHGA7:PCDHGA8:PCDHGA9:PCDHGB1:PCDHGB2:PCDHGB3:PCDHGB4:PCDHGB5:PCDHGB6:PCDHGB7:PCDHGC3:PCDHGC4:PCDHGC5;AF:chr5:140723601:140726024-140874374:140787770:140790187-140874374:+	-0.412373007	0.032509445
PRKAR1A;AF:chr17:66507921:66508283-66511535:66508520:66508720-66511535:+	-0.318872091	0.032509445
CSPG5;AF:chr3:47619418-47620087:47620359:47619418-47621380:47621730:-	-0.271682332	0.032509445
DNMT3B;AF:chr20:31350191:31350505-31368124:31367658:31367951-31368124:+	-0.233748821	0.032509445
YY1AP1;AF:chr1:155657992-155658450:155658568:155657992-155658721:155658823:-	-0.171680719	0.032509445
RALGDS;AF:chr9:135987539-136004526:136004788:135987539-136006339:136006544:-	-0.152108711	0.032509445
ZEB1;AF:chr10:31608101:31608221-31749966:31610064:31610461-31749966:+	-0.141172149	0.032509445
NSUN4;AF:chr1:46805849:46806591-46810473:46806850:46807537-46810473:+	0.112123701	0.032509445
BUB1B:PAK6;AF:chr15:40509629:40509868-40532800:40531292:40532003-40532800:+	-0.942495315	0.032524181
GPR56;AF:chr16:57653605:57653793-57684165:57673207:57673582-57684165:+	0.5952643	0.032524181
RAI2;AF:chrX:17820154-17878629:17878947:17820154-17879218:17879457:-	-0.352561616	0.033006258
FAM65C;AF:chr20:49247393-49253118:49253455:49247393-49307663:49308067:-	0.437018019	0.033222319
SNCA;AF:chr4:90756843-90758113:90758350:90756843-90759403:90759447:-	-0.530915156	0.033894854
SNCA;AF:chr4:90756843-90757894:90758127:90756843-90759403:90759447:-	-0.360056932	0.033894854
STRA6;AF:chr15:74494623-74495344:74495556:74494623-74501141:74501371:-	0.272769977	0.035014981
BDNF;AF:chr11:27680132-27722223:27722447:27680132-27741976:27742326:-	-0.317780273	0.035090018
MEST;AF:chr7:130131170:130131327-130135209:130131899:130132180-130135209:+	0.365632722	0.037573552

Supplementary Table 7. A list of significant isoform switches (p-value<0.05 & absolute Relative expression median delta>0.1) of event type Alternative First Exon (AF) found in ESRP1-low versus ESRP-high cell lines using two-sided Wilcoxon test -**Continued**

Event name	Relative expression median delta	Wilcoxon p-value of relative expression
RCAN1;AF:chr21:35896008-35897386:35897765:35896008-35986086:35986142:-	-0.886892765	0.038333139
CLIP4;AF:chr2:29320542:29320813-29344240:29338292:29338527-29344240:+	-0.765451862	0.038333139
GCNT1;AF:chr9:79056582:79057231-79115832:79093257:79093446-79115832:+	-0.362120278	0.038333139
KLHL2;AF:chr4:166128770:166129054-166141086:166131171:166131332-166141086:+	-0.274177047	0.038333139
CYTH1;AF:chr17:76705814-76713011:76713114:76705814-76778284:76778424:-	0.101278469	0.038333139
SHISA5;AF:chr3:48538726-48541452:48541682:48538726-48541856:48542247:-	0.109669608	0.038333139
PNPLA4;AF:chrX:7894173-7895327:7895475:7894173-7895727:7895780:-	0.135431486	0.038333139
NFATC2;AF:chr20:50140649-50158909:50159258:50140649-50179099:50179370:-	0.174207534	0.038333139
PPP1R13L;AF:chr19:45901597-45908257:45908312:45901597-45909550:45909607:-	0.223093688	0.038333139
MID1;AF:chrX:10535643-10544824:10544957:10535643-10645505:10645779:-	0.297153548	0.038333139
PLA2G6;AF:chr22:38565478-38577671:38577761:38565478-38577791:38577836:-	0.303885563	0.038333139
PRKAR1A;AF:chr17:66409764:66409936-66511535:66507921:66508283-66511535:+	0.305163481	0.038333139
PRKAG2;AF:chr7:151483627-151511502:151511957:151483627-151573592:151574316:-	0.334230078	0.038333139
SH3YL1;AF:chr2:253115-263984:264097:253115-264782:264866:-	0.57574925	0.038333139
DDR1;AF:chr6:30850390:30850760-30856465:30852315:30852487-30856465:+	0.639270653	0.038333139
ACOT7;AF:chr1:6409926-6420630:6420764:6409926-6445554:6445883:-	0.808560074	0.038333139
LSP1;AF:chr11:1891482:1891891-1901317:1892099:1892642-1901317:+	0.543393383	0.038867104
SYNPO2;AF:chr4:119771843:119772095-119944585:119809996:119810296-119944585:+	0.505165194	0.039211045
BDNF;AF:chr11:27680132-27721729:27722035:27680132-27742959:27743605:-	-0.252352455	0.039351837
BDNF;AF:chr11:27680132-27720929:27721214:27680132-27741976:27742326:-	-0.226756719	0.039351837
BDNF;AF:chr11:27680132-27720929:27721214:27680132-27722844:27723180:-	0.51557884	0.039351837
SORBS2;AF:chr4:186696520-186732092:186732258:186696520-186732818:186733410:-	0.353011289	0.041540067
SORBS2;AF:chr4:186696520-186696856:186697066:186696520-186877350:186877870:-	0.570693816	0.041540067
FGFR1;AF:chr8:38315052-38325190:38325363:38315052-38325499:38326352:-	-0.253521273	0.043311496
DAPK1;AF:chr9:90112596:90113022-90113885:90113450:90113551-90113885:+	-0.241662467	0.043975196
BDNF;AF:chr11:27680132-27721729:27722035:27680132-27741976:27742326:-	-0.433429208	0.044045295
BDNF;AF:chr11:27680132-27722223:27722447:27680132-27741893:27742326:-	-0.219878172	0.044045295
UGT1A1:UGT1A10:UGT1A3:UGT1A4:UGT1A5:UGT1A6:UGT1A7:UGT1A8:UGT1A9;AF:chr2:234590584:234591438-234675680:234601512:234602511-234675680:+	-0.621340891	0.044538616
MYADM;AF:chr19:54369412:54369671-54373008:54371119:54371202-54373008:+	-0.546254274	0.045020881
PTCH1;AF:chr9:98268881-98270443:98270831:98268881-98278905:98279247:-	-0.45241174	0.045020881
DDR1;AF:chr6:30852315:30852487-30856465:30852757:30853008-30856465:+	-0.106491859	0.045020881
EXD2;AF:chr14:69658194:69658308-69670634:69658483:69658756-69670634:+	-0.10134907	0.045020881
PACSIN2;AF:chr22:43308163-43355805:43355887:43308163-43411027:43411184:-	0.102476783	0.045020881
GNA13;AF:chr17:63049846-63051711:63051892:63049846-63052429:63052920:-	0.122065789	0.045020881
AK3;AF:chr9:4722625-4726083:4726227:4722625-4740937:4741309:-	0.13496397	0.045020881
AKT1S1;AF:chr19:50376559-50379131:50379506:50376559-50379860:50380644:-	0.211310288	0.045020881
ACOT7;AF:chr1:6409926-6420630:6420764:6409926-6453317:6453826:-	0.284933356	0.045020881
SERPINB6;AF:chr6:2959576-2970992:2971282:2959576-2971725:2972399:-	0.317995396	0.045020881
MYADM;AF:chr19:54371119:54371202-54373008:54371799:54371913-54373008:+	0.346420387	0.045020881
SERPINB6;AF:chr6:2959576-2962128:2962404:2959576-2970992:2971282:-	0.445802915	0.045020881
PCDHGA1:PCDHGA10:PCDHGA11:PCDHGA12:PCDHGA2:PCDHGA3:PCDHGA4:PCDHGA5:PCDHGA6:PCDHGA7:PCDHGA8:PCDHGA9:PCDHGB1:PCDHGB2:PCDHGB3:PCDHGB4:PCDHGB5:PCDHGB6:PCDHGB7:PCDHGC3:PCDHGC4:PCDHGC5;AF:chr5:140787770:140790187-140874374:140855569:140858113-140874374:+	0.477207835	0.045020881
PCDHGA1:PCDHGA10:PCDHGA11:PCDHGA12:PCDHGA2:PCDHGA3:PCDHGA4:PCDHGA5:PCDHGA6:PCDHGA7:PCDHGA8:PCDHGA9:PCDHGB1:PCDHGB2:PCDHGB3:PCDHGB4:PCDHGB5:PCDHGB6:PCDHGB7:PCDHGC3:PCDHGC4:PCDHGC5;AF:chr5:140753651:140756074-140874374:140855569:140858113-140874374:+	0.63349834	0.045020881
NEBL-AS1;AF:chr10:21462919:21463036-21463411:21463231:21463302-21463411:+	0.434813339	0.045500264
PHYHIPL;AF:chr10:60936348:60936719-60994064:60937227:60937378-60994064:+	0.538768464	0.045500264
PCDHA1:PCDHA10:PCDHA11:PCDHA12:PCDHA13:PCDHA2:PCDHA3:PCDHA4:PCDHA5:PCDHA6:PCDHA7:PCDHA8:PCDHA9:PCDHAC1:PCDHAC2;AF:chr5:140235468:140237232-140358534:140345747:140348916-140358534:+	0.544515119	0.047125304
MB;AF:chr22:36013312-36013409:36013555:36013312-36019238:36019401:-	0.510963509	0.047418372
PCDHA1:PCDHA10:PCDHA11:PCDHA12:PCDHA13:PCDHA2:PCDHA3:PCDHA4:PCDHA5:PCDHA6:PCDHA7:PCDHA8:PCDHA9:PCDHAC1:PCDHAC2;AF:chr5:140174444:140176937-140358534:140306302:140308910-140358534:+	0.514652015	0.047418372
BDNF;AF:chr11:27680132-27722518:27722600:27680132-27741893:27742326:-	-0.123760122	0.049202789
CLDN2;AF:chrX:106161590:106161699-106171281:106163606:106163781-106171281:+	0.38247201	0.049534613

Supplementary Table 8. A list of significant isoform switches (p-value<0.05 & absolute Relative expression median delta>0.1) of event type Alternative First Exon (AF) found in ESRP1-low versus ESRP-high cell lines using two-sided Wilcoxon test

Event name	Relative expression median delta	Wilcoxon p-value of relative expression
KRTCAP3;AL:chr2:27666928-27667053:27667164:27666928-27669168:27669348:+	0.267379001	0.001339739
PSG6;AL:chr19:43406234:43406598-43411074:43407768:43407873-43411074:-	-0.160188803	0.00448325
ZNF254;AL:chr19:24289445-24290018:24290154:24289445-24309056:24312769:+	-0.083858871	0.004807913
C22orf39;AL:chr22:19428410:19429217-19434901:19430673:19431913-19434901:-	0.180053447	0.005012287
FGFR2;AL:chr10:123237844:123239535-123243212:123241367:123241691-123243212:-	-0.038915802	0.006154624
LOC100506990;AL:chr8:12388540-12406996:12408558:12388540-12423395:12424354:+	0.18615473	0.009166074
THAP7-AS1;AL:chr22:21357118-21357207:21357561:21357118-21362956:21364663:+	0.233123817	0.009166074
BCL7C;AL:chr16:30845362:30846469-30900181:30899116:30899311-30900181:-	-0.017807045	0.011117561
CDCA3;AL:chr12:6955889:6956067-6958729:6957972:6958362-6958729:-	0.280879297	0.013429696
NIN;AL:chr14:51192546:51192784-51196241:51193951:51194469-51196241:-	0.133252922	0.015018591
PPCS;AL:chr1:42923021-42925274:42926901:42923021-42938793:42939049:+	-0.04482526	0.016156931
FAM86B3P;AL:chr8:8094797-8095832:8096448:8094797-8097149:8097552:+	0.311601056	0.016156931
SVIL-AS1;AL:chr10:29704341-29757849:29758059:29704341-29776084:29776785:+	-0.803238109	0.020210931
SEPT6;AL:chrX:118749688:118750705-118763281:118750909:118752749-118763281:-	0.595329543	0.021966365
SVIL-AS1;AL:chr10:29704341-29709394:29711301:29704341-29757849:29758059:+	0.99998853	0.022292295
SVIL-AS1;AL:chr10:29704341-29757849:29758059:29704341-29771762:29774636:+	-0.412580733	0.023103394
LOC644919;AL:chr14:41443595-41494725:41494994:41443595-41610037:41610251:+	0.193477629	0.027248441
LIMK2;AL:chr22:31671294-31672775:31673624:31671294-31674283:31676066:+	-0.12068326	0.027460744
POM121;AL:chr7:72416247-72416676:72418843:72416247-72418867:72421979:+	-0.027940942	0.027460744
SVIL-AS1;AL:chr10:29704341-29709394:29711301:29704341-29771762:29774636:+	0.313129715	0.027460744
BAIAP2;AL:chr17:79082309-79082768:79084312:79082309-79089570:79091232:+	0.187314809	0.032509445
TSFM;AL:chr12:58186856-58189960:58191370:58186856-58196338:58196639:+	-0.103463851	0.038333139
CGREF1;AL:chr2:27322221:27322708-27324965:27323462:27324756-27324965:-	-0.079718459	0.043975196
FBLN1;AL:chr22:45946495-45953951:45954591:45946495-45960764:45961581:+	0.35597011	0.044423185
NF2;AL:chr22:30077590-30079009:30079904:30077590-30090741:30094589:+	0.227039998	0.045020881

Supplementary Table 9. A list of significant isoform switches (p-value<0.05 & absolute Relative expression median delta>0.1) of event type Mutually Exclusive Exon (MX) found in ESRP1-low versus ESRP-high cell lines using two-sided Wilcoxon test

Event name	Relative expression median delta	Wilcoxon p-value of relative expression
EXOC7;MX:chr17:74085401-74086410:74086478-74090495:74085401-74087224:74087316-74090495:-	0.778616202	0.000237997
TCF7L2;MX:chr10:114917828-114918426:114918476-114925314:114917828-114920378:114920450-114925314:+	-0.308669373	0.000655415
DNM2;MX:chr19:10906115-10906737:10906875-10909162:10906115-10908056:10908194-10909162:+	0.17723361	0.000655415
FGFR3;MX:chr4:1803752-1804641:1804791-1806057:1803752-1805419:1805563-1806057:+	0.394181172	0.000835477
TCF7L2;MX:chr10:114917828-114919679:114919751-114925314:114917828-114920378:114920450-114925314:+	-0.20099847	0.002115607
DLG1;MX:chr3:196796131-196802708:196802741-196807922:196796131-196803457:196803556-196807922:-	0.204488848	0.002115607
CACNA1G;MX:chr17:48696369-48697044:48697187-48701268:48696369-48699021:48699155-48701268:+	0.708014581	0.002736753
TSPAN4;MX:chr11:842915-847201:847300-862550:842915-850288:850367-862550:+	-0.484202773	0.00406519
MARK3;MX:chr14:103958371-103964839:103964865-103969219:103958371-103966493:103966537-103969219:+	-0.416682671	0.00406519
YAF2;MX:chr12:42555567-42592938:42593037-42631401:42555567-42604350:42604421-42631401:-	-0.472389037	0.008123423
MUC1;MX:chr1:155159850-155159931:155160052-155160484:155159850-155160198:155160334-155160484:-	-0.973219917	0.009166074
CKLF:CKLF-CMTM1:CMTM1;MX:chr16:66586696-66592093:66592251-66599789:66586696-66597025:66597120-66599789:+	0.146953056	0.009166074
IFIT1;MX:chr10:91152474-91152864:91153098-91162038:91152474-91159652:91159760-91162038:+	0.22093819	0.009166074
FRS2;MX:chr12:69864310-69874079:69874139-69924645:69864310-69885318:69885431-69924645:+	0.342686486	0.009166074
MBNL1;MX:chr3:152165562-152173331:152173366-152177060:152165562-152174056:152174150-152177060:+	-0.198642953	0.013429696
FXYD2:FXYD6:FXYD6-FXYD2;MX:chr11:117713496-117714825:117714921-117747307:117713496-117728926:117729001-117747307:-	0.172762631	0.014305878
CHFR;MX:chr12:133438220-133438919:133439011-133447310:133438220-133446205:133446420-133447310:-	-0.214441614	0.019208251
FGFR2;MX:chr10:123274833-123276833:123276977-123279493:123274833-123278196:123278343-123279493:-	-0.547726346	0.019359467
BACE2;MX:chr21:42617990-42622679:42622828-42647298:42617990-42629085:42629253-42647298:+	-0.337957668	0.019359467
AKAP13;MX:chr15:86199018-86201768:86201821-86207794:86199018-86205619:86205684-86207794:+	-0.206535754	0.019359467
FRS2;MX:chr12:69864310-69879982:69880077-69924645:69864310-69885318:69885431-69924645:+	0.106665413	0.019359467
CACNB1;MX:chr17:37341117-37341384:37341403-37342749:37341117-37342203:37342357-37342749:-	0.142042744	0.019359467
IFIT1;MX:chr10:91152474-91152864:91153098-91162038:91152474-91159647:91159760-91162038:+	0.177453417	0.019359467
GSR;MX:chr8:30541716-30546678:30546836-30553897:30541716-30550486:30550572-30553897:-	-0.698784316	0.023103394
C16orf13;MX:chr16:684797-684889:684956-685612:684797-685281:685340-685612:-	-0.422368138	0.023103394
UBE2C;MX:chr20:44442103-44443023:44443109-44444493:44442103-44444180:44444384-44444493:+	-0.203621893	0.023103394
ZNF778;MX:chr16:89288591-89289565:89289691-89291963:89288591-89291127:89291210-89291963:+	0.166592935	0.023103394
PCBP4;MX:chr3:51995320-51995957:51996104-52001342:51995320-51996826:51996908-52001342:-	0.134693449	0.027460744
SORBS2;MX:chr4:186599976-186605908:186606000-186696381:186599976-186611716:186611765-186696381:-	0.457346703	0.031450449
PAX5;MX:chr9:36840633-36846840:36846926-36923352:36840633-36882001:36882102-36923352:-	0.50680871	0.031686389
GNPDA2;MX:chr4:44719312-44720326:44720427-44728491:44719312-44724101:44724259-44728491:-	-0.226871436	0.032509445
P4HA2;MX:chr5:131531173-131533899:131533964-131534572:131531173-131534014:131534073-131534572:-	0.10292619	0.032509445
DLG1;MX:chr3:196796131-196802708:196802741-196807922:196796131-196803457:196803553-196807922:-	0.124785834	0.032509445
SORBS1;MX:chr10:97127456-97131083:97131184-97135730:97127456-97131741:97131806-97135730:-	0.387401108	0.032509445
NR2F1-AS1;MX:chr5:92747215-92768071:92768166-92889467:92747215-92774409:92774447-92889467:-	0.308745741	0.036713856
GSN;MX:chr9:124043840-124044153:124044231-124064241:124043840-124062343:124062404-124064241:+	0.330419795	0.038333139
PGAP2;MX:chr11:3829545-3832480:3832654-3845500:3829545-3845113:3845365-3845500:+	0.452259247	0.038333139
SORBS2;MX:chr4:186556565-186559233:186559316-186567822:186556565-186560031:186560189-186567822:-	-0.386364049	0.041540067
TCTN1;MX:chr12:111070364-111072201:111072268-111074286:111070364-111072475:111072584-111074286:+	-0.134598706	0.044538616
ANKMY1;MX:chr2:241439982-241446977:241447108-241452219:241439982-241451278:241451418-241452219:-	-0.228638854	0.045020881
OGDH;MX:chr7:44685117-44687043:44687133-44706335:44685117-44687256:44687358-44706335:+	0.202737801	0.045020881
GSN;MX:chr9:124043840-124045597:124045670-124064241:124043840-124062343:124062404-124064241:+	0.425841873	0.045020881
PLAGL1;MX:chr6:144263800-144269122:144269597-144285924:144263800-144281606:144281679-144285924:-	0.63067294	0.04771488

Supplementary Table 10. A list of significant isoform switches (p-value<0.05 & absolute Relative expression median delta>0.1) of event type Retained Intron (RI) found in ESRP1-low versus ESRP-high cell lines using two-sided Wilcoxon test

Event name	Relative expression median delta	Wilcoxon p-value of relative expression
GOLGA8A;RI:chr15:34678826:34678942-34680034:34680153:-	-0.111164525	0.000835477
KCNK7;RI:chr11:65360326:65360681-65360781:65360854:-	-0.649443823	0.002608443
EDEM2;RI:chr20:33734628:33734738-33734955:33735161:-	0.109480022	0.00406519
ZBTB7B;RI:chr1:154975106:154975162-154975319:154975419:+	0.148711404	0.00406519
**BICD2;RI:chr9:95473645:95475743-95477535:95477745:-**	0.76577653	0.00406519
DMAP1;RI:chr1:44679125:44679199-44679381:44679530:+	-0.118309979	0.006154624
PHYKPL;RI:chr5:177658670:177658867-177659493:177659823:-	0.188385714	0.006154624
GYLTL1B;RI:chr11:45944372:45944515-45944609:45944718:+	-0.389167878	0.007526315
LOC285740;RI:chr6:143882304:143882605-143883022:143883188:-	0.812021276	0.007632882
ZNF248;RI:chr10:38117693:38120240-38121953:38122044:-	-0.235256224	0.009166074
ZNRD1;RI:chr6:30029017:30029175-30029292:30029446:+	-0.181600692	0.009166074
PIGO;RI:chr9:35091237:35091288-35092540:35092764:-	0.183379973	0.009166074
HSPBP1;RI:chr19:55791403:55791476-55791586:55791751:-	0.254588603	0.009166074
LINC00623:LINC00869;RI:chr1:144300468:144300755-144301190:144301536:-	0.38338589	0.011117561
SEPT4;RI:chr17:56599097:56599214-56599328:56599462:-	0.13159553	0.014697422
BCKDHB;RI:chr6:81053381:81053529-81055692:81055987:+	-0.229416632	0.016156931
MYLK-AS1;RI:chr3:123304360:123304400-123304494:123304648:+	0.622910077	0.020221587
ZFAND2B;RI:chr2:220071506:220071567-220071692:220071764:+	-0.190281612	0.023103394
NELL2;RI:chr12:45269598:45269683-45270077:45270247:-	-0.404795933	0.02396768
FMO5;RI:chr1:146696487:146696658-146696869:146697230:-	-0.315920528	0.026026074
ZNF154;RI:chr19:58207643:58212513-58212606:58214156:-	-0.546474831	0.027460744
BZRAP1-AS1;RI:chr17:56414563:56414671-56414809:56415040:+	-0.468826659	0.027460744
LINC00623:LINC00869;RI:chr1:144298164:144300755-144301350:144301536:-	-0.402628549	0.027460744
PTGES2;RI:chr9:130887523:130887562-130887655:130887720:-	-0.222695225	0.027460744
ISY1:ISY1-RAB43:RAB43;RI:chr3:128840129:128840335-128840587:128840647:-	-0.206176013	0.027460744
TNFSF12:TNFSF12-TNFSF13:TNFSF13;RI:chr17:7461609:7461692-7462385:7462614:+	-0.150420896	0.027460744
ARHGAP33;RI:chr19:36278070:36278461-36278546:36279724:+	-0.124956562	0.027460744
PML;RI:chr15:74326819:74326871-74327513:74328735:+	-0.108039905	0.027460744
NEK1;RI:chr4:170533171:170533294-170533373:170533778:-	0.118059095	0.027460744
PHKG2;RI:chr16:30768125:30768280-30771440:30772497:+	0.122873081	0.027460744
MDFI;RI:chr6:41606195:41606390-41606477:41606563:+	0.436917842	0.027806895
CSTF3;RI:chr11:33162977:33163308-33163431:33163532:-	0.121510861	0.032509445
OGG1;RI:chr3:9798451:9798500-9798728:9799089:+	0.145419331	0.032509445
C14orf93;RI:chr14:23467636:23467950-23468263:23468365:-	0.379700499	0.032509445
PRRT2;RI:chr16:29824311:29825254-29825654:29827202:+	0.482658936	0.032509445
HMGN3;RI:chr6:79910962:79911443-79911993:79912106:-	0.101720913	0.038333139
ZNF131;RI:chr5:43161351:43161708-43161811:43162033:+	0.115763685	0.038333139
GLYCTK;RI:chr3:52326276:52326585-52327062:52327208:+	0.426321783	0.041563239
COCH;RI:chr14:31343741:31343797-31344122:31344178:+	-0.299762907	0.048452223
ANKMY1;MX:chr2:241439982-241446977:241447108-241452219:241439982-241451278:241451418-241452219:-	-0.228638854	0.045020881
OGDH;MX:chr7:44685117-44687043:44687133-44706335:44685117-44687256:44687358-44706335:+	0.202737801	0.045020881
GSN;MX:chr9:124043840-124045597:124045670-124064241:124043840-124062343:124062404-124064241:+	0.425841873	0.045020881
PLAGL1;MX:chr6:144263800-144269122:144269597-144285924:144263800-144281606:144281679-144285924:-	0.63067294	0.04771488

Supplementary Table 11. A list of significant isoform switches (p-value<0.05 & absolute Relative expression median delta>0.1) of event type Skipping Exon (SE) found in ESRP1-low versus ESRP-high cell lines using two-sided Wilcoxon test

Event name	Relative expression median delta	Wilcoxon p-value of relative expression
AP1B1;SE:chr22:29724884-29725701:29725709-29726367:-	-0.133937491	0.000182811
AP1B1;SE:chr22:29735122-29735743:29735763-29736645:-	-0.133937491	0.000182811
FNIP1;SE:chr5:131044965-131046271:131046354-131052258:-	0.389040809	0.000182811
EXOC7;SE:chr17:74085401-74086410:74086478-74090495:-	0.391331548	0.000182811
TUFT1;SE:chr1:151512902-151534567:151534641-151535061:+	0.391396293	0.000182811
ENAH;SE:chr1:225688772-225692693:225692755-225695653:-	0.657639342	0.000182811
DNM2;SE:chr19:10916643-10919245:10919256-10922940:+	0.559104469	0.000237997
INF2;SE:chr14:105181193-105181621:105181677-105185132:+	0.202449373	0.000308535
NF2;SE:chr22:30077590-30079009:30079053-30090741:+	0.383331827	0.000308535
BBX;SE:chr3:107497366-107508634:107508723-107510087:+	0.144383556	0.000398294
TCF7L2;SE:chr10:114711366-114724315:114724383-114799784:+	0.367312525	0.000398294
BAIAP2;SE:chr17:79082309-79084714:79084759-79089570:+	0.55397938	0.000398294
ANAPC10;SE:chr4:146017263-146018647:146018714-146018840:-	-0.987521475	0.000512005
ACOT9;SE:chrX:23751334-23752458:23752484-23754036:-	-0.734037318	0.000512005
RALGAPA1:RALGAPA1P;SE:chr14:36008896-36017714:36017744-36018316:-	-0.695864708	0.000512005
FGFR1OP2;SE:chr12:27110676-27113448:27113561-27116275:+	-0.396018387	0.000512005
EDRF1;SE:chr10:127414407-127417572:127417673-127417927:+	0.142933282	0.000512005
ZMYND8;SE:chr20:45839542-45841287:45841370-45848909:-	0.147218276	0.000512005
USO1;SE:chr4:76678726-76681747:76681758-76691592:+	0.270316964	0.000512005
USO1;SE:chr4:76715054-76716489:76716509-76720775:+	0.270316964	0.000512005
**CCDC50;SE:chr3:191087825-191092851:191093378-191097948:+**	0.629026305	0.000512005
FLNB;SE:chr3:58124256-58127585:58127623-58128377:+	0.741290951	0.000512005
EXOC7;SE:chr17:74086478-74087224:74087316-74090495:-	-0.548470749	0.000655415
CD46;SE:chr1:207959027-207963598:207963690-207966864:+	-0.347494203	0.000655415
LAS1L;SE:chrX:64752510-64753490:64753615-64754360:-	-0.185716108	0.000655415
SPTAN1;SE:chr9:131353904-131355262:131355321-131356454:+	0.285058842	0.000655415
EXOC7;SE:chr17:74085401-74086410:74086562-74087224:-	0.494788447	0.000655415
CTNND1:TMX2:TMX2-CTNND1;SE:chr11:57529518-57556509:57556627-57558857:+	-0.654129589	0.000835477
PLEKHA1;SE:chr10:124186547-124187792:124187936-124189140:+	-0.214943297	0.000835477
VPS39;SE:chr15:42483758-42484264:42484296-42492094:-	0.200046413	0.000835477
EXOC7;SE:chr17:74085401-74086410:74086478-74087224:-	0.23576799	0.000835477
**LRRFIP2;SE:chr3:37125297-37132958:37133029-37136283:-**	-0.768922653	0.001060553
ACLY;SE:chr17:40049427-40052873:40052902-40054002:-	0.142629253	0.001060553
MBNL1;SE:chr3:152163328-152164493:152164546-152165409:+	0.186962726	0.001060553
ADAM15;SE:chr1:155033308-155034380:155034451-155034721:+	0.216448172	0.001060553
MARK2;SE:chr11:63670630-63671458:63671619-63672258:+	0.358839902	0.001060553
NUMB;SE:chr14:73744001-73745989:73746132-73749067:-	0.420290113	0.001060553
FLNB;SE:chr3:58124256-58127585:58127656-58128377:+	0.471997074	0.001060553
MYOF;SE:chr10:95148911-95152674:95152712-95155899:-	-0.764377125	0.001340641
YBX3;SE:chr12:10856747-10862507:10862713-10865810:-	-0.265680704	0.001340641
APLP2;SE:chr11:129992408-129993507:129993674-129996595:+	0.113567446	0.001340641
RPS24;SE:chr10:79797062-79797723:79797740-79799962:+	0.186548782	0.001340641
TCF12;SE:chr15:57543621-57544619:57544690-57545460:+	0.30439451	0.001340641
RRP12;SE:chr10:99148381-99150180:99150296-99155975:-	0.383398658	0.001340641
MAP3K7;SE:chr6:91246120-91254271:91254351-91256977:-	0.549053122	0.001340641
IMPDH1;SE:chr7:128040593-128040886:128040945-128041069:-	0.631416405	0.001340641
MYH14;SE:chr19:50726606-50727411:50727434-50728842:+	0.2949388	0.001524379
RGL2;SE:chr6:33264892-33266232:33266428-33266647:-	-0.514662652	0.001687636
ZEB1;SE:chr10:31750166-31784708:31784767-31791276:+	-0.441216963	0.001687636
MYO18A;SE:chr17:27409456-27412622:27412666-27413456:-	-0.399404557	0.001687636
MLPH;SE:chr2:238449176-238449445:238449600-238451210:+	0.135263217	0.001687636
DEPDC1;SE:chr1:68947295-68947729:68948580-68949633:-	0.513987002	0.001687636
CTNND1:TMX2:TMX2-CTNND1;SE:chr11:57582972-57583387:57583473-57583769:+	0.709319422	0.001687636
CACNA1G;SE:chr17:48696369-48697044:48697187-48701268:+	0.145897765	0.001707806
CACNA1G;SE:chr17:48696369-48697044:48697187-48699021:+	0.636505139	0.001707806
GIT2;SE:chr12:110377052-110383065:110383154-110385061:-	-0.550557788	0.002115607
ABI1;SE:chr10:27040712-27047991:27048167-27054147:-	-0.531196234	0.002115607
GSE1;SE:chr16:85647004-85667520:85667738-85682158:+	-0.349155388	0.002115607
NF1;SE:chr17:29576137-29579956:29580018-29585362:+	-0.309725616	0.002115607
DCAF6;SE:chr1:167974031-167985019:167985189-167992226:+	-0.201068213	0.002115607
LAS1L;SE:chrX:64744142-64744444:64744494-64744845:-	-0.162669274	0.002115607
SEC31A;SE:chr4:83750211-83763293:83763634-83765539:-	0.171024215	0.002115607
KRAS;SE:chr12:25362845-25368371:25368494-25378548:-	0.207236244	0.002115607
MYEOV;SE:chr11:69062043-69062700:69062962-69063059:+	0.215960215	0.002115607
PCYT2;SE:chr17:79864774-79865080:79865133-79865430:-	0.527557912	0.002115607
RALGPS2;SE:chr1:178858831-178861365:178861442-178863054:+	0.650124788	0.002115607
FAM20A;SE:chr17:66551884-66586265:66586490-66596404:-	0.166093653	0.002199647
MARK3;SE:chr14:103958371-103964839:103964865-103966493:+	-0.764570226	0.002641098
LMO7;SE:chr13:76427524-76429396:76429504-76430642:+	-0.226866311	0.002641098
MLPH;SE:chr2:238427291-238428553:238428672-238434244:+	0.158644369	0.002641098
RHNO1;SE:chr12:2986448-2994429:2994700-2997077:+	-0.587995902	0.003283461
NF2;SE:chr22:30077590-30079009:30079068-30090741:+	0.21309265	0.003283461
TCF7L2;SE:chr10:114919751-114920378:114920450-114925314:+	0.364670266	0.003283461
YAP1;SE:chr11:102076817-102080248:102080295-102094353:+	0.364724759	0.003283461
FGFR1;SE:chr8:38287466-38297824:38297891-38314874:-	0.119521583	0.003823531
TMEM51;SE:chr1:15479280-15536986:15537058-15541391:+	-0.365248143	0.00406519
PRMT2;SE:chr21:48055675-48056351:48056459-48056808:+	-0.210156704	0.00406519
MPRIP;SE:chr17:17083402-17083921:17083983-17088137:+	-0.163790189	0.00406519

Supplementary Table 11. A list of significant isoform switches (p-value<0.05 & absolute Relative expression median delta>0.1) of event type Skipping Exon (SE) found in ESRP1-low versus ESRP-high cell lines using two-sided Wilcoxon test -**Continued**

Event name	Relative expression median delta	Wilcoxon p-value of relative expression
ARNT;SE:chr1:150812130-150814900:150814944-150818739:-	-0.152491891	0.00406519
LRRC14;SE:chr8:145743425-145744133:145744199-145744999:+	0.292190981	0.00406519
TCF7L2;SE:chr10:114917828-114920378:114920450-114925314:+	0.387017841	0.00406519
SMARCC2;SE:chr12:56557549-56558087:56558152-56558432:-	0.500084469	0.00406519
PDGFA;SE:chr7:538211-540068:540136-540753:-	0.592310779	0.00406519
VMO1;SE:chr17:4688954-4689196:4689347-4689453:-	0.495093924	0.004800929
C12orf43;SE:chr12:121444197-121448621:121448729-121448907:-	-0.155176533	0.005012287
RSPH4A;SE:chr6:116949532-116950730:116950865-116951598:+	-0.14832493	0.005012287
ATP1A1-AS1;SE:chr1:116948770-116953883:116953937-116961081:-	-0.140067995	0.005012287
ARPC4:ARPC4-TTLL3:TTLL3;SE:chr3:9855029-9857758:9857886-9859329:+	-0.129793831	0.005012287
PRC1;SE:chr15:91510432-91512309:91512350-91512677:-	-0.121577577	0.005012287
SGOL1;SE:chr3:20212724-20215741:20216547-20218093:-	0.105604152	0.005012287
CDCA7L;SE:chr7:21956512-21979880:21979933-21985399:-	0.111643288	0.005012287
MAP3K4;SE:chr6:161518208-161519310:161519459-161522924:+	0.126824618	0.005012287
CTAGE5;SE:chr14:39788495-39790132:39790260-39796068:+	0.163597656	0.005012287
ZDHHC16;SE:chr10:99213420-99213556:99213603-99214471:+	0.213309104	0.005012287
YAP1;SE:chr11:102076805-102080248:102080295-102094353:+	0.25196656	0.005012287
GPR126;SE:chr6:142703156-142704897:142704980-142711395:+	0.328356498	0.005012287
SLC37A4;SE:chr11:118900274-118900942:118901445-118901559:-	0.38055761	0.005012287
SEC31A;SE:chr4:83750211-83763338:83763634-83765539:-	0.433071205	0.005012287
ZNF177:ZNF559:ZNF559-ZNF177;SE:chr19:9435456-9448472:9448693-9449170:+	-0.408862283	0.005286959
MPP2;SE:chr17:41960723-41975630:41975748-41981798:-	-0.129327508	0.005362948
ST6GALNAC1;SE:chr17:74625793-74633682:74633818-74639590:-	-0.25411736	0.005478553
CTNND1:TMX2:TMX2-CTNND1;SE:chr11:57529518-57556509:57556627-57558966:+	-0.688235171	0.006154624
FOSL1;SE:chr11:65660767-65661485:65661587-65664280:-	-0.370234628	0.006154624
OSBPL3;SE:chr7:24901388-24902819:24902911-24903115:-	-0.315904213	0.006154624
FAM92A1;SE:chr8:94722103-94730903:94731016-94738622:+	-0.259350112	0.006154624
SENP7;SE:chr3:101212812-101219914:101219963-101231935:-	-0.228048895	0.006154624
SRSF3;SE:chr6:36566760-36567598:36568053-36568929:+	0.100465746	0.006154624
ATP11C;SE:chrX:138811121-138813810:138813914-138820075:-	0.11474393	0.006154624
ERBB2IP;SE:chr5:65350779-65364705:65364848-65370852:+	0.235793562	0.006154624
RCCD1;SE:chr15:91498178-91499030:91499113-91499842:+	0.367733393	0.006154624
EXOC1;SE:chr4:56750094-56755054:56755098-56756389:+	0.374289109	0.006154624
CHN2;SE:chr7:29539656-29544360:29544437-29548917:+	-0.424300302	0.006435091
SHOX2;SE:chr3:157820675-157822847:157822918-157823468:-	0.180940001	0.006840858
OLFML3;SE:chr1:114522252-114522597:114522748-114522954:+	-0.440502627	0.007371168
NFYA;SE:chr6:41046903-41048550:41048636-41051785:+	-0.512882452	0.007526315
ZEB1;SE:chr10:31750166-31784705:31784767-31791276:+	-0.403904771	0.007526315
MBNL1;SE:chr3:152165562-152173331:152173366-152174056:+	-0.256266451	0.007526315
BCL6;SE:chr3:187443417-187444519:187444686-187446148:-	-0.123125876	0.007526315
BCS1L;SE:chr2:219524466-219524760:219524968-219525662:+	0.173528528	0.007526315
MAP4K4;SE:chr2:102476326-102477287:102477448-102481392:+	0.18043249	0.007526315
RAI14;SE:chr5:34812313-34813679:34813765-34814688:+	0.292538945	0.007526315
CHRFAM7A;SE:chr15:30672602-30675522:30675585-30679066:-	-0.687996674	0.00808577
NR3C2;SE:chr4:149073764-149075702:149076052-149115897:-	0.203190505	0.008234191
LIMCH1;SE:chr4:41621457-41640949:41640984-41646517:+	-0.370665888	0.008730257
DIO2;SE:chr14:80672078-80672574:80672681-80677594:-	0.294201239	0.008730257
ZNF286A;SE:chr17:15603091-15603449:15603680-15604466:+	-0.357615716	0.009166074
GOLGA4;SE:chr3:37292975-37315027:37315092-37323449:+	-0.320863168	0.009166074
ARHGAP17;SE:chr16:24946960-24950685:24950918-24953308:-	-0.2484795	0.009166074
ABI1;SE:chr10:27040712-27047991:27048164-27054147:-	-0.128391652	0.009166074
INTS9;SE:chr8:28695293-28704264:28704326-28707730:-	0.106548076	0.009166074
MPZL1;SE:chr1:167742605-167745301:167745403-167757057:+	0.125317912	0.009166074
DDR1;SE:chr6:30852487-30853402:30853457-30856465:+	0.173719924	0.009166074
LOC100506990;SE:chr8:12302403-12355092:12355358-12388452:+	0.18615473	0.009166074
FKBP14;SE:chr7:30058739-30059829:30059920-30062281:-	0.187397546	0.009166074
ARHGAP8:PRR5:PRR5-ARHGAP8;SE:chr22:45204318-45204886:45204978-45210552:+	0.215754659	0.009166074
GOLGA4;SE:chr3:37376657-37378634:37378654-37379157:+	0.320863168	0.009166074
GOLGA4;SE:chr3:37396678-37402734:37402796-37407571:+	0.320863168	0.009166074
EML1;SE:chr14:100331983-100341268:100341324-100344822:+	0.458376457	0.009847166
FOXP2;SE:chr7:114174761-114178381:114178438-114210862:+	0.488530244	0.010587137
SYTL2;SE:chr11:85425550-85428526:85428573-85429833:-	-0.689471504	0.011117561
SPHK2;SE:chr19:49123810-49129325:49129619-49130949:+	-0.640419498	0.011117561
IRF3;SE:chr19:50166771-50167700:50167930-50168888:-	-0.242207741	0.011117561
PIGN;SE:chr18:59828618-59829486:59829562-59830764:-	-0.233414343	0.011117561
NRM;SE:chr6:30656719-30657053:30657229-30657824:-	-0.208722334	0.011117561
NEK1;SE:chr4:170429482-170429920:170430003-170458960:-	-0.15884497	0.011117561
GK;SE:chrX:30714800-30715849:30715866-30718531:+	-0.15860476	0.011117561
SLC9A8;SE:chr20:48466217-48467347:48467381-48471975:+	0.126466122	0.011117561
BAZ2B;SE:chr2:160269056-160284452:160284553-160284822:-	0.131498099	0.011117561
ERBB2IP;SE:chr5:65350779-65370852:65371058-65372144:+	0.144157667	0.011117561
MRPL55;SE:chr1:228296019-228296656:228296722-228296962:-	0.346320211	0.011117561
SMPD4;SE:chr2:130930468-130930852:130930927-130932487:-	0.74152181	0.011117561
SERINC5;SE:chr5:79409448-79410337:79410474-79441913:-	0.996763161	0.011117561
CHN2;SE:chr7:29394279-29407548:29407603-29433295:+	0.167448835	0.011159425
DDC;SE:chr7:50571757-50596906:50597040-50605558:-	0.831556937	0.011412036
OGDHL;SE:chr10:50960786-50964822:50964992-50966435:-	-0.511699148	0.01144592
RCAN3;SE:chr1:24841057-24857708:24857881-24861583:+	-0.674475773	0.012334708

Supplementary Table 11. A list of significant isoform switches (p-value<0.05 & absolute Relative expression median delta>0.1) of event type Skipping Exon (SE) found in ESRP1-low versus ESRP-high cell lines using two-sided Wilcoxon test -**Continued**

Event name	Relative expression median delta	Wilcoxon p-value of relative expression
NR5A2;SE:chr1:199997039-200008786:200008923-200012902:+	-0.561096821	0.012429351
ZNF177:ZNF559:ZNF559-ZNF177;SE:chr19:9435456-9448472:9448534-9449170:+	-0.221972955	0.012554919
FGFR1;SE:chr8:38285953-38287200:38287466-38314874:-	0.192833675	0.012554919
FGFR1;SE:chr8:38315052-38318614:38318773-38325190:-	0.241857936	0.012554919
ERICH5;SE:chr8:99076955-99101304:99102257-99105449:+	0.171872711	0.012606357
IFI27;SE:chr14:94577143-94577971:94578119-94581197:+	0.25744471	0.012731364
LOC285740;SE:chr6:143876367-143878668:143878878-143879136:-	-0.799821742	0.012738072
SORBS2;SE:chr4:186599976-186605908:186606000-186696381:-	0.677584414	0.012738072
RPH3AL;SE:chr17:177370-183550:183725-202503:-	-0.426155033	0.013429696
LOXL3;SE:chr2:74763598-74763836:74764055-74776496:-	-0.32583428	0.013429696
SYNRG;SE:chr17:35936516-35937478:35937711-35944756:-	-0.207226193	0.013429696
TCF7L2;SE:chr10:114917828-114918426:114918476-114925314:+	-0.156612688	0.013429696
SPAG9;SE:chr17:49052308-49053224:49053262-49054469:-	-0.114960985	0.013429696
ARMC2;SE:chr6:109175688-109179402:109179474-109190027:+	0.189668733	0.013429696
ELMOD3;SE:chr2:85582293-85582678:85582721-85584090:+	0.220595618	0.013429696
PAX6;SE:chr11:31823324-31823419:31823460-31824252:-	0.340800865	0.013429696
SEC61A2;SE:chr10:12204288-12209752:12209892-12211257:+	0.399493364	0.013429696
ELN;SE:chr7:73459623-73460518:73460547-73461026:+	-0.110862531	0.013554666
LINC00960;SE:chr3:75725031-75725119:75725279-75727984:+	0.345819149	0.014171388
FXYD2:FXYD6:FXYD6-FXYD2;SE:chr11:117714921-117728926:117729001-117747307:-	0.401866641	0.014305878
P2RX2;SE:chr12:133196160-133196265:133196336-133196430:+	0.417530264	0.014305878
FXYD2:FXYD6:FXYD6-FXYD2;SE:chr11:117713496-117714825:117714921-117728926:-	0.422914071	0.014305878
BDNF;SE:chr11:27680132-27680367:27680473-27721711:-	0.268044499	0.014886724
LIMCH1;SE:chr4:41668683-41672735:41672809-41673571:+	0.325153576	0.014886724
TPPP3;SE:chr16:67425020-67426850:67426940-67427283:-	0.102277199	0.014982894
SUGCT;SE:chr7:40535985-40723655:40723732-40789033:+	0.192215565	0.015806775
GPR56;SE:chr16:57662714-57675503:57675620-57684165:+	-0.566395547	0.016156931
CP;SE:chr3:148903233-148904307:148904519-148905839:-	-0.498816963	0.016156931
ABI1;SE:chr10:27044670-27047991:27048164-27054147:-	-0.388858593	0.016156931
NUMB;SE:chr14:73763993-73783098:73783130-73789838:-	-0.283208473	0.016156931
ABCB9;SE:chr12:123424831-123425354:123425542-123428938:-	-0.201768344	0.016156931
TSPAN4;SE:chr11:842915-847201:847300-850288:+	-0.198924878	0.016156931
ARMCX5:ARMCX5-GPRASP2:GPRASP2;SE:chrX:101967480-101968019:101968145-101968710:+	-0.191193325	0.016156931
SNRK;SE:chr3:43328167-43341246:43341307-43344590:+	-0.166984601	0.016156931
HSF2;SE:chr6:122744831-122749048:122749101-122752575:+	-0.149235972	0.016156931
BZRAP1-AS1;SE:chr17:56415040-56423641:56423704-56429096:+	-0.130071598	0.016156931
CPNE1:RBM12;SE:chr20:34220845-34243124:34243266-34246852:-	-0.123039076	0.016156931
BBC3;SE:chr19:47730011-47731415:47731703-47735772:-	0.132788215	0.016156931
DCAF6;SE:chr1:167974031-167992226:167992285-168007609:+	0.159079302	0.016156931
CACNA2D2;SE:chr3:50407811-50410496:50410516-50412168:-	0.19534971	0.016156931
ELK1;SE:chrX:47500874-47509320:47509425-47509822:-	0.205286043	0.016156931
PDE4DIP;SE:chr1:144868172-144871696:144871881-144873877:-	0.206743123	0.016156931
EPB41;SE:chr1:29379824-29385101:29385157-29386934:+	0.207996934	0.016156931
LMF1;SE:chr16:981793-983991:984145-984244:-	0.400131448	0.016156931
SLC37A2;SE:chr11:124955914-124956100:124956156-124958015:+	0.784854439	0.016156931
ZSCAN31;SE:chr6:28295310-28297080:28297555-28303607:-	0.793821561	0.016156931
FLNC;SE:chr7:128489632-128490030:128490128-128490438:+	-0.369421683	0.016762336
PILRA;SE:chr7:99972056-99987511:99987729-99995502:+	0.814575579	0.016762336
MUC1;SE:chr1:155159850-155159931:155160020-155160198:-	-0.850443762	0.017694795
LEF1;SE:chr4:108969907-108984779:108984819-108985492:-	-0.129483655	0.017960478
CACNA1G;SE:chr17:48669453-48672354:48672422-48673923:+	0.609939378	0.018204132
MAGEA2:MAGEA2B;SE:chrX:151883171-151883568:151883646-151884446:+	0.361463773	0.018770972
IRF4;SE:chr6:405130-406766:406899-407455:+	0.184813304	0.019016474
COLEC11;SE:chr2:3660972-3673604:3673682-3685123:+	0.124460842	0.019172485
APITD1:APITD1-CORT:CORT;SE:chr1:10491458-10494714:10494747-10500404:+	-0.757049262	0.019359467
SMAD9;SE:chr13:37439895-37441410:37441520-37446795:-	-0.560871443	0.019359467
PPIL6;SE:chr6:109721373-109724234:109724311-109740390:-	-0.310597616	0.019359467
KGFLP1;SE:chr9:46688114-46698075:46698184-46744177:+	-0.27585062	0.019359467
ACAD11:NPHP3:NPHP3-ACAD11;SE:chr3:132416206-132418197:132418294-132418762:-	-0.233084003	0.019359467
MXRA7;SE:chr17:74676961-74679929:74680009-74681154:-	-0.204772648	0.019359467
POMT1;SE:chr9:134378460-134381501:134381607-134381790:+	-0.166755525	0.019359467
ACSF2;SE:chr17:48538233-48538603:48538731-48538994:+	-0.152444484	0.019359467
IFNLR1;SE:chr1:24484381-24485542:24485672-24485964:-	-0.150057862	0.019359467
RTN3;SE:chr11:63472379-63486174:63488504-63517463:+	-0.124697552	0.019359467
CKLF:CKLF-CMTM1:CMTM1;SE:chr16:66592251-66597025:66597120-66599789:+	-0.120860349	0.019359467
LTBP1;SE:chr2:33540336-33567905:33568030-33572434:+	0.146440996	0.019359467
ARHGAP12;SE:chr10:32120728-32128565:32128639-32132389:-	0.207465981	0.019359467
THTPA;SE:chr14:24025552-24025952:24026243-24027904:+	0.279453776	0.019359467
INPP5J;SE:chr22:31520892-31521819:31521996-31522362:+	0.412417354	0.019359467
BCL2L12;SE:chr19:50172194-50172281:50172421-50176955:+	0.831384085	0.019359467
TMPRSS4;SE:chr11:117985642-117985853:117985995-117988020:+	0.148721737	0.019517481
NCAM1;SE:chr11:113092047-113101918:113101995-113102367:+	0.429883257	0.019630657
TMEM130;SE:chr7:98457961-98460718:98461023-98467400:-	-0.21719077	0.020136752
CASP5;SE:chr11:104872928-104874001:104874110-104877810:-	0.400729763	0.020136752
SLC15A3;SE:chr11:60707110-60708594:60708762-60709507:-	-0.187089816	0.02121768
CHN2;SE:chr7:29234606-29332541:29332579-29394241:+	-0.285104659	0.021966365
PDE4B;SE:chr1:66379039-66384280:66384518-66713143:+	0.446621474	0.022740296
TBC1D23;SE:chr3:100029386-100030677:100030721-100034943:+	-0.328198396	0.023103394

Supplementary Table 11. A list of significant isoform switches (p-value<0.05 & absolute Relative expression median delta>0.1) of event type Skipping Exon (SE) found in ESRP1-low versus ESRP-high cell lines using two-sided Wilcoxon test -**Continued**

Event name	Relative expression median delta	Wilcoxon p-value of relative expression
ARPC4:ARPC4-TTLL3:TTLL3;SE:chr3:9859443-9860239:9860604-9862230:+	-0.177704087	0.023103394
QTRTD1;SE:chr3:113775711-113775846:113775953-113784084:+	-0.143569249	0.023103394
NAE1;SE:chr16:66860486-66860580:66860683-66864750:-	-0.114648372	0.023103394
LINC01128;SE:chr1:764484-783034:783186-787307:+	0.104692283	0.023103394
ANAPC10;SE:chr4:146017263-146018840:146018912-146019272:-	0.108536715	0.023103394
FAM13A;SE:chr4:89653349-89658623:89658706-89660181:-	0.153254856	0.023103394
LOC101929147;SE:chr1:119683416-119689576:119689692-119693188:+	0.213942095	0.023103394
ADD3;SE:chr10:111890244-111892063:111892158-111893084:+	0.386562447	0.023103394
PLCB4;SE:chr20:9454012-9457364:9457400-9459568:+	0.400634488	0.023103394
TMEM106A;SE:chr17:41363941-41364224:41364413-41365040:+	0.69769942	0.023103394
FPR1;SE:chr19:52250258-52253456:52253506-52255067:-	-0.261597146	0.023342202
WBP5;SE:chrX:102611534-102612011:102612089-102612543:+	0.135502281	0.023919348
LDLRAD1;SE:chr1:54477953-54479909:54480037-54483049:-	-0.581260181	0.02396768
MAL;SE:chr2:95691630-95713704:95713871-95715326:+	0.781497093	0.025347319
CARD16;SE:chr11:104912446-104914210:104914272-104915119:-	-0.187322168	0.025952456
ZNF254;SE:chr19:24270139-24288742:24288868-24289350:+	-0.28360708	0.026387678
AKR1C2;SE:chr10:5046206-5049599:5049698-5060092:-	0.144578228	0.026387678
SEMA7A;SE:chr15:74710310-74710609:74710650-74711142:-	0.285163112	0.026387678
SLC44A4;SE:chr6:31842297-31842498:31842623-31842706:-	0.364906335	0.026856696
DDC;SE:chr7:50597040-50605558:50605677-50611583:-	0.614903539	0.026856696
SORBS1;SE:chr10:97131184-97131741:97131806-97135730:-	-0.974311941	0.027460744
JAM3;SE:chr11:134010665-134014136:134014288-134014687:+	-0.77480879	0.027460744
SYNC;SE:chr1:33147461-33149611:33149690-33149859:-	-0.38524637	0.027460744
NDEL1;SE:chr17:8363478-8366638:8366672-8370248:+	-0.337628326	0.027460744
CD99L2;SE:chrX:149963762-149963891:149963959-149999704:-	-0.301250958	0.027460744
ZNF133;SE:chr20:18269248-18286312:18286451-18286942:+	-0.266591486	0.027460744
LYRM1;SE:chr16:20912211-20913808:20914039-20926878:+	-0.20359349	0.027460744
CBWD1;SE:chr9:135030-146102:146158-152034:-	-0.134109922	0.027460744
ARHGAP33;SE:chr19:36266566-36268547:36268644-36268726:+	-0.124956562	0.027460744
KIF21A;SE:chr12:39720126-39724044:39724064-39724548:-	-0.107917926	0.027460744
REPS1;SE:chr6:139242261-139247538:139247618-139251114:-	0.128605716	0.027460744
ZFYVE27;SE:chr10:99497078-99498234:99498406-99504486:+	0.167898527	0.027460744
SDCBP2-AS1;SE:chr20:1353822-1355128:1355342-1357442:+	0.215355232	0.027460744
GLYCTK;SE:chr3:52321892-52324320:52324735-52324976:+	0.226676304	0.027460744
PRR3;SE:chr6:30525227-30525927:30525989-30529611:+	0.230298734	0.027460744
NFATC2;SE:chr20:50007988-50015180:50015267-50048604:-	0.247437428	0.027460744
ALDH1L1;SE:chr3:125831723-125833400:125833499-125836848:-	-0.152250532	0.027890529
ALDH1L1;SE:chr3:125849136-125850227:125850377-125854378:-	-0.152250532	0.027890529
COL2A1;SE:chr12:48392214-48393702:48393908-48398020:-	-0.395608335	0.028351037
EYA4;SE:chr6:133767892-133769249:133769317-133777694:+	0.135308305	0.028351037
C14orf132;SE:chr14:96505864-96517461:96517559-96552848:+	0.265304947	0.028459737
SORBS2;SE:chr4:186696520-186815451:186815527-186877350:-	0.603795715	0.029049022
CLEC7A;SE:chr12:10278047-10279170:10279307-10280346:-	-0.259016218	0.030368472
CLEC7A;SE:chr12:10271189-10275844:10275962-10277896:-	0.245335935	0.030368472
MAPT;SE:chr17:44074030-44087676:44087768-44091609:+	0.199341994	0.030499933
VMO1;SE:chr17:4688954-4689252:4689347-4689453:-	0.710982154	0.030708373
CLIC5;SE:chr6:45882284-45888388:45888426-45905142:-	0.459632764	0.030753561
BDNF;SE:chr11:27680132-27681703:27681819-27695607:-	0.247329408	0.031228617
KIF1A;SE:chr2:241689964-241696736:241697011-241697777:-	0.26000226	0.031228617
KIF1A;SE:chr2:241710521-241711987:241712013-241712531:-	0.26000226	0.031228617
TNFSF10;SE:chr3:172224709-172227007:172227111-172232651:-	0.443710942	0.031228617
PAX5;SE:chr9:36840633-36882001:36882102-36923352:-	-0.523549019	0.031686389
SEPT4;SE:chr17:56604339-56616473:56616569-56618027:-	0.239098545	0.031794861
HSD11B1L;SE:chr19:5685130-5686427:5686538-5686911:+	-0.684858542	0.032509445
DEDD2;SE:chr19:42719404-42720832:42721197-42724226:-	-0.216895427	0.032509445
GAB1;SE:chr4:144361535-144378833:144378922-144380538:+	-0.215261631	0.032509445
CCSER2;SE:chr10:86237420-86259631:86259715-86273205:+	-0.193162871	0.032509445
ZNF107;SE:chr7:64139714-64150777:64150903-64151626:+	-0.185294955	0.032509445
SREBF1;SE:chr17:17723835-17726832:17726921-17740041:-	-0.173474891	0.032509445
CLK1;SE:chr2:201724469-201724848:201724938-201725961:-	-0.162021012	0.032509445
PSMG4;SE:chr6:3263993-3264443:3264559-3267825:+	-0.157277148	0.032509445
LRRC42;SE:chr1:54412222-54413461:54413494-54417659:+	-0.145199976	0.032509445
LIN9;SE:chr1:226475498-226483543:226483647-226485420:-	-0.134556247	0.032509445
EXOC7;SE:chr17:74085401-74087224:74087316-74090495:-	-0.133556115	0.032509445
ETV4;SE:chr17:41622735-41622926:41623036-41623229:-	-0.119555966	0.032509445
DNM1;SE:chr9:131013219-131015380:131015416-131016933:+	0.133837093	0.032509445
LRRC75A;SE:chr17:16347445-16351159:16351274-16365572:-	0.143571249	0.032509445
HSH2D;SE:chr19:16259685-16262257:16262388-16263362:+	0.149045201	0.032509445
PPP3CC;SE:chr8:22390531-22396982:22397011-22398128:+	0.162024012	0.032509445
TSNAXIP1;SE:chr16:67848304-67855002:67855128-67858486:+	0.171377754	0.032509445
P4HA2;SE:chr5:131554337-131562779:131562914-131563483:-	0.188413144	0.032509445
INPP4A;SE:chr2:99163157-99165418:99165432-99169249:+	0.244058254	0.032509445
ZNF75A;SE:chr16:3362768-3363049:3363175-3366912:+	0.245471679	0.032509445
ATG12;SE:chr5:115173461-115176194:115176309-115176515:-	0.324100928	0.032509445
RHOBTB1;SE:chr10:62671310-62695971:62696042-62701562:-	-0.146658099	0.032524181
SLC44A3;SE:chr1:95286034-95286505:95286612-95290049:+	0.21303629	0.032524181
FKBP6;SE:chr7:72742695-72743363:72743452-72744153:+	-0.107147452	0.033006258
TUSC3;SE:chr8:15605974-15615300:15615364-15621712:+	0.326068566	0.033474065

Supplementary Table 11. A list of significant isoform switches (p-value<0.05 & absolute Relative expression median delta>0.1) of event type Skipping Exon (SE) found in ESRP1-low versus ESRP-high cell lines using two-sided Wilcoxon test -**Continued**

Event name	Relative expression median delta	Wilcoxon p-value of relative expression
ELF5;SE:chr11:34511606-34515026:34515259-34527176:-	0.382581709	0.033474065
ZFR2;SE:chr19:3852612-3855400:3855557-3868963:-	-0.348504669	0.033894854
PLAGL1;SE:chr6:144285955-144287295:144287367-144290044:-	-0.230546316	0.033894854
CASP12;SE:chr11:104761250-104761897:104762131-104763058:-	0.406013034	0.033894854
PDE9A;SE:chr21:44073993-44106302:44106372-44119078:+	0.226993202	0.034610558
CADM1;SE:chr11:115049495-115061608:115061661-115069126:-	-0.253994656	0.034808479
CAPS2;SE:chr12:75692560-75692651:75692746-75693617:-	-0.312902362	0.036713856
EMR1;SE:chr19:6896552-6897160:6897315-6897439:+	-0.21087184	0.036713856
MAGEA2:MAGEA2B;SE:chrX:151884565-151885137:151885234-151885385:+	0.275057654	0.036713856
KCNH7;SE:chr2:163361167-163369164:163369184-163374240:-	0.925610851	0.036713856
CACNA1G;SE:chr17:48697187-48699021:48699155-48701268:+	0.127094199	0.037573552
TP73;SE:chr1:3646012-3646564:3646712-3647491:+	0.134108394	0.037573552
C4orf26;SE:chr4:76481794-76488445:76488555-76489324:+	0.999973064	0.037635314
ZNF286A;SE:chr17:15603091-15603522:15603680-15604466:+	-0.505816123	0.038333139
FCHO1;SE:chr19:17865170-17865401:17865490-17865927:+	-0.37816285	0.038333139
ANKLE1;SE:chr19:17394693-17394898:17395075-17396240:+	-0.295002789	0.038333139
GNPDA2;SE:chr4:44719312-44720326:44720427-44724101:-	-0.265541514	0.038333139
TIMM23B;SE:chr10:51371646-51372579:51372702-51374370:+	-0.206948539	0.038333139
TACC2;SE:chr10:123989001-123989866:123989955-123996910:+	-0.192805701	0.038333139
ASAP1;SE:chr8:131370389-131373916:131374017-131414131:-	-0.15149664	0.038333139
TCF7L2;SE:chr10:114918476-114919679:114919751-114925314:+	-0.138290391	0.038333139
TRIM3;SE:chr11:6486962-6494314:6494478-6495021:-	-0.132798361	0.038333139
BORA;SE:chr13:73303231-73305419:73305525-73309098:+	-0.131898954	0.038333139
RNF146;SE:chr6:127601485-127603432:127603540-127607195:+	-0.130931181	0.038333139
KIAA1958;SE:chr9:115337531-115380151:115380234-115407930:+	-0.10311362	0.038333139
EYA3;SE:chr1:28323889-28324795:28324862-28326471:-	-0.100031798	0.038333139
NUMA1;SE:chr11:71721900-71723447:71723488-71723941:-	0.102862586	0.038333139
PXK;SE:chr3:58398690-58409188:58409221-58410479:+	0.103695686	0.038333139
TRA2A;SE:chr7:23561459-23561751:23562051-23571408:-	0.114377237	0.038333139
FAM86B3P;SE:chr8:8090396-8092005:8092106-8093338:+	0.117094576	0.038333139
FAM86B3P;SE:chr8:8093471-8093554:8093821-8094648:+	0.117094576	0.038333139
SYT7;SE:chr11:61300596-61313503:61313727-61318856:-	0.143382318	0.038333139
MEF2B:MEF2BNB:MEF2BNB-MEF2B;SE:chr19:19256831-19257082:19257193-19257364:-	0.162960964	0.038333139
ABL2;SE:chr1:179100616-179102447:179102509-179198376:-	0.182985595	0.038333139
KIFC3;SE:chr16:57792821-57793037:57793065-57793640:-	0.19246881	0.038333139
IRAK4;SE:chr12:44162075-44165023:44165168-44165983:+	0.203708767	0.038333139
ZWILCH;SE:chr15:66797729-66801084:66801230-66806326:+	0.254052868	0.038333139
GSG1;SE:chr12:13241843-13242142:13242210-13243437:-	-0.310165846	0.038867104
HNF4A;SE:chr20:42984493-43019098:43019319-43034698:+	-0.267646398	0.038999953
TOPORS-AS1;SE:chr9:32552477-32565395:32565626-32566389:+	0.243429875	0.039211045
GPR132;SE:chr14:105523617-105524275:105524364-105531716:-	-0.104912059	0.039318369
VMO1;SE:chr17:4688954-4689232:4689347-4689453:-	0.744675924	0.039318369
DIO2;SE:chr14:80669631-80671945:80672078-80672574:-	0.380702634	0.039351837
MAPK10;SE:chr4:87023185-87024339:87024397-87028376:-	-0.318583491	0.040423979
SPRR3;SE:chr1:152974269-152975079:152975162-152975478:+	-0.228550802	0.040423979
KIAA0226L;SE:chr13:46946732-46952025:46952140-46961269:-	0.313489102	0.040423979
BAGE:BAGE2:BAGE3:BAGE4:BAGE5;SE:chr21:11026839-11029598:11029717-11038728:-	0.480048675	0.041226833
PTPRD;SE:chr9:8526644-8527345:8527353-8528591:-	-0.159714479	0.043308143
SPATA9;SE:chr5:94999752-95010390:95010492-95011116:-	-0.130414594	0.043308143
CSE1L-AS1;SE:chr20:47657147-47659687:47659909-47662397:-	1	0.043308143
SAMD14;SE:chr17:48191670-48191721:48191804-48192928:-	0.462205934	0.043311496
BEAN1;SE:chr16:66461334-66471498:66471604-66503505:+	-0.347926156	0.043975196
CEACAM1;SE:chr19:43015072-43015728:43015780-43016502:-	-0.268829877	0.044538616
SLC26A11;SE:chr17:78194295-78194718:78194914-78195347:+	0.380940804	0.044538616
NDUFV2-AS1;SE:chr18:9121454-9132221:9132396-9133389:-	0.668333609	0.044538616
UNC93A;SE:chr6:167709749-167711433:167711558-167717407:+	0.537122688	0.044609718
TTC23;SE:chr15:99768937-99775193:99775285-99781570:-	-0.225615301	0.045020881
ECHDC1;SE:chr6:127648289-127651954:127652175-127664481:-	-0.18725056	0.045020881
TMEM126B;SE:chr11:85339732-85342189:85342360-85342731:+	-0.182686743	0.045020881
MVK;SE:chr12:110017751-110019200:110019355-110023827:+	-0.114805082	0.045020881
PRMT5;SE:chr14:23397420-23397706:23397824-23398376:-	-0.110380562	0.045020881
ZNF142;SE:chr2:219515249-219516424:219516545-219520873:-	-0.1096334	0.045020881
C14orf80;SE:chr14:105959071-105960172:105960270-105963694:+	0.133605244	0.045020881
RAP1GAP2;SE:chr17:2861088-2865964:2866008-2866724:+	0.14396076	0.045020881
MARK2;SE:chr11:63673586-63675732:63675776-63676349:+	0.173220034	0.045020881
RASGRP1;SE:chr15:38794622-38795473:38795577-38798041:-	0.181395785	0.045020881
PPP3CB;SE:chr10:75198178-75199630:75199659-75204483:-	0.212672405	0.045020881
TMEM106A;SE:chr17:41363941-41364246:41364413-41365040:+	0.216890849	0.045020881
SORBS1;SE:chr10:97106209-97110966:97111133-97114639:-	0.220183331	0.045020881
RGL1;SE:chr1:183605636-183666538:183666647-183711261:+	0.236450165	0.045020881
COPZ1;SE:chr12:54718965-54734247:54734399-54735990:+	0.570368402	0.045020881
PRDX3;SE:chr10:120933384-120933963:120934104-120938267:-	0.902376823	0.045020881
C8orf37-AS1;SE:chr8:96281118-96313350:96313420-96444854:+	-0.847021373	0.045500264
GPR56;SE:chr16:57673582-57675503:57675620-57684165:+	0.182572979	0.046539581
SYTL5;SE:chrX:37865921-37890253:37890343-37892787:+	0.16096463	0.047761243
SLC4A9;SE:chr5:139745163-139745419:139745607-139745736:+	-0.19047349	0.048398223
HHAT;SE:chr1:210560925-210573812:210574006-210577808:+	-0.223042126	0.04841124
CADM2;SE:chr3:85008819-85775672:85775698-85851197:+	0.504819109	0.049534613
LINC01518;SE:chr10:43181464-43186024:43186140-43186983:-	0.926619151	0.049534613
